# QTFPred: robust high-performance quantum machine learning modeling that predicts main and cooperative transcription factor bindings with base resolution

**DOI:** 10.1093/bib/bbaf604

**Published:** 2025-11-26

**Authors:** Taichi Matsubara, Shuto Machida, Samuel Papa Kwesi Owusu, Akihiro Asakura, Hiroki Hashimoto, Masanori Matsuoka, Masao Nagasaki

**Affiliations:** Division of Biomedical Information Analysis, Medical Research Center for High Depth Omics, Medical Institute of Bioregulation, Kyushu University, Fukuoka 812-8582, Japan; BlueMeme Inc., Nishikicho 3-20, Kandanishiki-cho, Chiyoda-ku, Tokyo 101-0054, Japan; Division of Biomedical Information Analysis, Medical Research Center for High Depth Omics, Medical Institute of Bioregulation, Kyushu University, Fukuoka 812-8582, Japan; BlueMeme Inc., Nishikicho 3-20, Kandanishiki-cho, Chiyoda-ku, Tokyo 101-0054, Japan; Division of Biomedical Information Analysis, Medical Research Center for High Depth Omics, Medical Institute of Bioregulation, Kyushu University, Fukuoka 812-8582, Japan; Division of Biomedical Information Analysis, Medical Research Center for High Depth Omics, Medical Institute of Bioregulation, Kyushu University, Fukuoka 812-8582, Japan; Division of Biomedical Information Analysis, Medical Research Center for High Depth Omics, Medical Institute of Bioregulation, Kyushu University, Fukuoka 812-8582, Japan; Division of Biomedical Information Analysis, Medical Research Center for High Depth Omics, Medical Institute of Bioregulation, Kyushu University, Fukuoka 812-8582, Japan; BlueMeme Inc., Nishikicho 3-20, Kandanishiki-cho, Chiyoda-ku, Tokyo 101-0054, Japan; Division of Biomedical Information Analysis, Medical Research Center for High Depth Omics, Medical Institute of Bioregulation, Kyushu University, Fukuoka 812-8582, Japan; Center for Genomic Medicine, Graduate School of Medicine, Kyoto University, Kyoto 606-8507, Japan

**Keywords:** quantum computation, transcription factor, deep learning, quantum circuit learning, quantum convolutional neural network, cooperative motif prediction

## Abstract

Deep learning has become an essential tool for identifying transcription factor (TF) binding sites, yet conventional approaches often struggle with limited training data for specific TFs. Here, we introduce QTFPred (Quantum-based TF Predictor), a quantum-classical hybrid framework that integrates quantum convolutional layers within neural networks to predict TF binding at base resolution. By leveraging the exponential feature space offered by quantum circuits and training from scratch via GPU simulation, QTFPred achieves robust performance even in data-sparse scenarios. In benchmarks on 49 Encyclopedia of DNA elements ChIP-seq datasets, QTFPred delivered state-of-the-art accuracy in 92% of binary prediction and 96% of signal prediction tasks, outperforming conventional models in precision and stability. Moreover, the method reveals underlying TF motif representations, offering insights into cooperative binding mechanisms. These results highlight the potential of quantum machine learning to overcome the limitations of traditional deep learning in genomics modeling.

## Introduction

Transcription factors (TFs) regulate gene expression by binding to specific genomic sequence motifs, called transcription factor binding sites (TFBS), with mutations in these sites associated with various diseases [1–4]. The dynamic spatial–temporal patterns and cooperative actions of TFs are critical for proper development [5–7] and create binding specificities different from individual factors [8]. Accurately predicting TFBS is essential for understanding gene regulation mechanisms and their implications in development and disease.

Prior to deep learning approaches, computational TFBS prediction relied primarily on traditional position weight matrices (PWMs) and support vector machine (SVM) [9]. PWMs became the most widely adopted approach, providing interpretable models that quantify the contribution of each nucleotide at each motif position [10, 11]. Despite their effectiveness and biological interpretability, PWMs assume positional independence within binding sites, potentially limiting their ability to capture interdependent nucleotide relationships [12]. SVM-based approaches emerged as an alternative, leveraging k-mer frequency patterns to distinguish bound from unbound sequences and demonstrating superior performance compared with PWMs in several benchmarking studies [13, 14]. However, SVM-based models face constraints in modeling longer motifs due to computational limitations with k-mer length and overfitting issues with increased parameter complexity [9].

Recent advances in deep learning have improved TFBS prediction, evolving from binary classification methods like DeepBind [15–17] to base resolution modeling where predictions are made at each nucleotide position. Examples include FCNA [18], a fully convolutional network (FCN) making single-base predictions, and BPNet [19], which uses dilated CNNs to extract regulatory motifs and enable discovery of cis-regulatory grammar governing TF interactions with nucleotide-level precision.

More recently, Transformer-based architectures have emerged as powerful alternatives to CNNs for genomic sequence modeling, achieving state-of-the-art performance across various TFBS detection tasks. Models such as DNABERT [20], Enformer [21], and the Nucleotide Transformer family [22] leverage self-attention mechanisms to capture long-range dependencies in DNA sequences more effectively than traditional convolutional approaches, enabling better modeling of distal regulatory interactions and complex genomic contexts. While these Transformer-based models demonstrate superior performance in capturing sequence-level patterns and regulatory relationships, they require substantial computational resources for extensive pretraining.

**Figure 1:**
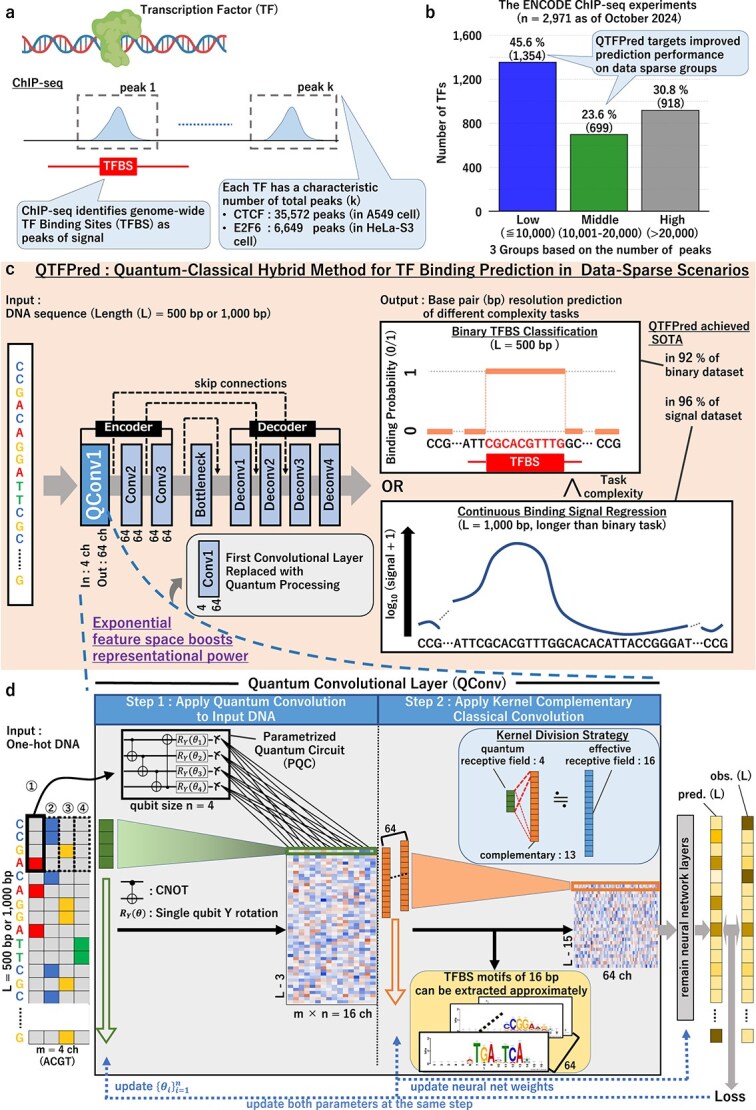
Overview of QTFPred. (a) ChIP-seq methodology for identifying TFBS, where the technique identifies genome-wide binding sites as peaks of signal, with each TF having a characteristic pattern and number of binding regions, (b) distribution of TFs across 2971 ENCODE ChIP-seq experiments (as of October 2024), categorized into three groups based on peak counts: low ($\leq $10 000 peaks), middle (10 001–20 000 peaks), and high (>20 000 peaks), (c) QTFPred architecture overview, showing the model takes DNA sequences as input and produces base resolution predictions for either binary TFBS classification (500 bp sequences) or continuous binding signal regression (1000 bp sequences), and featuring an encoder–decoder structure with skip connections, where the first conventional convolutional layer is replaced with quantum processing (QConv1), and (d) detailed structure of the QConv layer, where the process involves two steps: (1) applying quantum convolution to input DNA using a PQC with 4 qubits and (2) applying kernel complementary classical convolution, while the Kernel Division Strategy combines a quantum receptive field of 4 with a complementary field of 13 to achieve an effective receptive field of 16, approximating the extraction of TFBS motifs of 16 bp.

Generally, it is well established that the performance of deep learning models improves with increasing training data [23, 24]. In TF binding prediction, the training data size depends on the number of high-quality ChIP-seq peaks available (Fig. 1a). However, many TFs yield only a limited number of binding peaks [23]. For instance, an analysis of the Encyclopedia of DNA Elements (ENCODE) ChIP-seq experiments [25] (as of October 2024) indicates that 45.6% of experiments contain <10 000 peaks (Fig. 1b), highlighting a persistent challenge in training robust models with sparse data [23].

Quantum computing offers an alternative approach to classical machine learning by harnessing qubits—units that can exist in a superposition of 0 and 1 and exhibit entanglement—which enable exploring larger feature spaces [26–29]. Quantum machine learning leverages these unique quantum properties to enhance pattern recognition, with methods such as quantum circuit learning (QCL) enabling supervised learning analogous to neural networks [30].

Previous quantum approaches to TF binding prediction [31] used quantum annealing on D-Wave systems [32, 33] to address binding classification via quadratic unconstrained binary optimization (QUBO). However, this approach was limited by quantum annealing platform constraints, static feature representations predetermined by the QUBO function rather than learned from data, processable DNA sequence length of only about 10 bp, and narrow TF coverage (only four types). Importantly, no quantum method has yet demonstrated base-resolution TF binding prediction capabilities.

We implemented QCL-based quantum machine learning via GPU simulation to overcome these limitations, enabling base-resolution TF binding prediction across longer sequences (1000 bp) and diverse TFs. This study introduces QTFPred (Quantum-based TF Predictor), a quantum-classical hybrid method for efficient and robust prediction of TF binding events. QTFPred is trained end-to-end and evaluated on binary classification and signal prediction tasks using 49 ChIP-seq datasets from three ENCODE cell lines. Our benchmarking shows QTFPred achieves state-of-the-art performance in 92% of binary classification datasets and 96% of signal prediction datasets, maintaining high performance even with limited peak data (<10 000 peaks), such as TAF1 (4093 peaks) and TCF12 (4861 peaks) in the MCF7 cell line, without requiring transfer learning [23].

Furthermore, QTFPred identifies both primary TF binding events and cooperative TF interactions in a cell line-dependent manner. By analyzing motif co-occurrence patterns, it provides insights into TF collaboration within specific cellular contexts. For example, our analysis revealed a previously underappreciated E2F6–MAX functional relationship in HeLa cells, with specific spatial constraints (36 bp and 99 bp distances) governing their cooperative binding.

## Results

### Overview of quantum-based transcription factor predictor architecture

QTFPred is a quantum-classical hybrid model constructed to predict TF binding events at the base resolution using DNA sequences as input (Fig. 1c). By combining a quantum convolutional (QConv) layer with a FCN-based encoder, QTFPred leverages quantum computation’s ability to explore higher-dimensional feature spaces and thus potentially improve prediction accuracy compared to conventional CNNs.

QTFPred has the following main characteristics:

1. QTFPred replaces the encoder’s first convolutional layer with a QConv layer (Fig. 1c and d). This design enables parameter search in exponentially large Hilbert space during the initial feature extraction step.
2. Because QTFPred is based on the QCL framework proposed by Mitarai *et al*. [30], it can be trained in a supervised manner using gradient descent.
3. QTFPred supports both binary binding-site prediction and binding-signal regression at the base resolution, enabling the flexible application to various TF binding analyses.

The QCL framework enables end-to-end training of quantum-classical hybrid models by encoding classical information into quantum state vectors, which are the information format processable by quantum circuits, processing through parameterized quantum circuits (PQC), and measuring to produce classical outputs. PQC training involves calculating loss function gradients with respect to variational parameters (rotation gate angles) and updating accordingly. QConv layer applies this process to one-hot encoded DNA sequences, extracting local sequence features relevant to TF binding.

To achieve optimal TF binding motif detection while leveraging quantum computational advantages, we designed QTFPred with a target receptive field of 16 bp, based on two critical considerations. First, this length encompasses most TF binding motifs, as 96.5% of JASPAR2024 motifs are 16 bp or shorter (Supplementary Fig. S25), ensuring comprehensive motif coverage. Second, matching the 16 bp kernel size of the reference classical models (FCNsignal and FCNA) enables direct performance comparison and isolation of pure quantum enhancement effects through architectural substitution rather than confounding structural differences. However, simulating a 16-qubit quantum circuit is computationally prohibitive, necessitating our Kernel Division Strategy (Fig. 1d). This strategy partitions the 16 bp receptive field into a 4-qubit PQC ($2^{4} = 16$D feature space) for initial complex feature extraction, followed by a complementary 13 bp classical convolutional layer for spatial context integration. This division enables practical quantum simulation while preserving the target receptive field and maintaining architectural equivalence with classical baselines for rigorous comparative evaluation.

We assessed QTFPred on ENCODE datasets using two prediction tasks with different complexities:

1. Binary classification distinguishes between TF-bound (value one) and unbound (value zero) regions for each base in 500 bp DNA sequences.
2. Signal prediction estimates continuous ChIP-seq binding intensities across 1000 bp sequences, a more complex task requiring real-valued intensity capture, noise handling in continuous labels, and processing longer sequences in a higher-dimensional output space.

In the following sections, we evaluate QTFPred’s performance in binary classification and signal regression, assess its computational resources, demonstrate how quantum convolution aids in handling signal complexity, and show how it performs motif representation learning while uncovering cooperative regulatory interactions—a novel approach to understanding transcriptional regulation.

### Performance of binary transcription factor binding site classification

We analyzed 49 ChIP-seq samples from three human cell lines: epithelial cell line from human lung (A549: 15 TFs), B-lymphoblastoid cell line from human blood (GM12878: 20 TFs), and epithelial cell line from human breast (MCF7: 14 TFs). The list of TFs and their peak counts is provided in the Supplementary Table S1. This broad sampling captures the heterogeneity of TF families, providing a solid foundation for evaluating diverse TFs. We benchmarked QTFPred against a basic FCN and its state-of-the-art variant (FCNA) using the Intersection over Union (IOU) metric (Supplementary Method S4), which quantifies the overlap between predicted and experimental TFBS regions. Hyperparameter tuning was conducted on an independent dataset of four TFs from A549 to prevent data leakage. All subsequent experimental evaluations were performed across 10 independent trials to ensure robust performance assessment.

**Figure 2:**
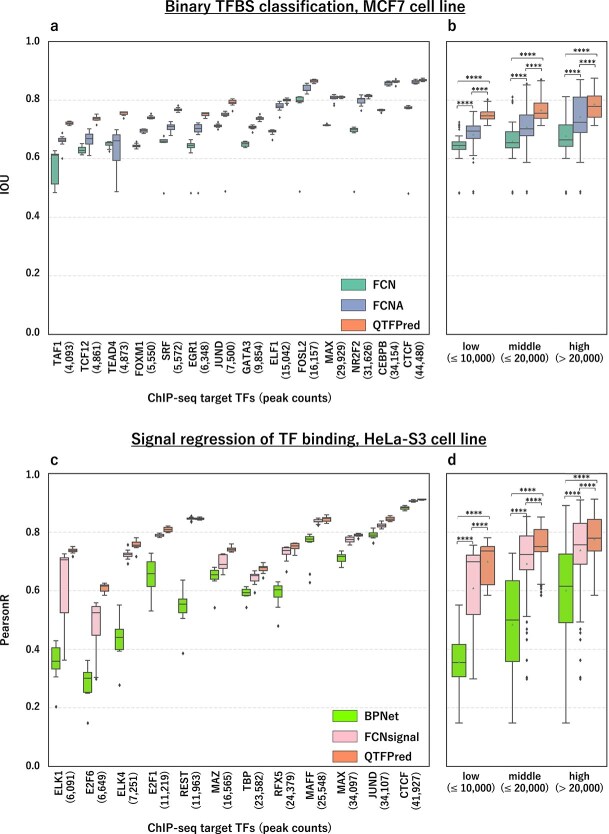
QTFPred outperforms state-of-the-art methods in both binary TFBS classification and signal prediction tasks, particularly for TFs with limited training data, as shown by (a) the performance comparison of QTFPred (right) against FCN (left) and FCNA (middle) models for binary TFBS prediction of 14 TFs in the MCF7 cell line, ordered by increasing peak count, where QTFPred consistently demonstrates superior IOU scores across all TFs, with the most substantial improvements observed for TFs with fewer binding peaks, with box plots represent results from 10 independent trials, and (b) the cumulative distribution of IOU scores grouped by peak count category (low, $\leq $10 000; middle, >10 000 to $\leq $20 000; high, >20 000), and further illustrated by (c) the performance comparison of QTFPred (right) against BPNet (left) and FCNsignal (middle) models for signal prediction of 12 TFs in the HeLa-S3 cell line, ordered by increasing peak count, where QTFPred consistently achieves higher Pearson correlation coefficients across all TFs, with substantial improvements for TFs with limited binding regions, with box plots represent results from 10 independent trials, and (d) the cumulative distribution of Pearson correlation scores grouped by peak count category.

Performance improved with increasing peak size across all methods in the benchmark results for MCF7 TFs, sorted by peak size (Fig. 2a). For example, TAF1 with 4093 regions showed scores of FCN 0.58, FCNA 0.66, QTFPred 0.77; CTCF with 44 480 regions demonstrated FCN 0.75, FCNA 0.86, QTFPred 0.87. QTFPred consistently outperformed FCN and FCNA, establishing a clear hierarchy (FCN < FCNA < QTFPred) across all tested TFs.

To analyze peak size influence, we categorized TFs into three groups: low ($\leq $10 000), middle (>10 000 to $\leq $20 000), and high (>20 000) peak groups (Fig. 2b). In the low peak group, QTFPred achieved a mean IOU of 0.75 (SD 0.022), significantly outperforming FCN (0.64, p-value (p) = 2.58e-34; Welch’s t-test) and FCNA (0.68, p = 1.78e-16) with greater stability than FCN (SD 0.049) and FCNA (SD 0.057). This advantage persisted in middle (IOU Mean/SD: FCN 0.66/0.067, FCNA 0.71/0.073, QTFPred 0.77/0.041) and high peak groups (FCN 0.68/0.073, FCNA 0.74/0.085, QTFPred 0.79/0.050). With 45.6% of ENCODE experiments falling in the low and 69.2% in low or middle groups (Fig. 1b), QTFPred’s effectiveness with limited data provides advantages for many TFs.

Similar performance patterns appeared in A549 and GM12878 cell lines (Supplementary Fig. S5). QTFPred excelled for low (IOU Mean/SD: FCN 0.67/0.051, FCNA 0.72/0.047, QTFPred 0.78/0.031; p-values: QTFPred versus FCN 9.91e-20, versus FCNA 1.31e-9) and middle peak groups (FCN 0.67/0.044, FCNA 0.72/0.071, QTFPred 0.78/0.022; p-values: versus FCN 2.69e-75, versus FCNA 2.39e-16). For high peak groups, the performance occasionally slightly favored FCNA but not significantly, e.g. FOXA2, MAX in A549 (p = 4.50e-1, 2.86e-1) except for TCF12 in A549 (p = 3.57e-4) and YY1 in GM12878 (p = 8.93e-3).

Overall, across all cell lines and 49 TF datasets, QTFPred achieved state-of-the-art results in 45 cases (92 %), confirming its high accuracy and robustness, particularly with limited training data—crucial for TFs lacking abundant ChIP-seq peaks.

### Performance of transcription factor binding signal regression

To evaluate QTFPred’s performance in predicting TF binding signals at base resolution, we benchmarked it against BPNet and FCNsignal [34] (Supplementary Method S4) using 49 ChIP-seq datasets from three human cell lines (Supplementary Table S2): cervical carcinoma cell line from human cervix (HeLa-S3: 12 TFs), chronic myelogenous leukemia cell line from human blood (K562: 17 TFs), and B-lymphoblastoid cell line from human blood (GM12878: 20 TFs).

#### Prediction performance comparison to HeLa-S3

For HeLa-S3 experiments (Fig. 2c), we evaluated performance using Pearson correlation (score) between predicted and observed signals. We divided datasets into three peak-size groups (low, $\leq $10 000; middle, >10 000 to $\leq $20 000; high, >20 000) and examined score distributions (Fig. 2d).

In the low peak group, QTFPred (mean score = 0.70) significantly outperformed BPNet (0.36; p = 4.50e-23) and FCNsignal (0.61; p = 2.56e-3), with greater stability (SD: QTFPred 0.068 versus BPNet 0.089 versus FCNsignal 0.14). This advantage persisted in middle (score mean/SD: BPNet 0.49/0.15, FCNsignal 0.69/0.14, QTFPred 0.75/0.075) and high peak groups (BPNet 0.60/0.18, FCNsignal 0.74/0.12, QTFPred 0.78/0.081), demonstrating QTFPred’s consistent benefits across all data sizes.

#### Prediction performance comparison to the other two cell lines

We conducted identical performance comparisons for K562 and GM12878 cell lines (Supplementary Fig. S6). QTFPred consistently excelled across all peak groups: low (score mean/SD: BPNet 0.39/0.15, FCNsignal 0.58/0.17, QTFPred 0.71/0.080; p-values: versus BPNet 7.53e-60, versus FCNsignal 1.30e-14), middle (BPNet 0.47/0.16, FCNsignal 0.63/0.15, QTFPred 0.72/0.073; p-values: versus BPNet 9.61e-66, versus FCNsignal 2.04e-16), and high (BPNet 0.54/0.18, FCNsignal 0.67/0.15, QTFPred 0.74/0.071; p-values: versus BPNet 4.54e-65, versus FCNsignal 6.25e-17).

Overall, across three cell lines and 49 TF datasets, QTFPred achieved state-of-the-art results in 48 datasets (98%). These findings confirm QTFPred’s superior predictive accuracy and robustness for ChIP-seq signal prediction tasks across all peak groups.

### Validate the robust quantum-based transcription factor predictor prediction performance with a down-sampling experiment

**Figure 3:**
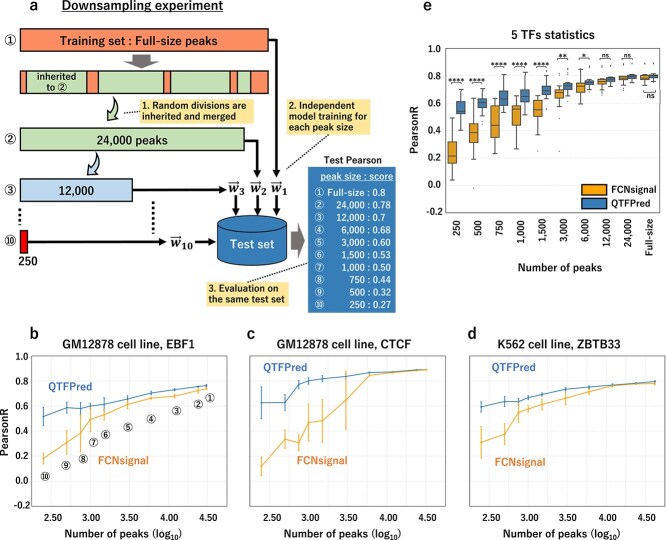
QTFPred maintains robust prediction performance with limited training data as demonstrated by down-sampling experiments, starting with (a) the experimental design for the down-sampling analysis, where training datasets of decreasing peak sizes (250, 500, 750, 1000, 1500, 3000, 6000, 12 000, 24 000, and full peaks) were created for five TFs with abundant binding regions while maintaining a nested structure to ensure consistent evaluation against a common test dataset, followed by (b–d) the comparison of the prediction performance of QTFPred and FCNsignal for EBF1 (b) in the GM12878 cell line, CTCF (c), and ZBTB33 (d) in the K562 cell line, where the horizontal axis is the size of the training dataset (log10 scale), the vertical axis is PearsonR, and the error bars indicate the standard deviation of five independent trials, and concluding with (e) the statistical comparison of the prediction performance of FCNsignal (left) and QTFPred (right) for different training data sizes, where the boxplots show the distribution of prediction performance for five TF (GM12878: EBF1 BHLHE40 CTCF, K562: ZBTB33, JUND).

Our experiments revealed QTFPred’s substantial performance improvements for TFs with limited training data. This observation suggests that QTFPred has the potential to generalize more efficiently with limited data compared with existing methods. To systematically verify this hypothesis, we evaluated prediction performance dependence on different peak sizes for the same TF (Fig. 3a and Supplementary Method S6).

We selected five TFs with sufficient binding regions (GM12878: CTCF, EBF1, BHLHE40; K562: ZBTB33, JUND) and created nested training datasets of varying sizes through random down-sampling, ensuring larger sets included smaller ones. This enabled direct comparison using a common test dataset. The training peak sizes were: 250, 500, 750, 1000, 1500, 3000, and 6000 (low), 12000 (middle), 24000 and full peaks (high).

Across all TFs in the five-trial comparison between QTFPred and FCNsignal (Fig. 3b–d), QTFPred maintained stable performance even with minimal data, while FCNsignal showed significant degradation as training data decreased. At ultra-low peak counts, the performance advantages were most pronounced. For example, with the lowest peak count (250), EBF1 showed QTFPred/FCNsignal scores of 0.52/0.18 (p = 7.90e-5), CTCF (0.63/0.12, p = 1.81e-4), ZBTB33 (0.59/0.31, p = 5.62e-3), BHLHE40 (0.56/0.35, p = 1.01e-2), and JUND (0.51/0.23, p = 8.29e-5) (Supplementary Fig. S7).

When summarizing prediction performance across all five TFs (Fig. 3e), QTFPred achieved significantly higher scores in low peak groups (QTFPred/FCNsignal score mean (SD) = 0.56 (0.080)/0.24 (0.12), 0.59 (0.064)/0.37 (0.14), 0.65 (0.079)/0.45 (0.13), 0.67 (0.082)/0.51 (0.11), 0.70 (0.074)/0.55 (0.093), 0.73 (0.062)/0.65 (0.11), 0.77 (0.056)/0.73 (0.068); p = 1.95e-14, 2.30e-8, 7.82e-8, 4.28e-7, 1.08e-7, 2.40e-3, 4.96e-2). No significant differences emerged for middle (0.79 (0.049)/0.76 (0.063); p = 1.77e-1) and high peak groups (0.81 (0.046)/0.79 (0.054), 0.81 (0.044)/0.80 (0.052); p = 3.51e-1, 3.52e-1). At the lowest count (250 peaks), QTFPred showed superior robustness while maintaining moderate predictive power (0.56 (0.080)/0.24 (0.12); p = 1.95e-14).

We also conducted the same validation using binary TFBS classification datasets (Supplementary Fig. S32). Similar to signal prediction, QTFPred demonstrated consistent superiority in low peak groups (QTFPred/FCNA IOU mean (SD) = 0.55 (0.040)/0.49 (0.004), 0.60 (0.050)/0.50 (0.031), 0.64 (0.050)/0.51 (0.046), 0.68 (0.046)/0.52 (0.058), 0.71 (0.046)/0.56 (0.075), 0.74 (0.039)/0.65 (0.077), 0.76 (0.039)/0.71 (0.069); p = 6.03e-12, 6.52e-16, 2.47e-19, 5.98e-22, 7.45e-16, 3.86e-8, 7.83e-5).

These down-sampling experiments confirm QTFPred maintains high accuracy and stable performance across different training peak sizes, particularly with limited data where other models struggle—a crucial advantage for TF binding prediction with sparse ChIP-seq peaks.

### Quantum-based transcription factor predictor enables cross-cell line transcription factor binding prediction

Building on the demonstrated robustness of QTFPred with limited training data, we further investigated whether cross-cell transfer learning—where models pre-trained on source cell lines are fine-tuned on target cell data—could enhance prediction performance beyond single-cell training. We evaluated ELK1, a low peak group TF, across three biologically diverse cell lines using cross-validation: HeLa-S3 (cervical carcinoma, 6091 peaks), K562 (chronic myelogenous leukemia, 3793 peaks), and GM12878 (B-lymphoblastoid, 7245 peaks). Each cell line served as the target while the remaining two provided source data, enabling comprehensive assessment of QTFPred’s ability to leverage information across different cellular contexts.

**Figure 4:**
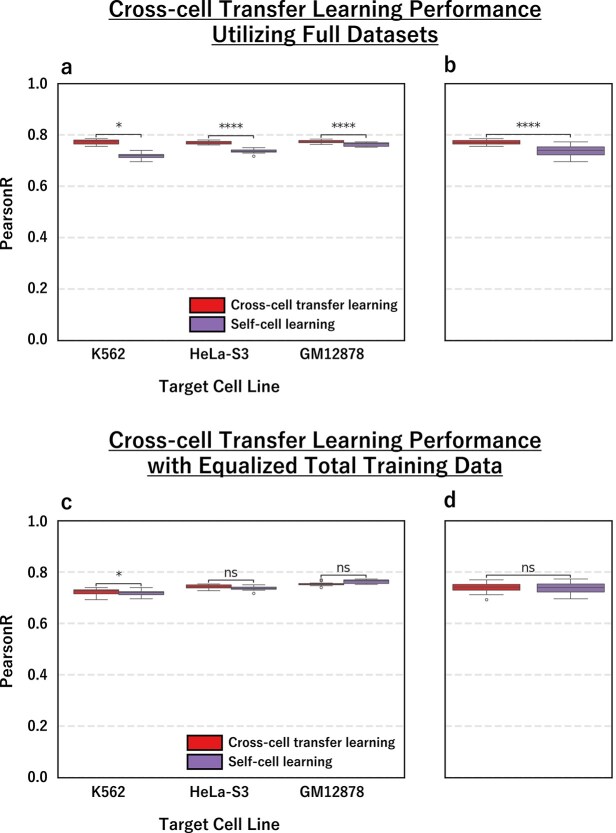
Cross-cell transfer learning performance of QTFPred for ELK1 across three cell lines, as shown by (a) and (b) the performance comparison using full datasets where models pretrained on two source cell lines are fine-tuned on target cell data (right), compared against self-cell learning (left) using only target cell data, with (a) showing Individual cell line performance across K562 (3793 peaks), HeLa-S3 (6091 peaks), and GM12878 (7245 peaks), and (b) providing the overall performance summary across all cell lines, and further illustrated by (c) and (d) the performance comparison with equalized total training data where the total amount of training data is held constant between cross-cell (right) and self-cell (left) approaches, with (c) showing individual cell line performance, and (d) providing the overall performance summary.

Cross-cell transfer learning demonstrated consistent improvements across all target cell lines (Fig. 4a and b), with QTFPred achieving significantly higher Pearson correlations compared with self-cell learning using only target cell data: K562 (cross-cell/self-cell = 0.77/0.72, p = 3.59e-8), HeLa-S3 (0.77/0.74, p = 3.87e-8), and GM12878 (0.77/0.76, p = 1.13e-2). Overall performance across all cell lines showed substantial improvement (0.77/0.74, p = 3.57e-9), confirming that QTFPred can effectively transfer knowledge between cellular contexts while maintaining its robustness with limited data.

To determine optimal data allocation strategies under resource constraints, we conducted a second experiment where total training data was held constant between cross-cell transfer learning and self-cell learning approaches (Fig. 4c and d). When experimental resources were limited, cross-cell transfer learning showed no significant advantage over self-cell learning, with comparable overall performance (0.74/0.74, p = 8.53e-1). Individual cell lines showed mixed results: slight improvements for K562 (0.72/0.72, p = 4.38e-1) and HeLa-S3 (0.74/0.74, p = 7.91e-2), but marginal decline for GM12878 (0.76/0.76, p = 2.02e-2). These results indicate that when experimental resources are limited, concentrating data collection efforts on the target cell type provides equivalent or superior performance compared to distributing resources across multiple cell types.

### Performance of computational resource

As a practical evaluation, we compared the training time, peak memory for CPU and GPU, and inference time of BPNet, FCNsignal, and QTFPred using the same computational node (Supplementary Method S7). We selected six TFs from HeLa-S3, two from each peak group (low: ELK1, ELK4; middle: REST, MAZ; high: JUND, CTCF), using a batch size of 100.

**Figure 5:**
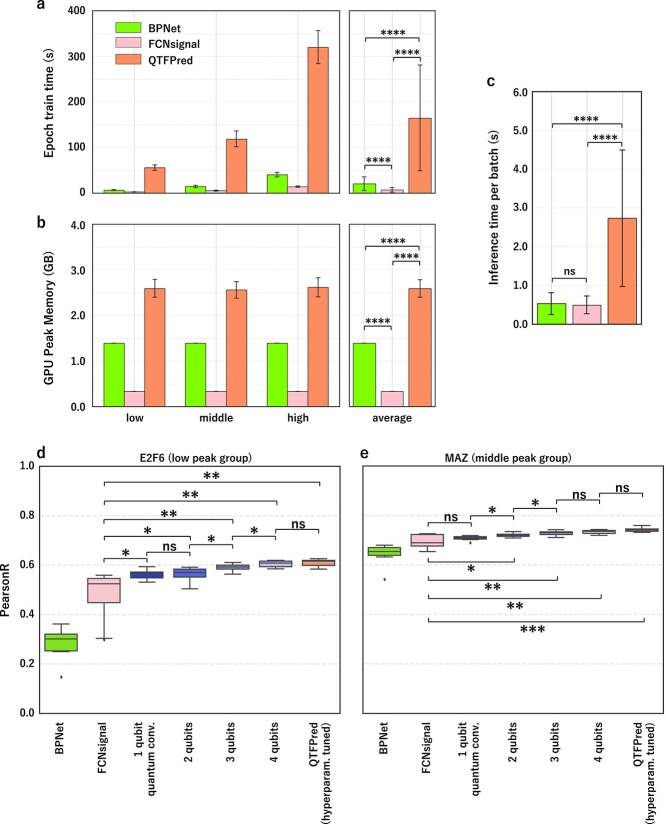
Computational resource requirements and the effect of the number of qubits on prediction performance, demonstrated by (a) the comparison of training time per epoch for BPNet (left), FCNsignal (middle), and QTFPred (right) across different peak group sizes in the HeLa-S3 cell line, box plots representing results from each 10 trials with two TFs per peak group, (b) the peak GPU memory usage during training for the three models, using BPNet (left) (c) the average inference time per batch (batch size = 100) for each model, using BPNet (left), FCNsignal (middle), and QTFPred (right), (d) the ablation study results for E2F6 (low peak group) in HeLa-S3 cell line, showing prediction performance as a function of quantum circuit complexity, and (e) the ablation study results for MAZ (middle peak group) in HeLa-S3 cell line.

For training time (Fig. 5a), FCNsignal was consistently fastest (low, 2.58/0.237 s mean/SD; middle, 5.32/0.77; high, 14.1/1.56), followed by BPNet (low, 6.61/0.985; middle, 14.7/2.59; high, 40.5/5.11) and QTFPred (low, 55.7/5.68; middle, 119/17.4; high, 320/36.1). Averaging across peak groups, FCNsignal (7.32/5.03 s) was 2.82 times faster than BPNet (20.6/15.0, p = 8.12e-9), while QTFPred (165/116) was 8.00 times slower than BPNet (p = 1.02e-13) and 22.5 times slower than FCNsignal (p = 3.74e-15). This gradient corresponds to GPU memory usage (Fig. 5b): FCNsignal (mean 0.335 GB) < BPNet (1.39 GB) < QTFPred (2.59 GB).

CPU memory usage (Supplementary Fig. S8) increased from low to high peak groups for all models. When averaged across groups, BPNet showed lowest memory usage (1.55/0.444 GB mean/SD), followed by FCNsignal (1.68/0.441 GB, p = 1.26e-1 versus BPNet) and QTFPred (1.86/0.443 GB, p = 2.47e-4 versus BPNet, p = 2.64e-2 versus FCNsignal). Notably, variation between peak groups (1.01 GB difference, 78.8% increase from low to high) exceeded variation between models (0.31 GB, 14.1–27.2%). These results suggest that CPU memory requirements are primarily determined by peak count, rather than by model architecture. Importantly, QTFPred’s CPU memory overhead versus BPNet remained consistent across all peak groups (0.31 GB), suggesting quantum circuit simulation imposes a fixed cost independent of dataset size.

For inference time (Fig. 5c and Supplementary Fig. S9), QTFPred (2.73/1.76 s) was slower than BPNet (0.529/0.283; 5.16$\times $ faster) and FCNsignal (0.491/0.227; 5.55$\times $ faster). Despite greater computational requirements, QTFPred’s superior prediction accuracy and robustness make it a practical method for TF binding prediction.

### Ablation study on the effect of the number of qubits in a quantum convolutional layer

To investigate the necessity of quantum computation in our hybrid model, we conducted an ablation study examining how qubit count affects prediction performance (Supplementary Method S8). We constructed models with varying qubit counts (1–4) using our Kernel Division Strategy, replacing FCNsignal’s first convolutional layer while preserving identical receptive field. We tested these on six TFs from HeLa-S3, selecting two from each peak group: low (E2F6, ELK1), middle (MAZ, E2F1), and high (TBP, MAX).

For low to middle peak TFs, performance improved with increasing qubit count. For E2F6 in the low peak group (Fig. 5d), FCNsignal achieved a mean/SD Pearson correlation of 0.48/0.10. Introducing just 1 qubit significantly improved performance to 0.56/0.018 (p = 2.80e-2), with continued improvements up to 4 qubits (0.61/0.013, p = 3.15e-3). For MAZ (middle peak group, Fig. 5e), performance similarly improved from FCNsignal’s 0.70/0.027 to QTFPred’s 0.74/0.009 (p = 3.08e-4). Similar patterns were observed for other low and middle peak TFs (Supplementary Fig. S10a and b).

For high peak TFs, we observed different patterns. For TBP (Supplementary Fig. S10c), models with 1–4 qubits showed no significant improvements (all p > 0.05) over FCNsignal (0.64/0.022). However, the fully optimized QTFPred significantly outperformed both FCNsignal (0.67/0.015, p = 1.47e-3) and the 4-qubit model (p = 7.98e-3), indicating that for data-rich scenarios, benefits emerge when the entire architecture is optimized. MAX showed similar results (Supplementary Fig. S10d).

This experiment confirms that the quantum computational component drives QTFPred’s superior performance, with even minimal quantum resources providing substantial advantages for TFs with limited data by enabling nonlinear transformations that extract more information-rich features than classical convolutions.

### Representation learning of known transcription factor motifs with quantum-based transcription factor predictor

To assess QTFPred’s ability to learn TF motif representations, we applied the filter activation method [34, 35] using known TF database (Supplementary Method S10). We constructed 64 position frequency matrices (PFMs) of 16 bp from the most activated sequence fragments using 64 QConv filters trained on ChIP-seq data for JUND (34 107 peaks), ELK4 (7251), and MAFF (25 548) in HeLa-S3 cell line (Supplementary Figs S11–S13).

**Figure 6:**
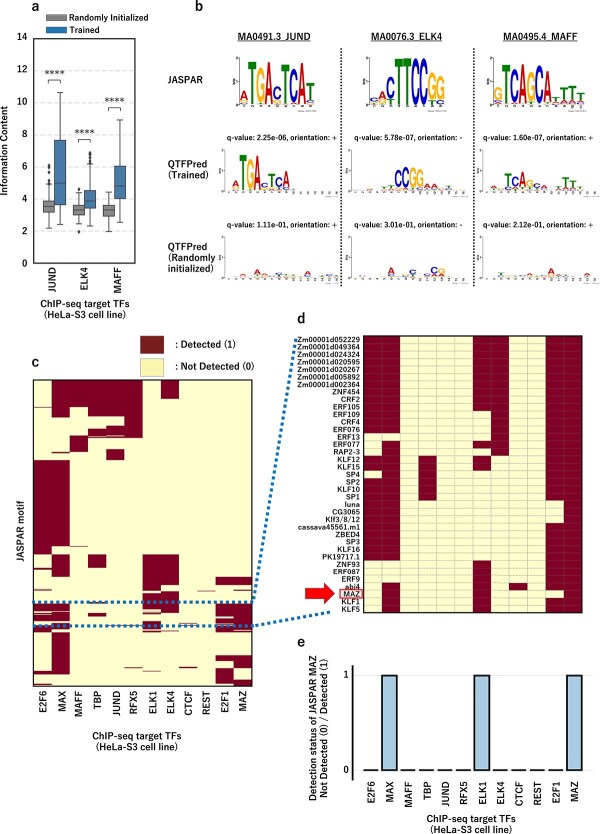
QTFPred acquires known TF motif representations through representation learning, beginning with (a) the comparison of the IC distribution of PWM (Randomly Initialized (left)) obtained from a quantum convolution filter with random weight initialization that has not been trained on the dataset, and PWM (Trained (right)) obtained from a trained filter, where the ChIP-seq target TFs are three types (JUND, ELK4, and MAFF) in the HeLa-S3 cell line, and the results of five different trials are summarized, followed by (b) the comparison of JASPAR motifs (top) and QTFPred-predicted motifs (middle, trained models for each TF in the HeLa-S3 cell line; bottom, randomly initialized models) for each TF, where each JASPAR motif is labeled with its Motif ID and TF name, and each QTFPred-predicted motif is labeled with its q-value and orientation ($+$/−), and illustrated by (c) the TF detection binary heatmap of HeLa-S3 cells based on reproducible motif matches, where the vertical-axis represents 523 JASPAR TFs detected by QTFPred in at least one ChIP-seq target TF, the horizontal-axis shows 12 ChIP-seq target TFs in HeLa-S3 cells, a JASPAR TF is labeled as detected if it matches in at least 4 out of 5 trials, and the heatmap is organized using hierarchical clustering (Ward’s method), with (d) being the enlargement of Fig. 6c, and (e) the detection status for each ChIP-seq target TFs when JASPAR motif = MAZ, extracted from Fig. 6d.

To confirm that QConv filters capture meaningful motifs, we compared information content (IC) of PFMs from trained filters against randomly initialized filters (Fig. 6a). Trained filters exhibited significantly higher IC for JUND (trained/random = 5.68/3.58, p = 1.64e-42), with similar significant improvements observed for ELK4 and MAFF (p < 1e-30), indicating more defined motif structures. This demonstrates that QTFPred learns distinct sequence patterns, while random filters produce diffuse, less informative motifs.

We compared the 64 PFMs against the JASPAR motifs [36] using TOMTOM [37] (Fig. 6b). QTFPred-derived motifs showed strong correspondence with known motifs (JUND, q = 2.25e-6; ELK4, q = 5.78e-7; MAFF, q = 1.60e-7), while randomly initialized filters showed no significant matches, demonstrating that our QConv filters effectively capture TF motif representations through QCL.

Additionally, QTFPred identified several highly informative and reproducible unannotated motifs warranting further investigation (Supplementary Result S2 and Figs S14–S16).

### Discovery of potential cis-regulatory transcription factor groups in a shared transcriptional environment

TF binding is often regulated by cooperative action of multiple TFs. Understanding this complex mechanism requires analyzing both individual binding patterns and interactions between TFs in shared transcriptional environments, e.g. specific cell types, tissue contexts, or developmental stages. The quantum convolution filters capture both primary TF binding signals (the antibody-targeted TF in ChIP-seq) and neighboring motif representations that may cooperate with the target TF by enhancing binding affinity.

To explore functional relationships between primary TFs, we developed a “TF detection binary heatmap based on reproducible motif matches” (Fig. 6c). This visualization plots 523 JASPAR TFs (vertical axis) detected by QTFPred against 12 primary TFs in the HeLa-S3 cell line (horizontal axis), with detection assigned a value of one and nondetection zero (Fig. 6d). To ensure reproducibility and minimize random matches, we defined “detection” as successful TOMTOM matching in at least four of five independent model training trials.

Each TF exhibits characteristic detection patterns. For example, the MAZ motif is detected alongside MAX and ELK1, but not with other TFs, suggesting these factors may co-occur in a cooperative cis-regulatory context in HeLa-S3 cells (Fig. 6e).

### Discovery of functionally similar transcription factor groups in a shared transcriptional environment

**Figure 7:**
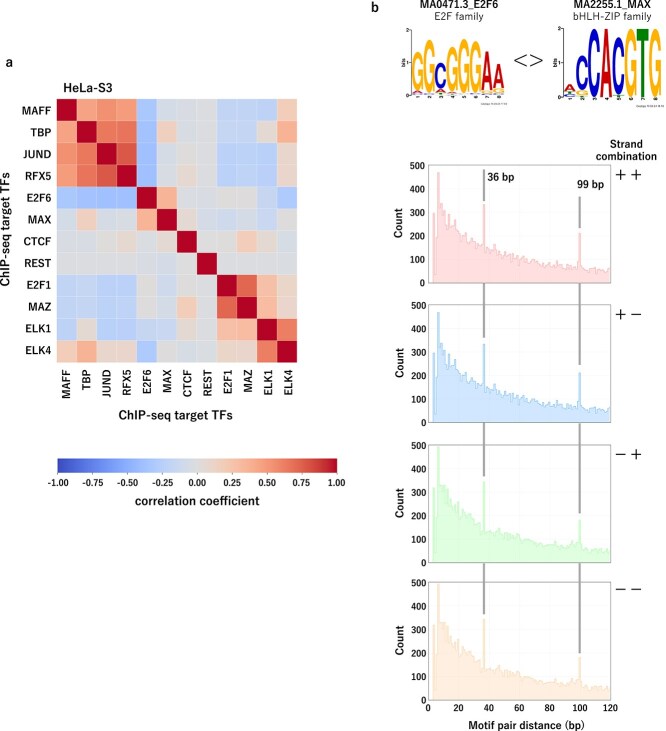
QTFPred can estimate potentially functionally similar TF pairs, as shown by (a) the correlation matrix showing the functional similarity between 12 ChIP-seq target TFs in the HeLa-S3 cell line, where each element represents the correlation coefficient of the JASPAR TF detection pattern between the corresponding ChIP-seq target TF pairs, and hierarchical clustering (Ward method) is applied to the correlation matrix, and (b) the top panel, which shows the consensus motifs of E2F6 and MAX TF registered in the JASPAR TF database, belonging to the E2F family and bHLH-ZIP family, respectively, and the bottom panel, which shows the distribution of the minimum distance between E2F6–MAX motif pairs in the ChIP-seq peak regions of MAX, where the distribution was calculated using FIMO for four different relative orientations ($++$, $+-$, $-+$, −−), and specific peaks were observed around 36 bp and 99 bp, regardless of the relative orientation.

We quantified functional similarity between primary TFs by treating their detection patterns of 523 JASPAR TFs in the HeLa-S3 cell line as binary vectors and calculating correlations between them (Fig. 7a). This analysis identified four functionally similar TF groups: (MAFF, TBP, JUND, RFX5), (E2F6, MAX), (E2F1, MAZ), and (ELK1, ELK4) (Supplementary Fig. S21).

#### Interpret the functionally similar transcription factor groups: E2F6 and MAX in the HeLa cell line

We focused on the E2F6–MAX group because these factors are functionally similar despite belonging to different motif classes—E2F6 from the E2F family and MAX binding to E-box sequences (CACGTG). Prior biochemical study confirmed this relationship, showing that MAX and E2F6 are components of the same multi-protein complex in HeLa cells, which can bind to both E2F and MYC binding sequences (E-box) [38].

#### Spatially constrained cooperative binding of E2F6 and MAX in HeLa cells

To investigate structural constraints governing E2F6–MAX binding, we analyzed minimum distances between their motifs within MAX binding peaks using FIMO [39] (Supplementary Method S13). We identified two specific distances—36 bp and 99 bp—as distinct patterns regardless of orientation (Fig. 7b and Supplementary Method S14). The shorter distance suggests direct protein interaction, while the longer indicates possible intermediary factors or nucleosome-mediated interactions. To validate these findings with independent experimental data, we analyzed an orthogonal ENCODE MAX ChIP-seq dataset from a different laboratory using the same HeLa-S3 cell line (ENCSR000EZF), which reproduced the identical distance patterns at 36 bp and 99 bp across all strand orientations (Supplementary Fig. S33), confirming the reproducibility of these spatially constrained cooperative binding relationships.

Interestingly, no specific distances were observed in E2F6 ChIP-seq binding context (Supplementary Fig. S24). This asymmetry suggests MAX binding requires a fixed spatial relationship to E2F6 motifs, while E2F6 binding is more heterogeneous. MAX likely serves as a key scaffold, dictating spatial arrangements for cooperative binding, whereas E2F6 can bind independently without fixed distancing.

These observations indicate E2F6–MAX complex formation is subject to spatial constraints driven primarily by MAX. In regulatory regions where MAX is bound, E2F6 positioning is likely dictated by MAX’s spatial preferences, while E2F6 may participate in other regulatory complexes without exhibiting the same distance-specific pattern.

## Discussion and conclusion

We developed QTFPred, achieving state-of-the-art performance across both tasks (92% and 96% of datasets for binary and signal prediction, respectively), with the most significant improvements in datasets with fewer than 10 000 peaks, which represent 45.6% of ENCODE ChIP-seq datasets. The QConv layers learn both known and unannotated motif representations, enabling the identification of functional similarities among TFs and capturing cooperative interactions.

Our evaluation revealed computational trade-offs: QTFPred was 8–22.5 times slower than existing models, with higher GPU memory requirements, but maintained consistent CPU overhead (0.3 GB) regardless of dataset size. These costs are justified by significant performance advantages for TFs with limited binding peaks when accuracy and robustness are priorities.

Our ablation study showed even a 1-qubit quantum circuit significantly improved performance for TFs with limited data, with further gains as qubits increased. This confirms the quantum computational component drives QTFPred’s advantages.

QTFPred’s modular design allows integration of quantum computational power with existing neural networks without requiring major architectural modifications. While current GPU simulations limit quantum circuit scale, future hardware, and algorithmic advances may enable larger implementations.

While QTFPred demonstrates strong performance across diverse TF binding prediction tasks, several limitations warrant consideration. First, the computational demands are substantially higher than classical models, with training times $\sim $8–22-fold slower and GPU memory requirements 1.9-fold greater, which may limit accessibility for resource-constrained research groups. Second, current quantum circuit simulation constraints restrict our implementation to 4 qubits, preventing exploration of potentially more powerful quantum architectures that could emerge with advances in quantum hardware or simulation capabilities. These limitations highlight important areas for future development, particularly as quantum computing technology continues to advance.

While QTFPred was developed as a quantum enhancement to CNN frameworks, its advantages in sparse data settings address a fundamental challenge shared across deep learning architectures, including both CNNs and large-scale models like Transformers. This positions quantum computation as a promising solution for data-limited scenarios regardless of the underlying architecture. Future developments may include quantum-enhanced Transformer models that can scale effectively even with sparse training data, and QTFPred’s implementation provides a foundational framework for such advances, demonstrating how quantum circuits can be integrated into deep learning architectures to enhance their data efficiency.

Future work will expand QTFPred’s applicability to ATAC-seq signal prediction for chromatin accessibility insights, multicell type analyses for discovering unannotated motifs and cooperative TF groups, and integration with transfer learning for extremely limited datasets, establishing it as a powerful tool for analyzing regulatory networks in complex biological systems.

## Materials and methods

### Data and preprocessing

We adopted FCNA’s data processing pipeline [18] to evaluate binary TFBS prediction performance at base resolution. We used 53 ChIP-seq datasets from three cell lines [A549 (19; four used for tuning), GM12878 (20), and MCF7 (14)] collected from the ENCODE project. We performed negative and positive labeling, using position count matrices (PCMs) obtained from the HOCOMOCO v11 motif database [40]. Each DNA sequence in the dataset is a 500 bp region centered around the binding peak.

For TF binding signal prediction at base resolution, we collected 52 ChIP-seq datasets from ENCODE [K562 (20, three used for tuning), GM12878 (20), HeLa-S3 (12)] following the FCNsignal pipeline [34]. The preprocessing steps included:

- Step 1 Obtaining the sequence position and smoothing: For positive samples, we extracted 1000 bp sequences centered on each peak position and applied random position shifts (−100 to 100 bp) to introduce noise.
- Step 2 Filtering: We excluded samples with signal values in the bottom 5% to remove low-quality data.
- Step 3 Generation of negative samples: We obtained 1000 bp sequences from positions 3000 bp upstream of each peak to serve as corresponding negative samples.
- Step 4 Normalization of signal values: We normalized signal distributions by applying the transformation ${signal{\_}transformed} = \log _{10}(1 + signal)$.

The choice of 1000 bp input sequence length for signal prediction was systematically validated through sequence length dependency analysis (Supplementary Result S4 and Fig. S27), which demonstrated optimal balance between predictive accuracy and computational efficiency at this length. The validity of our negative sampling strategy (3000 bp upstream) was confirmed through comparative evaluation (Supplementary Result S3).

The DNA sequence is represented in one-hot format: $A = [1, 0, 0, 0]$, $C = [0, 1, 0, 0]$, $G = [0, 0, 1, 0]$, $T = [0, 0, 0, 1]$.

### Evaluation of FCNA and fully convolutional network signal models

FCNA [18] and FCNsignal [34] (referred to as “reference classical models”) are U-net-like [41] encoder–decoder symmetric neural networks that predict TF binding at base resolution using DNA sequences as input.

Both models share four components: (i) an encoder with three convolutional layers that downsample input and extract sequence-specific features, (ii) a bottleneck for global context search in DNA sequences, (iii) a decoder that upsamples features through deconvolution to recover signals lost during encoding, and (iv) skip connections that transfer positional information from encoder to decoder.

The DNA sequence lengths used in our benchmarking experiments—500 bp for binary classification and 1000 bp for signal prediction—were chosen to match the evaluation settings established in the original publications of the reference classical models (FCNA [18] and FCNsignal [34]). This consistency ensures fair comparison between QTFPred and the baseline methods under identical experimental conditions.

We downloaded FCNA and FCNsignal from their respective GitHub repositories (FCNA: https://github.com/turningpoint1988/FCNA; FCNsignal: https://github.com/turningpoint1988/FCNsignal).

### Overview of quantum-based transcription factor predictor

QTFPred is a quantum-classical hybrid framework for TF binding prediction with base resolution using DNA sequences as input (Fig. 1c). QTFPred is based on Mitarai *et al.*’s QCL [30]. The QCL algorithm can search for features in higher dimensions than classical neural networks because it explores the exponentially large Hilbert space that is unique to quantum circuits [30, 42–44].

Specifically, QTFPred builds upon the neural network structure of the reference classical model, integrating quantum computational power through strategic incorporation of quantum circuits [30, 45, 46]. The encoder of the reference classical model hierarchically employs three 1D convolutional layers to extract latent features from the input DNA sequence (Supplementary Method S1) [18, 34]. Previous research has established that in hierarchical CNN models for DNA sequence analysis, the shallow layers closest to the input are particularly important for learning low-level abstract features that contribute to prediction, such as motifs [18, 34, 47, 48]. Therefore, we strategically selected the first convolution layer (Conv1) of the reference classical model for quantum enhancement, as it directly processes the input DNA sequence. Specifically, we replaced the classical Conv1 layer with a QConv layer to form the first layer of our model, which we refer to as QConv1 (Fig. 1d and Supplementary Fig. S1).

In the QConv1 layer, the input sequence undergoes processing through the qconv operation, which is a 1D quantum-based convolution operation utilizing quantum circuits (Supplementary Fig. S2). The resulting feature map is then passed as input to the subsequent classical neural network layers, beginning with Conv2.

The subsequent layers following QConv1 maintain the same structure as the reference classical model and perform hierarchical feature extraction (Supplementary Figs S3 and S4). The decoder part upsamples the feature values to the original input size and directly outputs the predicted values at each base position—either binding probability (for TFBS prediction) or binding signal (for signal prediction), depending on the task. Following the approach of the reference classical model, QTFPred is trained through gradient descent based on a cost function defined by the difference between the output prediction and the ground truth label.

For binary TFBS prediction, we used FCNA as the reference model for quantum enhancement, while for signal prediction, FCNsignal served as the reference model. Training and evaluation were conducted separately for each task using GPU-based quantum circuit simulation.

For detailed algorithmic procedures and implementation, see Supplementary Methods S2 and S3.

### Architecture of quantum convolutional layer

The QConv1 layer implements QCL through three sequential operations—encoding, unitary transformation, and measurement—to extract DNA sequence features in an exponentially enhanced feature space.

QConv1 employs angle encoding to transform one-hot encoded DNA sequences into quantum states. Each nucleotide value $x_{i} \in \{0,1\}$ from the one-hot representation is mapped to a quantum state through Y-axis rotation: 


(1) \begin{align*}& |x_{i}\rangle = R_{Y}(\pi x_{i})|0\rangle = \cos\left(\frac{\pi x_{i}}{2}\right)|0\rangle + \sin\left(\frac{\pi x_{i}}{2}\right)|1\rangle,\end{align*}

where $R_{Y}(\theta )$ denotes the rotation gate. This encoding maps each binary value to a 2D complex vector (qubit state). For our 4-qubit system processing 4 bp windows, the input quantum state becomes: 


(2) \begin{align*}& |\psi_{\text{in}}\rangle = \bigotimes_{i=1}^{4} |x_{i}\rangle \in \mathbb{C}^{2^{4}}.\end{align*}

This encoding performs the transformation: 


(3) \begin{align*}& f_{\text{encode}}: \mathbb{R}^{4} \rightarrow \mathbb{C}^{16},\end{align*}

which exploits the tensor product structure to automatically generate all multiplicative combinations of input features [30]. The resulting 16-D quantum state encodes these products as complex amplitudes without explicit computation. While classical machine learning must resort to kernel methods to implicitly access high-dimensional feature spaces [49], quantum circuits operate directly in this exponentially scaled space where each of the $2^{n}$ dimensions serves as a computational resource [30]. This direct operation in exponentially large spaces—as opposed to implicit kernel mappings—makes classical simulation of quantum circuits computationally intractable as qubit count increases [30]. This exponential scaling of representational capacity enables QCL to efficiently model the complex sequence–function relationships that arise in TFBS prediction.

Following encoding, a PQC applies unitary transformations $U(\theta )$ to the input state: 


(4) \begin{align*}& |\psi_{\text{out}}\rangle = U(\theta)|\psi_{\text{in}}\rangle,\end{align*}

where $\theta $ represents trainable parameters optimized through gradient descent. For our 4-qubit system, $U(\theta )$ is a $2^{4} \times 2^{4}$ unitary matrix implementing the transformation $\mathbb{C}^{16} \rightarrow \mathbb{C}^{16}$.

Our quantum circuit design incorporates entangling gates to generate quantum entanglement—correlated quantum states where measurement outcomes of one qubit depend on others in ways that cannot be reproduced by classical systems [26]. In the context of DNA sequence analysis, entanglement enables the circuit to capture nonlocal dependencies between nucleotides. While classical convolutions process features independently at each position before combining them linearly, entangled quantum states maintain inherent correlations throughout processing. This property allows QConv1 to detect complex, nonadditive interactions between multiple nucleotide positions that may be crucial for TF binding specificity—patterns that conventional CNNs might miss due to their local, separable feature representations.

The measurement stage decodes quantum states back to classical values through expectation value calculations. For each qubit $i$, we compute: 


(5) \begin{align*}& f_{i}(\theta) = \langle\psi_{\text{out}}|\sigma_{z}^{(i)}|\psi_{\text{out}}\rangle = \langle\psi_{\text{in}}|U^{\dagger}(\theta)\sigma_{z}^{(i)}U(\theta)|\psi_{\text{in}}\rangle,\end{align*}

where $\sigma _{z}^{(i)}$ is the Pauli-Z operator acting on the $i$th qubit. These real-valued features serve as inputs to subsequent neural network layers.

#### Kernel division strategy for practical implementation

QConv1 was designed with a target kernel size of 16 bp based on two key considerations. First, this size is sufficient to capture most TF binding motifs, as 96.5% of known motifs in JASPAR2024 are 16 bp or shorter (Supplementary Fig. S25). Second, the reference classical models (FCNsignal and FCNA) employ a 16 bp convolutional kernel in their first layer. By matching this kernel size while replacing classical convolution with quantum processing, we enable direct performance comparison to isolate the pure effect of quantum enhancement in our benchmarking experiments.

However, simulating a 16-qubit quantum circuit is computationally prohibitive due to the exponential scaling of quantum state dimensions. To address this challenge while maintaining the target receptive field, we developed the Kernel Division Strategy that partitions the convolution operation: 


(6) \begin{align*}& k_{\text{target}} = k_{\text{quantum}} + k_{\text{classical}} - 1 = 4 + 13 - 1 = 16,\end{align*}

where we adopt $k_{\text{quantum}} = 4$ (enabling efficient computation in a $2^{4} = 16$D space) and $k_{\text{classical}} = 13$ for complementary classical convolution.

Beyond computational efficiency, this hybrid architecture offers several advantages for TFBS prediction. The quantum component with its exponential feature space excels at capturing complex, short-range interactions within the 4 bp window—the scale at which critical base-specific binding determinants operate. The subsequent classical convolution then aggregates these quantum-extracted features across a broader 16 bp context, enabling detection of extended motif patterns while preserving the rich feature representations from quantum processing. This division mirrors the hierarchical nature of TF-DNA recognition, where specific base contacts combine with broader sequence context to determine binding affinity.

### Model training

The model was trained using either NVIDIA H100 or A100 GPUs. AdamW [50] was used as the optimizer, with updates applied to all parameters including variational parameters of the PQC. We used the Xavier method [51] to initialize all QTFPred weights, including those within the PQC. The maximum training epochs were set to 40, with early stopping (patience=6).

We used random data partitioning to evaluate each ChIP-seq dataset [34]. For binary classification, we allocated 80% of the data for training and 20% for testing. Unlike the original FCN/FCNA implementation [18], which set the first convolution layer’s kernel size to match each TF’s motif length from HOCOMOCO PCMs, we adopted a fixed kernel size of 16 for all TF datasets to avoid providing prior motif information. The binary classification task used single-precision (32-bit floating-point) computations.

For signal prediction, we maintained the same 80%/20% training/testing split, with both partitions including negative samples. Signal prediction was executed using double-precision (64-bit floating-point) computations.

We used hard negative mining loss [52, 53] for binary classification and root mean squared error (RMSE) for signal prediction. All benchmark comparisons used identical data partitions controlled by the same random seed. We conducted 10 independent trials for each experiment to ensure robust performance evaluation.

Hyperparameter tuning was performed for all models (Supplementary Method S5).

### Motif analysis

#### Motif calculation algorithm

We extracted motif representations learned by the QConv1 layer (trained on TF signal datasets) by converting QConv filters to PFMs [34, 35] (Supplementary Method S10). The process involves several steps:

First, we processed each DNA sequence in the test dataset through the pretrained QTFPred model to identify 100 bp sub-regions with the highest predicted binding signals. For these extracted regions, we then calculated activation scores from each of the 64 filters in the QConv1 layer (comprising both quantum convolution and complementary classical convolution). Using a sliding window approach, we identified the 16 bp subsequences that produced the highest activation score for each filter. These high-scoring sequences were collected across all test data and aligned by filter, allowing us to calculate the frequency of each nucleotide (A, C, G, T) at each position, thereby constructing 64 distinct PFMs of 16 bp length.

We compared these PFMs against the JASPAR 2024 CORE nonredundant database [36], a comprehensive collection of experimentally validated TF binding sites in eukaryotes, using the TOMTOM tool [37] with a statistical significance threshold of q-value < 0.1, consistent with previous studies [35].

To quantify the information richness of each motif, we calculated the IC of the constructed PFMs using: 


(7) \begin{align*}& \text{IC} = -\sum_{\substack{i,j}} b_{j} \log_{2}(b_{j}) + \sum_{\substack{i,j}} m_{ij} \log_{2}(m_{ij}),\end{align*}

where $m$ is the 16 $\times $ 4 PFM and $b$ is a 4-length vector representing the background nucleotide frequencies, assumed to be uniformly distributed.

#### Estimation of transcription factor pairs with functional similarity

The “TF detection binary heatmap based on reproducible motif matches” is a matrix that shows reproducible patterns of known TF binding motifs that QTFPred has learned by training on binding signals of each primary TF. Primary TFs (the antibody-targeted TFs in ChIP-seq) are placed on the horizontal axis and JASPAR TFs on the vertical axis, with detection represented as one and nondetection as zero. A JASPAR TF is considered detected when TOMTOM matching is successful with at least one of 64 PFMs in $\geq $ 4/5 independent trials, ensuring only highly reproducible and robust detections are counted while excluding accidental matches.

To quantify functional similarity between primary TFs, we calculated Pearson correlation coefficients between their JASPAR TF detection vectors, generating a correlation matrix. Hierarchical clustering of this matrix identified potential functionally related primary TF pairs and groups.

**Key Points**

- Quantum-based transcription factor predictor (QTFPred) is a novel quantum-classical hybrid deep learning model that significantly improves transcription factor (TF) binding prediction at base resolution, particularly excelling with limited training data. This addresses a common challenge in genomics where many TFs have sparse ChIP-seq peaks.
- QTFPred demonstrates the ability to learn and represent both known and novel TF motifs, providing insights into cooperative binding mechanisms between TFs within specific cellular contexts. For example, it uncovered a spatially constrained cooperative binding relationship between E2F6 and MAX in HeLa cells.
- While QTFPred has higher computational demands for training and inference compared with classical models, its superior accuracy and robustness—especially with limited data—justify these trade-offs for critical TF binding prediction tasks. An ablation study confirmed that even minimal quantum resources (e.g. 1-qubit) contribute substantially to performance gains.

## Supplementary Material

Supplementary_Material_bbaf604

## Data Availability

The data supporting the findings of this study are available within the paper and its Supplementary Information. The source code utilized in this study are publicly available at https://nagasakilab.csml.org/en/qtfpred. All ENCODE ChIP-seq datasets used in this study are publicly available and were obtained from the ENCODE Project Consortium (https://www.encodeproject.org/). Specific ENCODE experiment IDs for all datasets used are provided in Supplementary Tables S1 and S2. For binary classification tasks, ChIP-seq data were downloaded from the FCNA repository (https://github.com/turningpoint1988/FCNA). For signal prediction tasks, datasets were obtained directly from ENCODE using the experiment IDs listed in Supplementary Table S2. The JASPAR 2024 CORE nonredundant TF binding site database used for motif analysis is publicly available at http://jaspar.genereg.net/. The HOCOMOCO v11 motif database used for data preprocessing is available at https://hocomoco11.autosome.org/.
