# Supplementary material for: QTFPred: robust high-performance quantum machine learning modeling that predicts main and cooperative transcription factor bindings with base resolution
Source: Brief Bioinform. 2025 Nov 26;26(6):bbaf604. doi: 10.1093/bib/bbaf604 (PMC12648403; doi:10.1093/bib/bbaf604)
Supplement: Supplementary_Material_bbaf604 [file supplementary_material_bbaf604.pdf]

Supplementary Material

Supplementary Figures and Tables

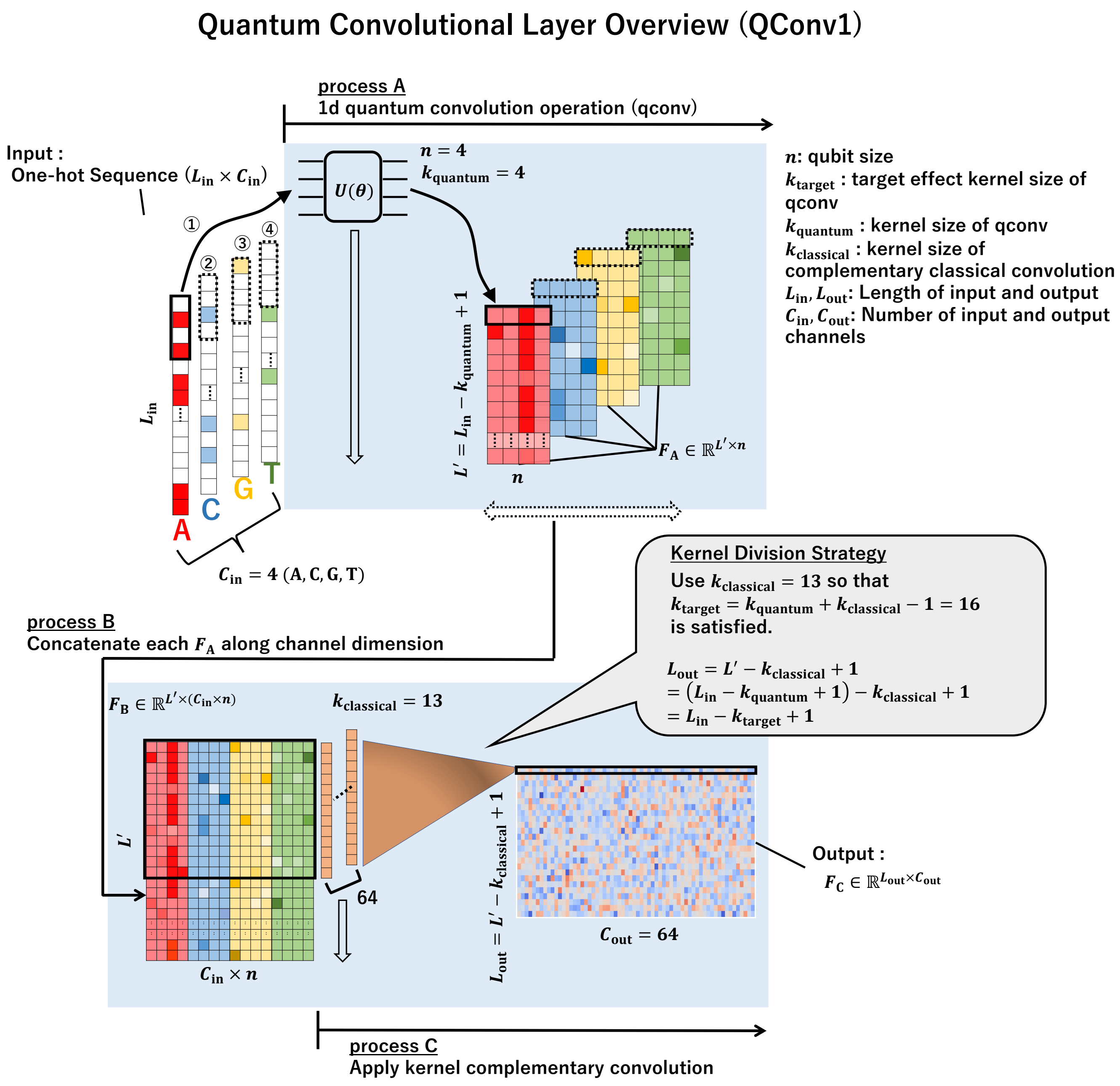

Supplementary Fig. 1: Detailed Quantum Convolutional Layer Architecture
Process A applies quantum convolution to each input channel, Process B concatenates these results, and Process C performs classical convolution for receptive field complementation.

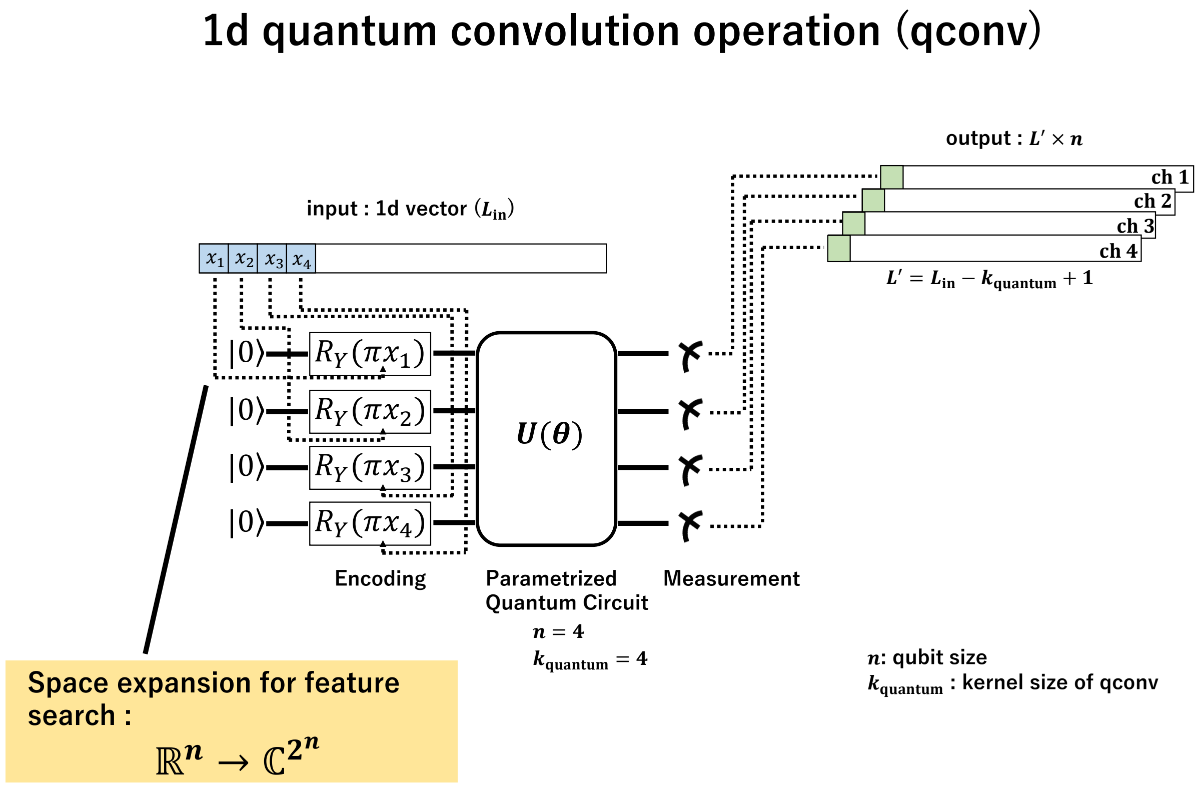

Supplementary Fig. 2: qconv: 1D Quantum Convolution Operation
The qconv operation executes quantum convolution in the QConv layer, performing the transformation $f_{qconv} : \mathbb{R}^{L_{\text{in}}} \rightarrow \mathbb{R}^{L' \times n}$, where $L' = L_{\text{in}} - k_{\text{quantum}} + 1$. Classical information is angle-encoded into a quantum state, processed through a parameterized quantum circuit (PQC), and decoded via expectation value measurements. Each qubit measurement produces a separate output channel, enabling feature extraction in an exponentially enhanced quantum feature space.

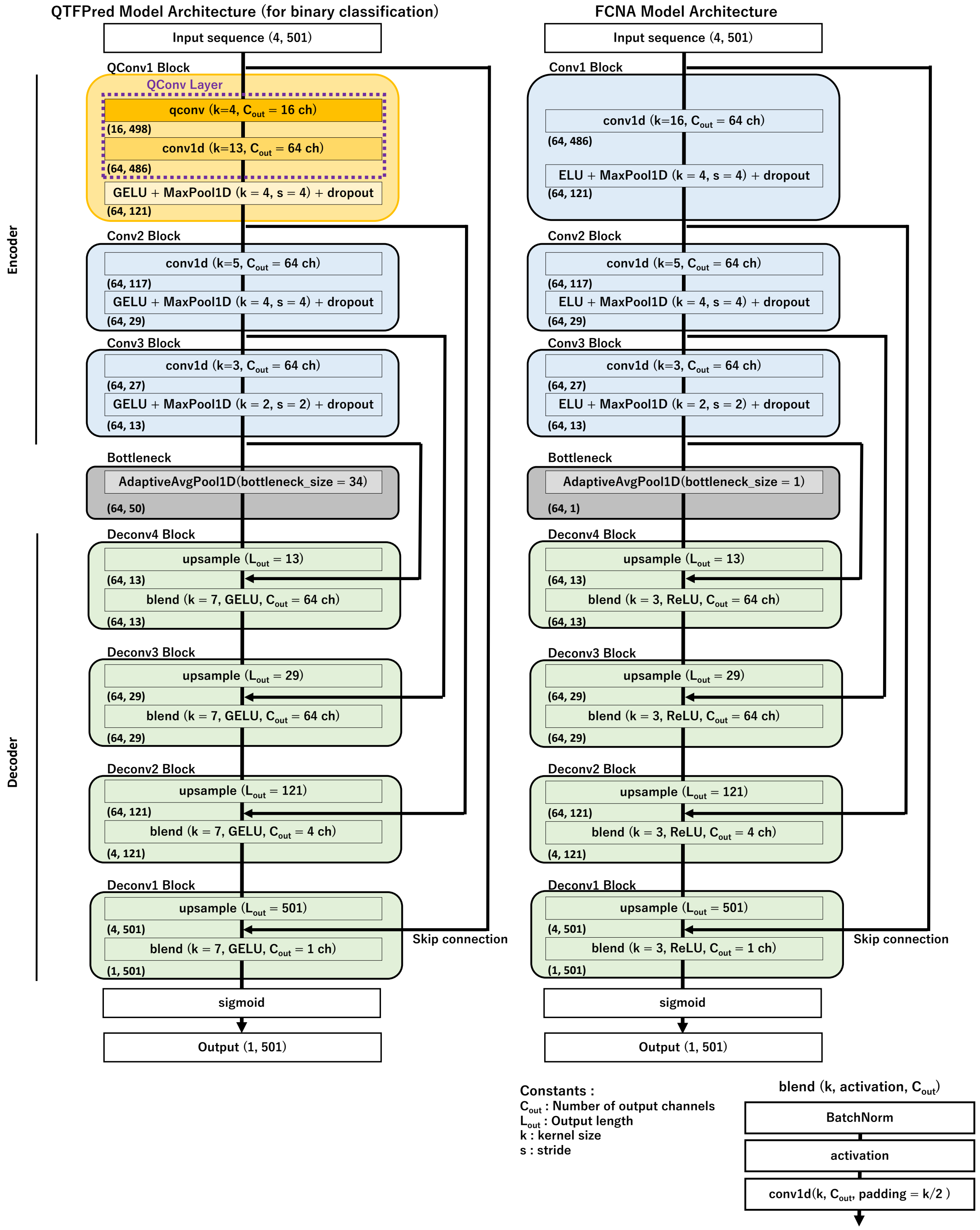

Supplementary Fig. 3: QTFPred Model Architecture for binary classification and comparison to FCNA The encoder begins with a quantum convolution layer (QConv1) with 4 qubits, followed by a classical convolution that produces 64 channels. The final output represents binding probabilities at each base position.

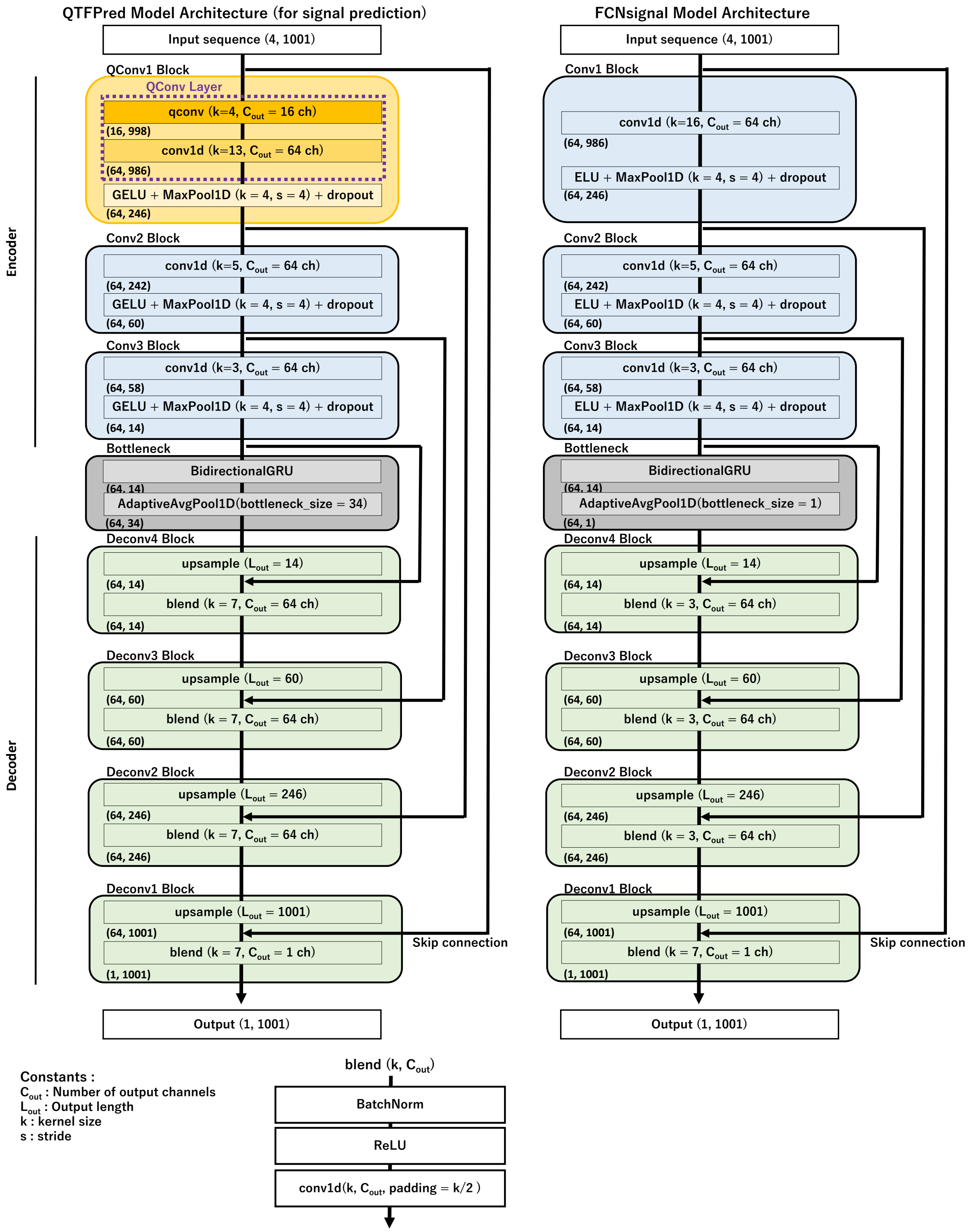

Supplementary Fig. 4: QTFPred Model Architecture for signal prediction and comparison to FCNsignal The encoder begins with a quantum convolution layer (QConv1) with 4 qubits, followed by a classical convolution that produces 64 channels. The final output represents continuous binding signals across the entire sequence.

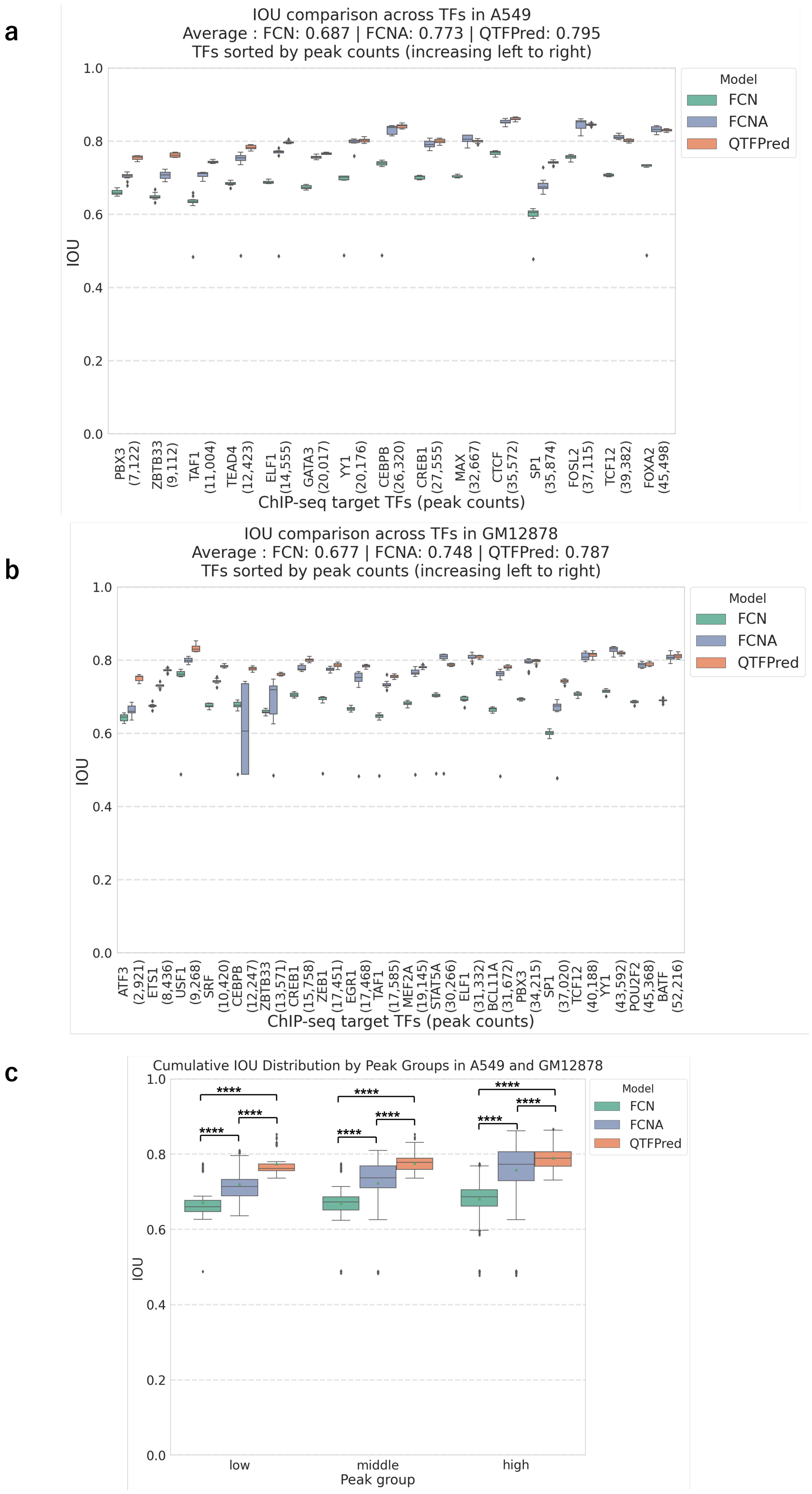

Supplementary Fig. 5: Binary TFBS prediction performance across multiple cell lines. (a) Comparison of IOU scores for FCN, FCNA, and QTFPred across 15 TFs in the A549 cell line, ordered by increasing peak count. Box plots represent results from 10 independent trials. (b) Comparison of IOU scores for 20 TFs in the GM12878 cell line, ordered by increasing peak count. (c) Cumulative IOU score distributions by peak groups (low (≤10,000), middle (>10,000 to ≤20,000), and high (>20,000)) for A549 and GM12878 cell lines combined.

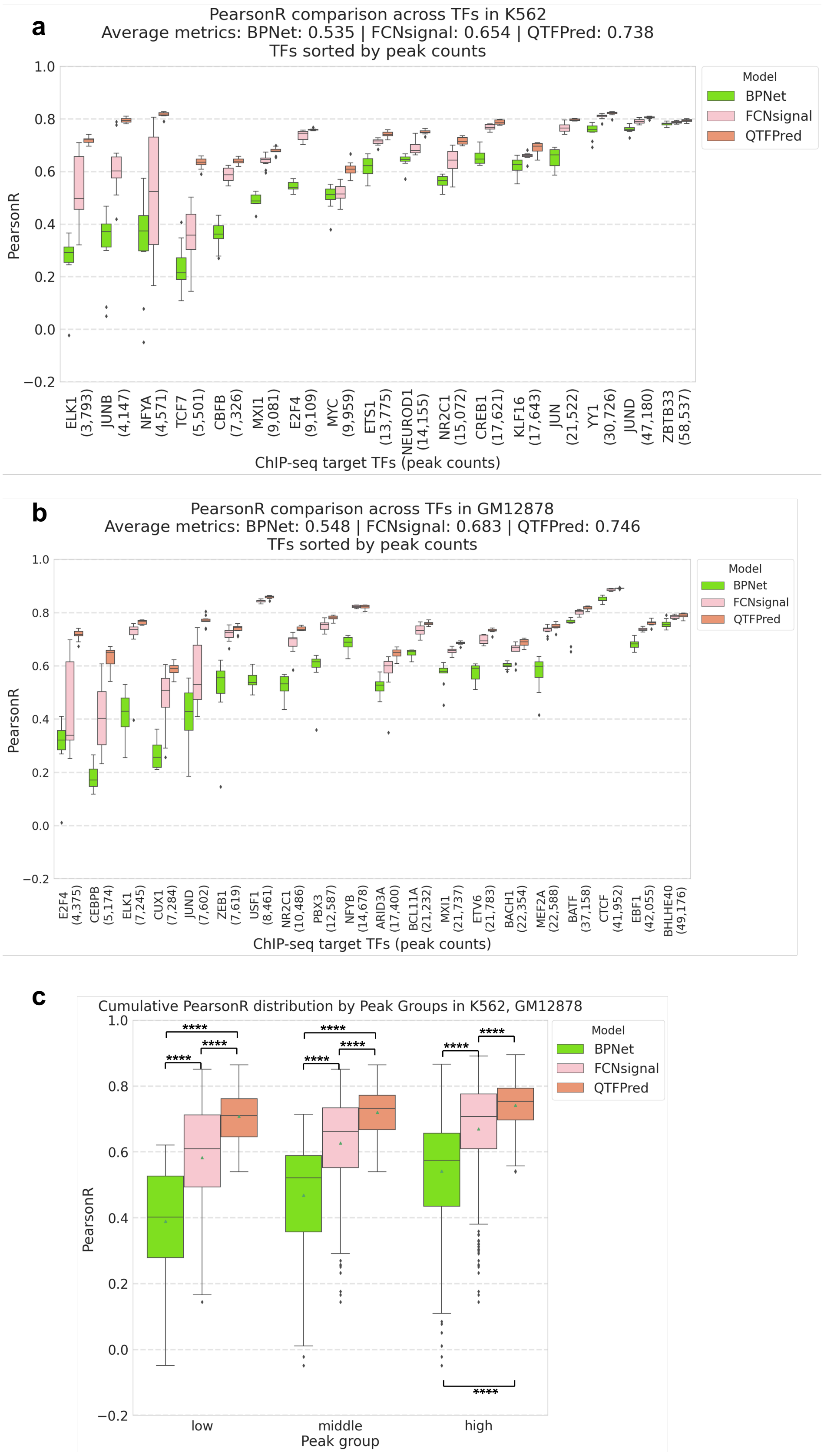

Supplementary Fig. 6: Signal prediction performance across multiple cell lines. (a) Comparison of Pearson correlation scores for BPNet, FCNsignal, and QTFPred across 17 TFs in the K562 cell line, ordered by increasing peak count. The boxplots are based on 10 independent trials. (b) Comparison of Pearson correlation scores for 20 TFs in the GM12878 cell line, ordered by increasing peak count. (c) Cumulative Pearson correlation distributions by peak groups for K562 and GM12878 cell lines combined.

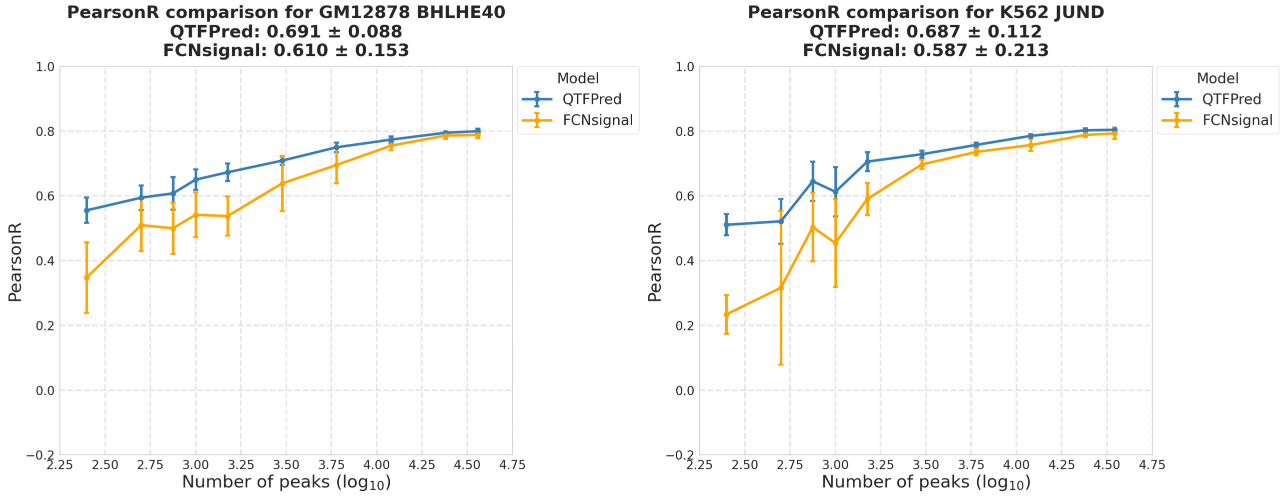

Supplementary Fig. 7: Results of downsampling experiments on the training data set. Error bars indicate the standard deviation of five independent trials. (left) BHLHE40 TF in the GM12878 cell line. (right) JUND TF in the K562 cell line.

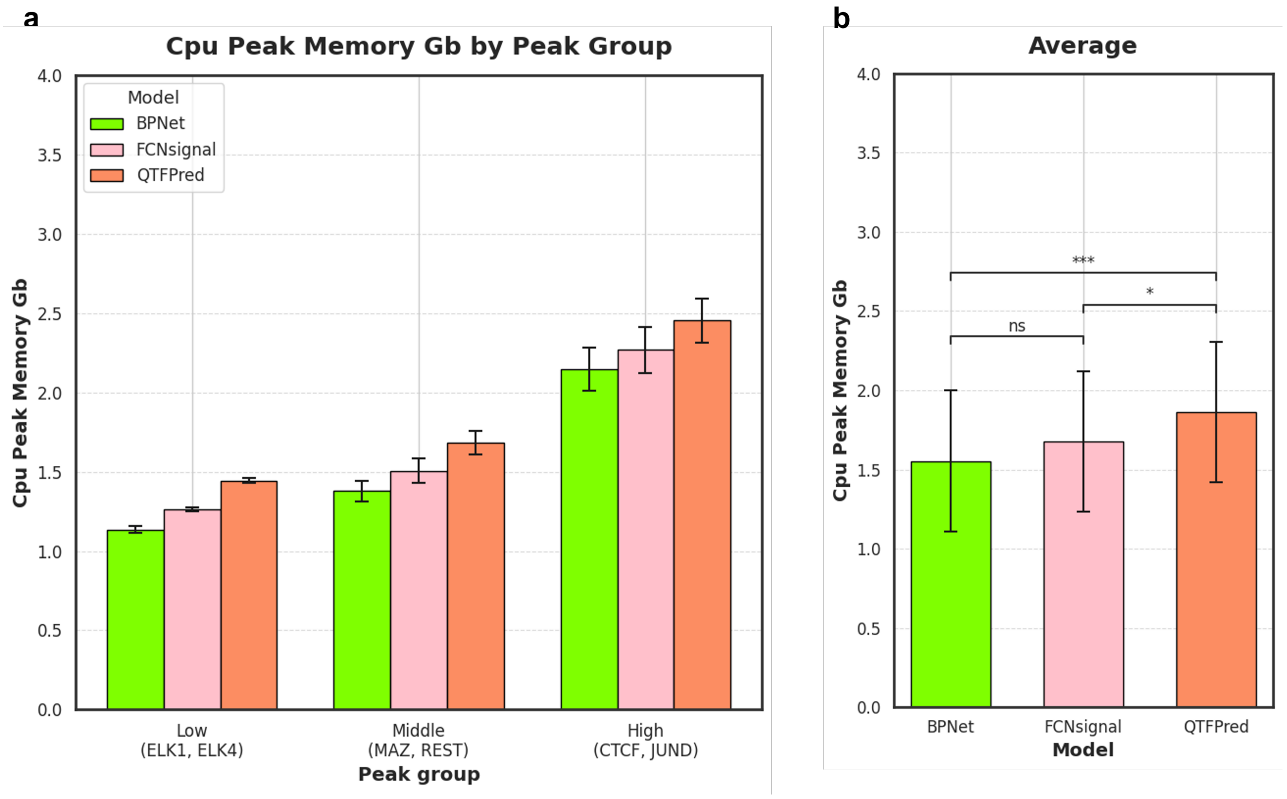

Supplementary Fig. 8: CPU memory requirements for TF binding prediction models across different peak categories. (a) Comparison of CPU peak memory usage for BPNet, FCNsignal, and QTFPred across three ChIP-seq peak groups in the HeLa-S3 cell line. All models show a consistent increase in memory consumption from low to high peak groups, with QTFPred consistently requiring slightly more memory than BPNet and FCNsignal. (b) Average CPU peak memory usage across all peak groups for the three models.

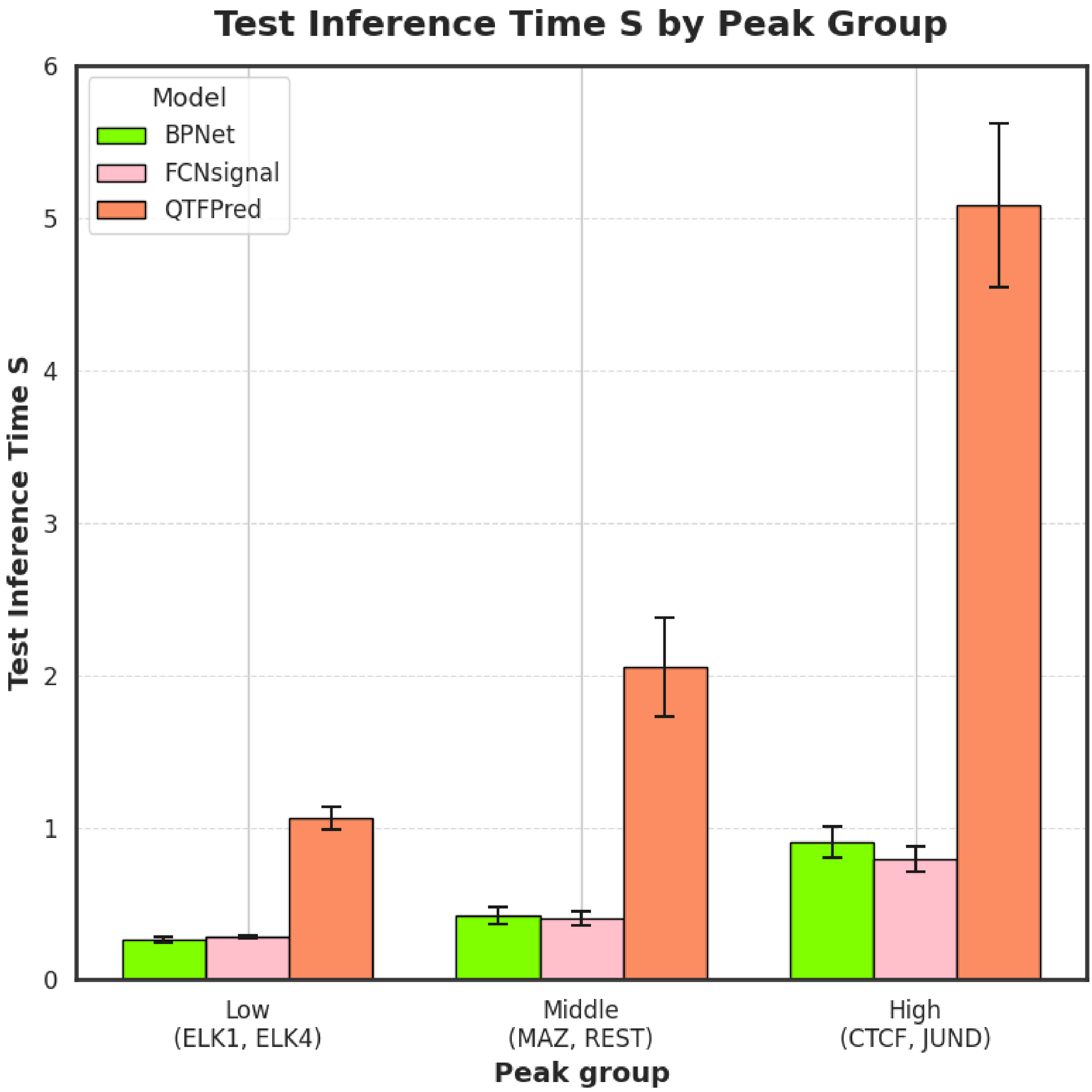

Supplementary Fig. 9: inference time comparison for BPNet, FCNsignal, and QTFPred across different peak groups in the HeLa-S3 cell line.

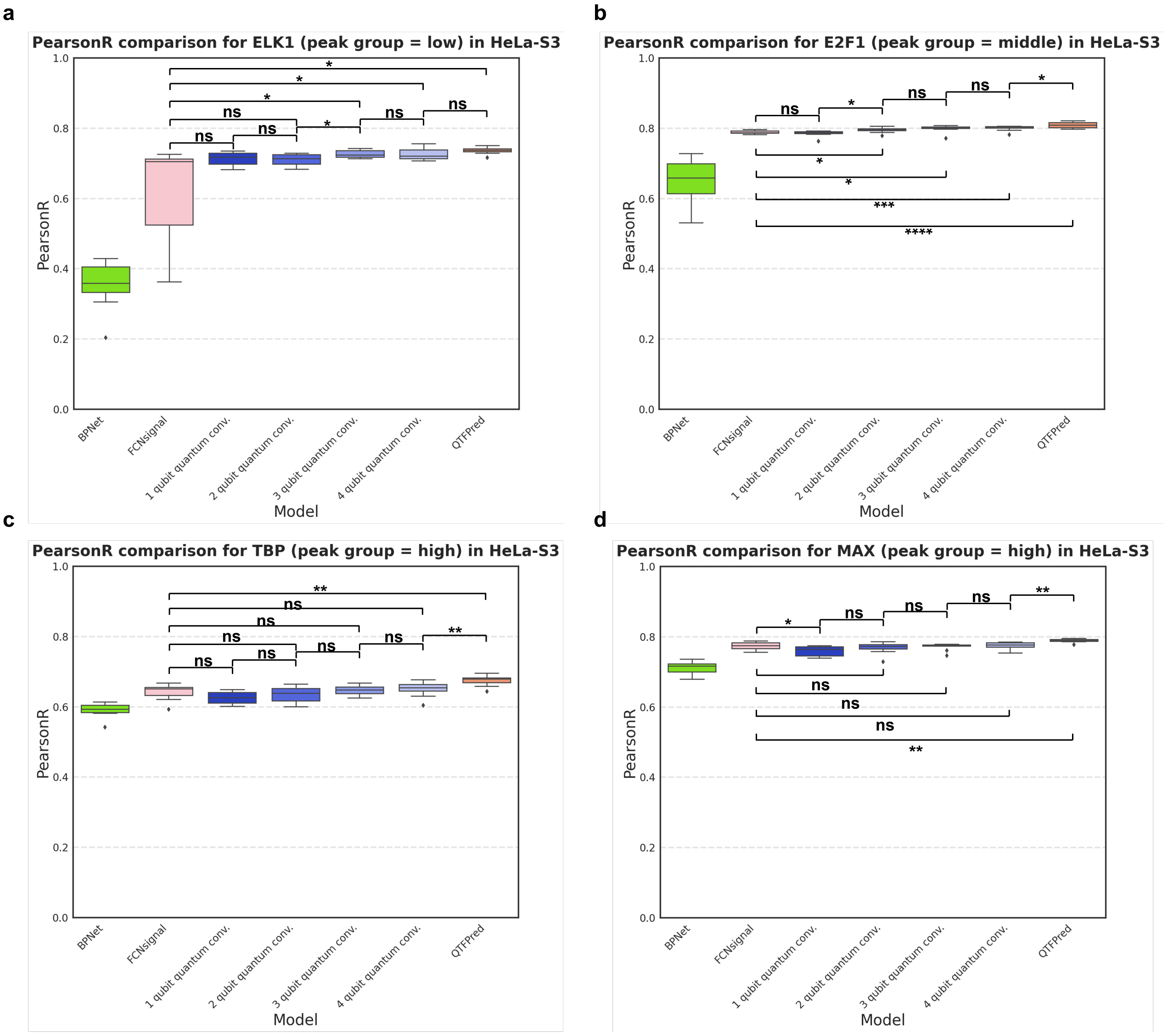

Supplementary Fig. 10: Effect of the number of qubits on prediction performance across different TF datasets. Ablation study comparing prediction performance (Pearson correlation) of models with increasing quantum resources across four representative transcription factors (a) ELK1 (low peak group), (b) E2F1 (middle peak group), (c) TBP (high peak group), (d) MAX (high peak group) in the HeLa-S3 cell line.

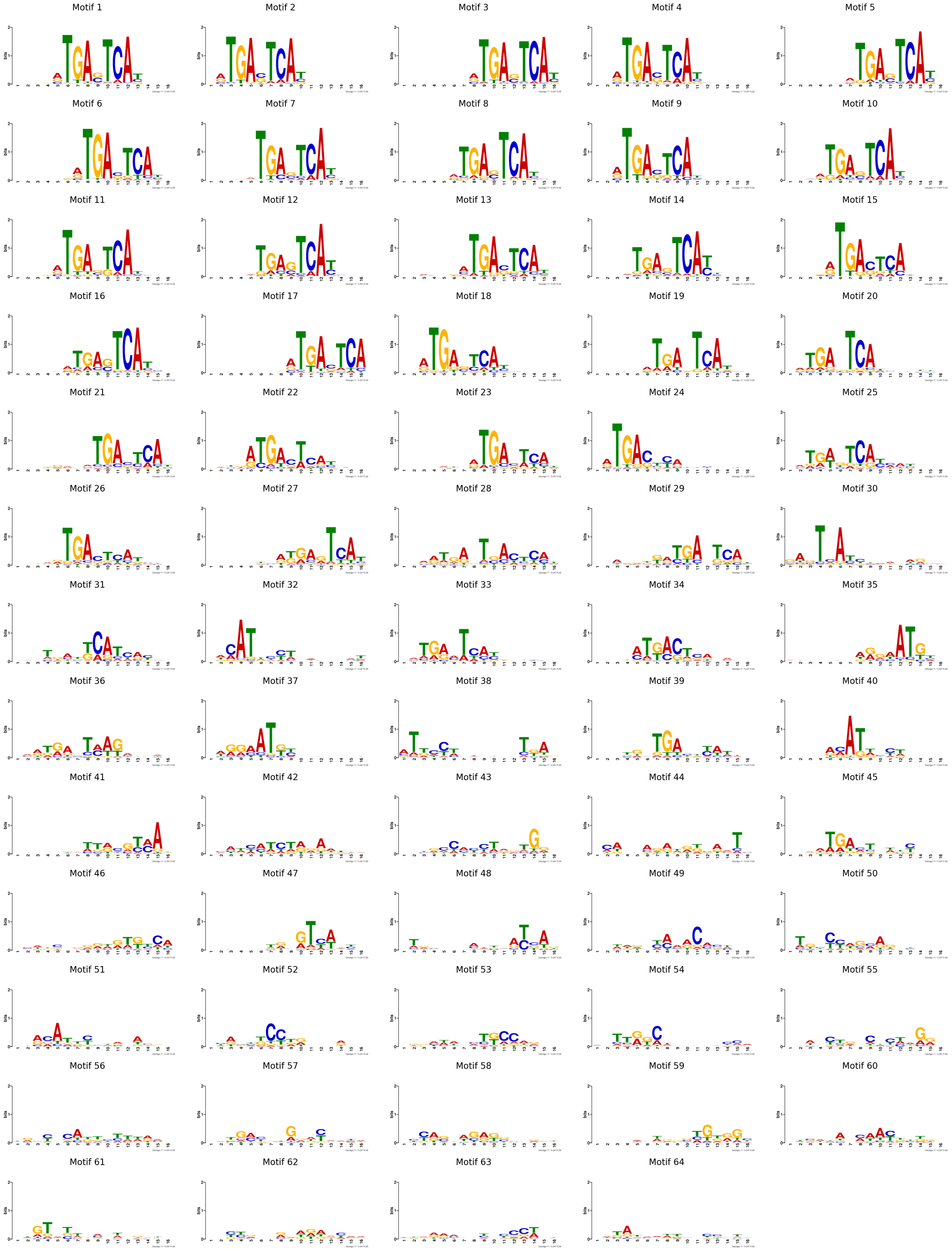

Supplementary Fig. 11: 64 motif representations derived from QTFPred trained on JUND ChIP-seq data from HeLa-S3 cell line. Motifs are arranged in descending order of information content.

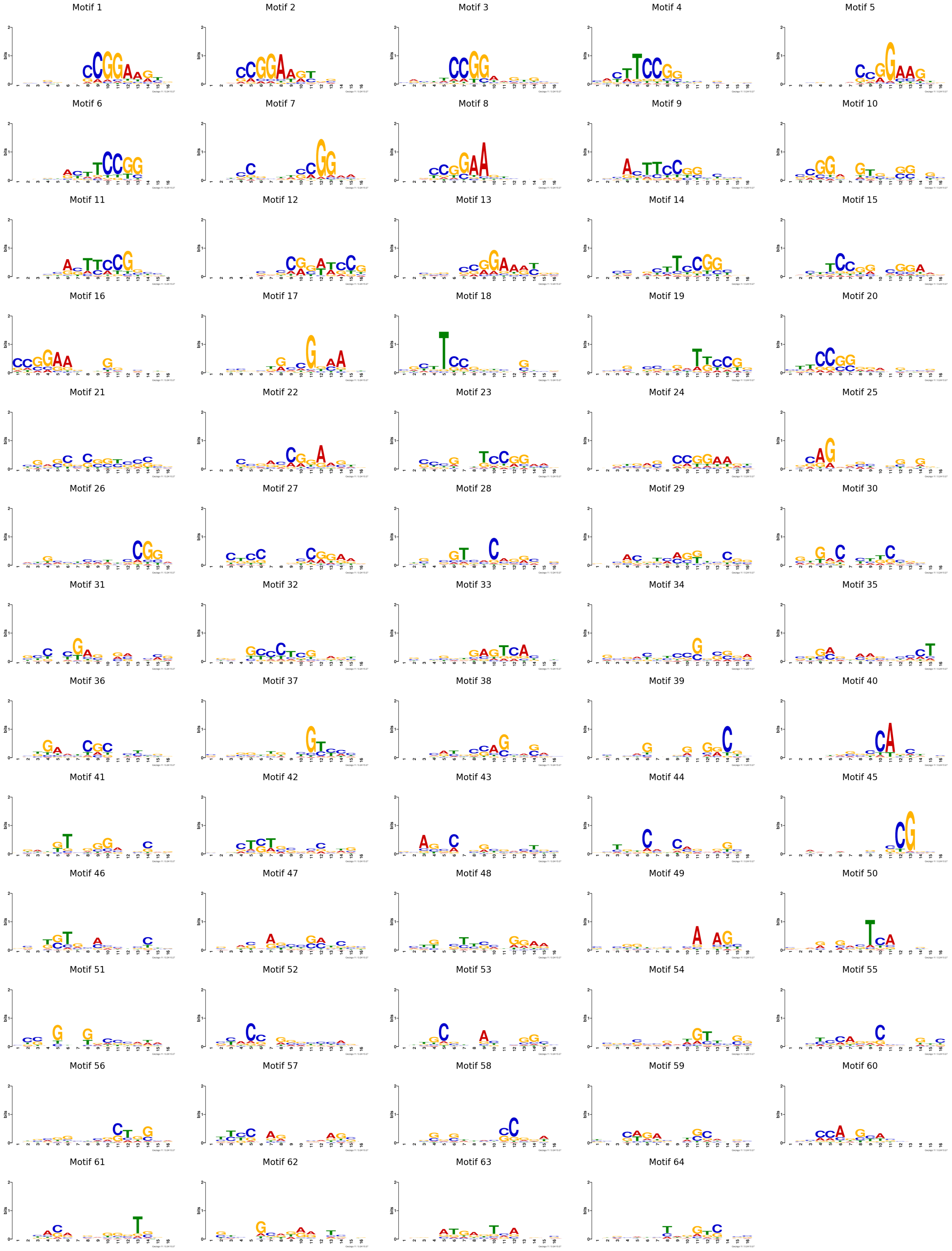

Supplementary Fig. 12: 64 motif representations derived from QTFPred trained on ELK4 ChIP-seq data from HeLa-S3 cell line. Motifs are arranged in descending order of information content.

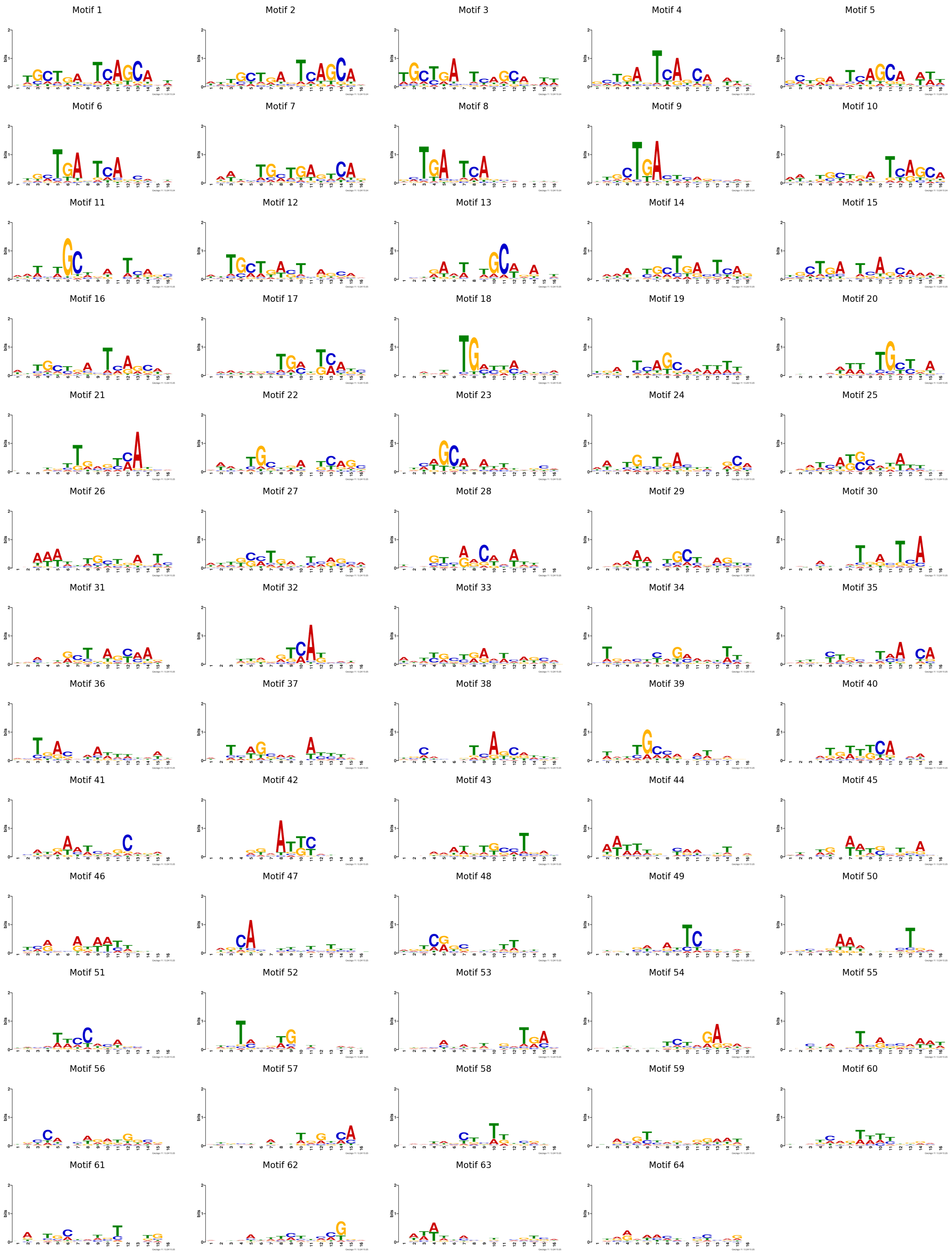

Supplementary Fig. 13: 64 motif representations derived from QTFPred trained on MAFF ChIP-seq data from HeLa-S3 cell line. Motifs are arranged in descending order of information content.

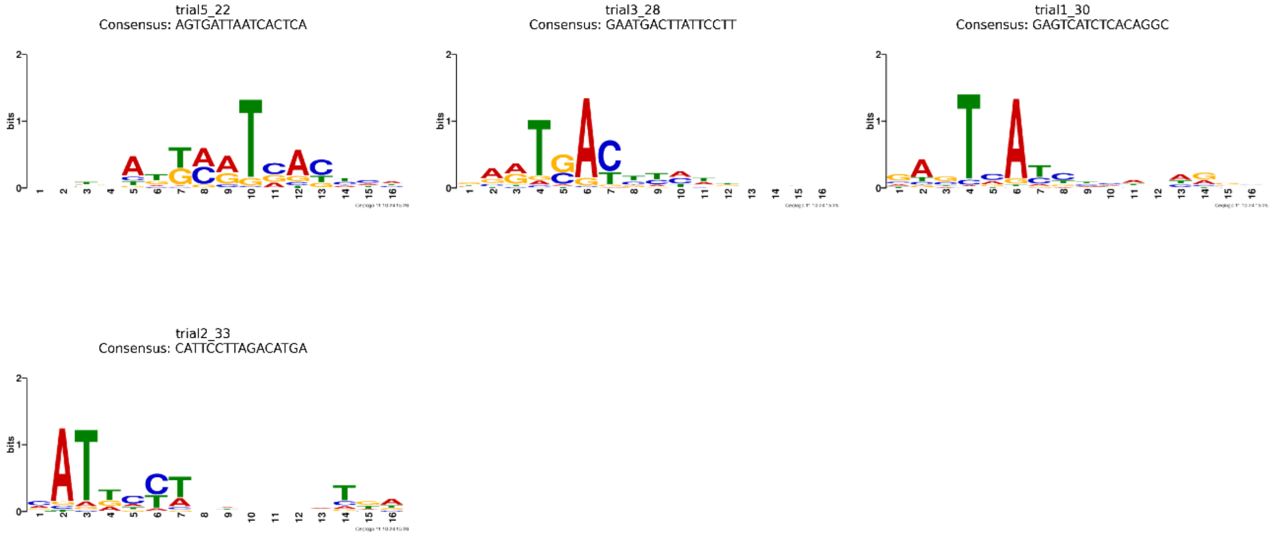

Supplementary Fig. 14: Reproducible unannotated motifs extracted from models trained on JUND signals in HeLa-S3 cell line. Motifs are arranged in descending order of information content, with each panel showing the trial number (1-5), motif ID, and consensus sequence.

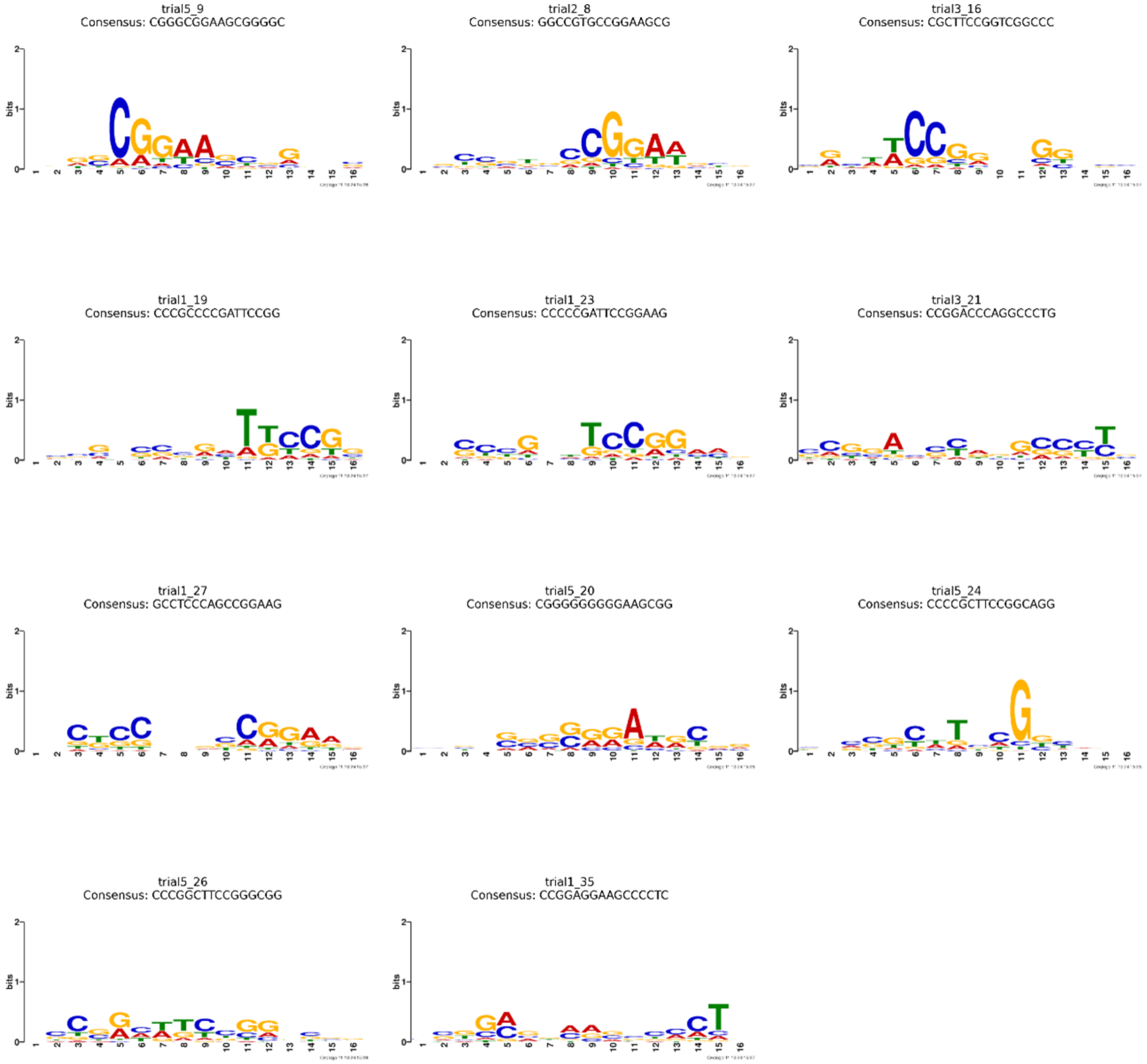

Supplementary Fig. 15: Reproducible unannotated motifs extracted from models trained on ELK4 signals in HeLa-S3 cell line. Motifs are arranged in descending order of information content, with each panel showing the trial number (1-5), motif ID, and consensus sequence.

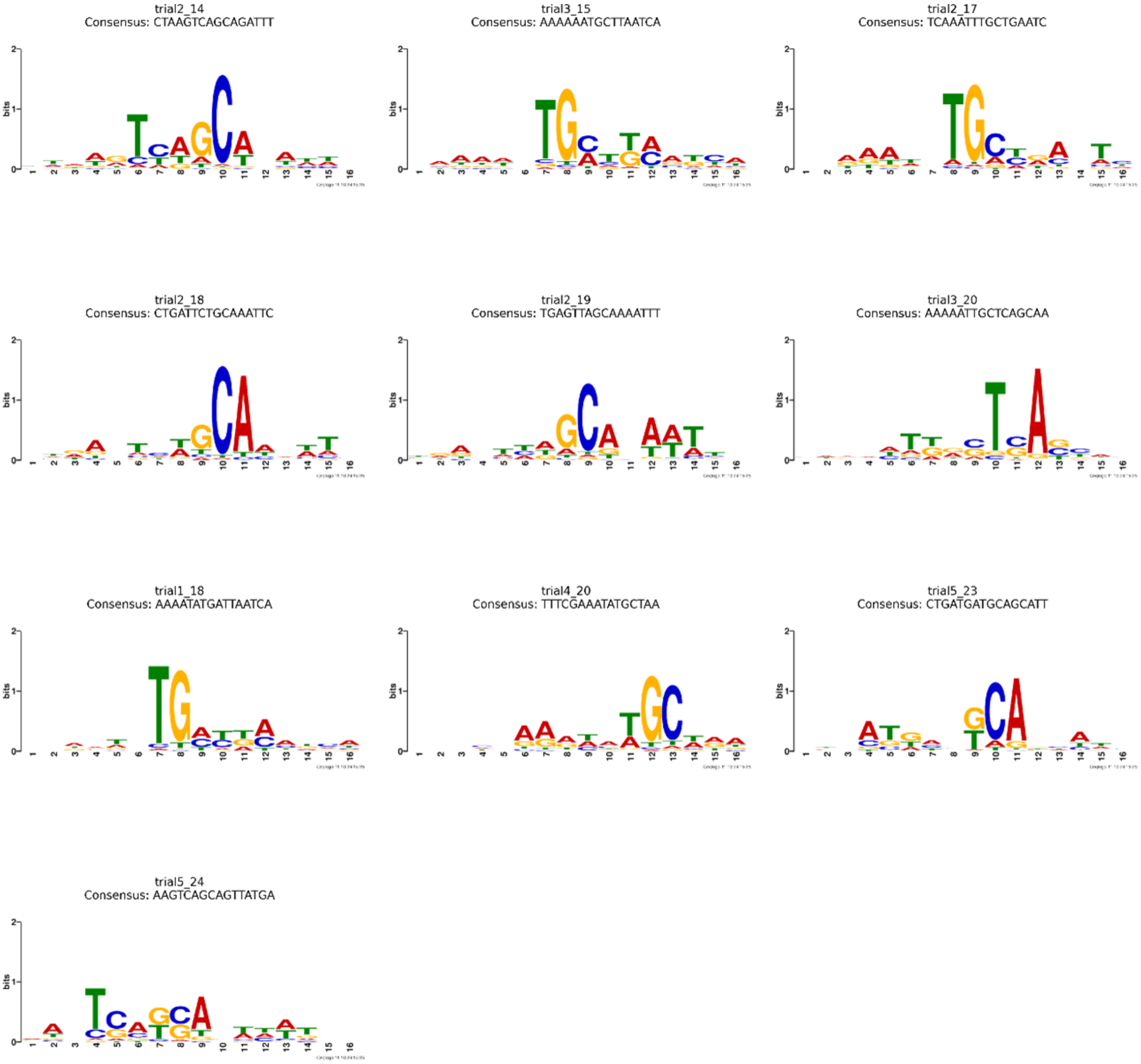

Supplementary Fig. 16: Reproducible unannotated motifs extracted from models trained on MAFF signals in HeLa-S3 cell line. Motifs are arranged in descending order of information content, with each panel showing the trial number (1-5), motif ID, and consensus sequence.

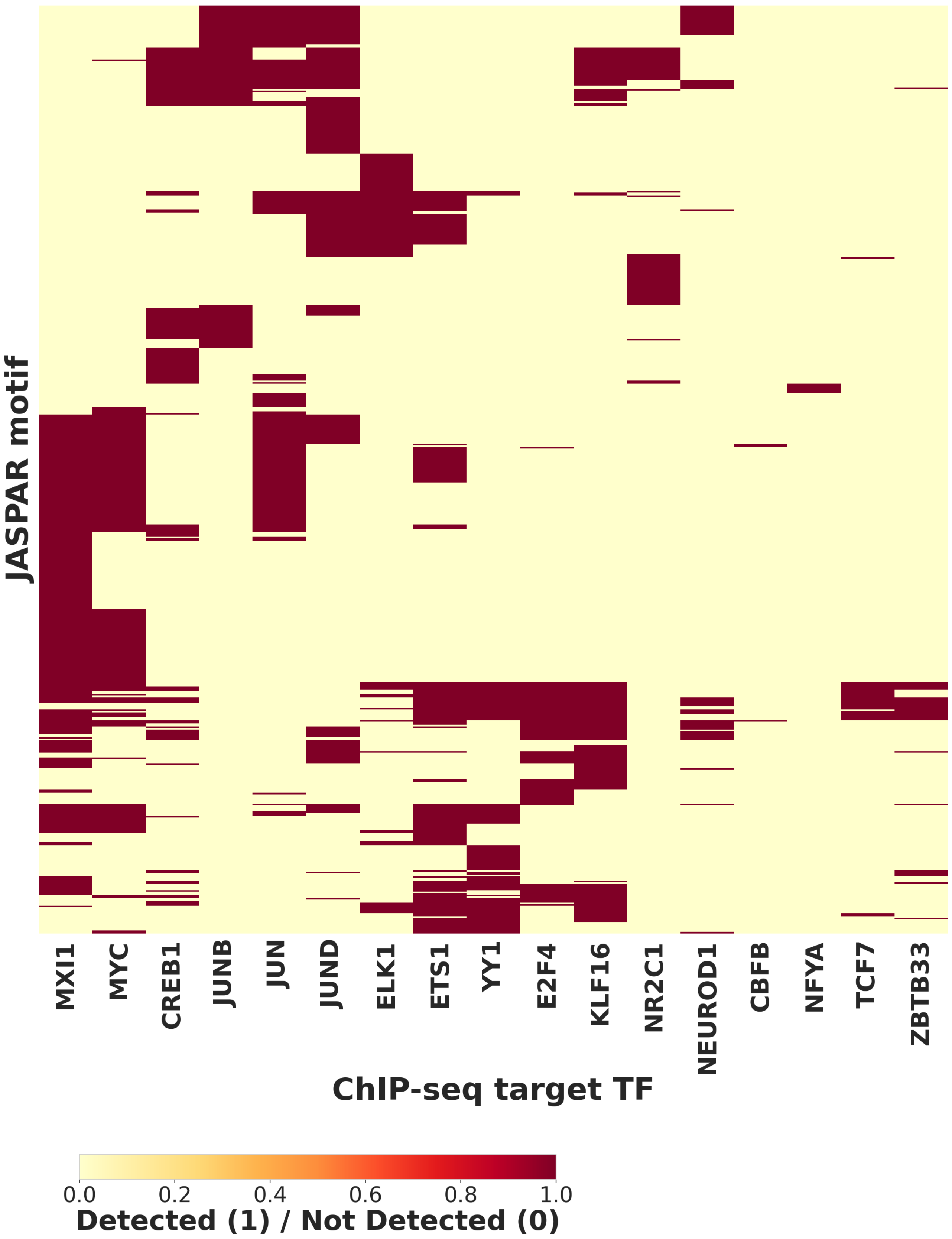

Supplementary Fig. 17: Binary heatmap of JASPAR TF detection in K562 cell line. Binary heatmap showing reproducible TF detection in K562 cells. The horizontal axis represents 601 JASPAR TFs detected by QTFPred in at least one primary TF.

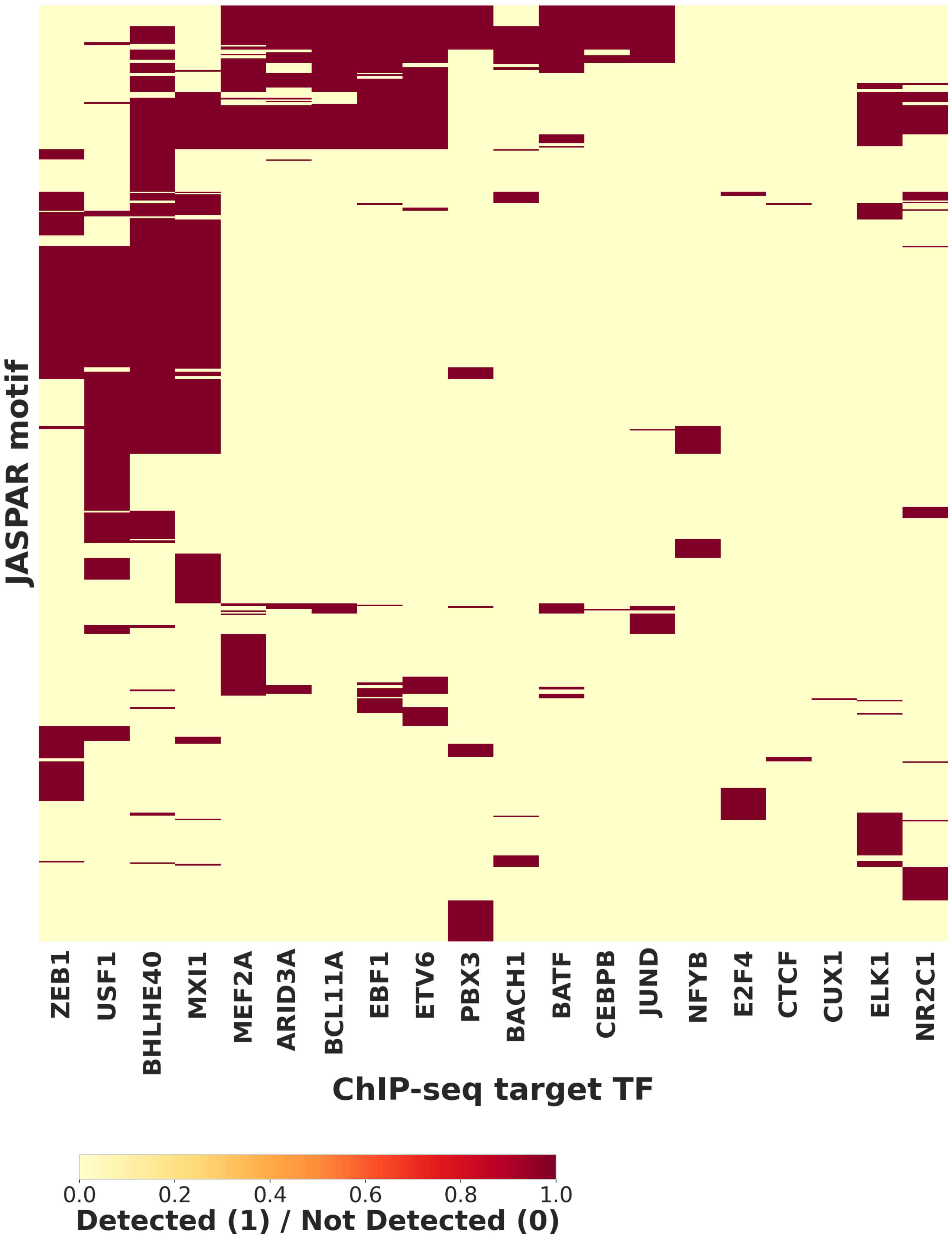

Supplementary Fig. 18: Binary heatmap of JASPAR TF detection in GM12878 cell line. Binary heatmap showing reproducible TF detection in GM12878 cells. The horizontal axis represents 639 JASPAR TFs detected by QTFPred in at least one primary TF.

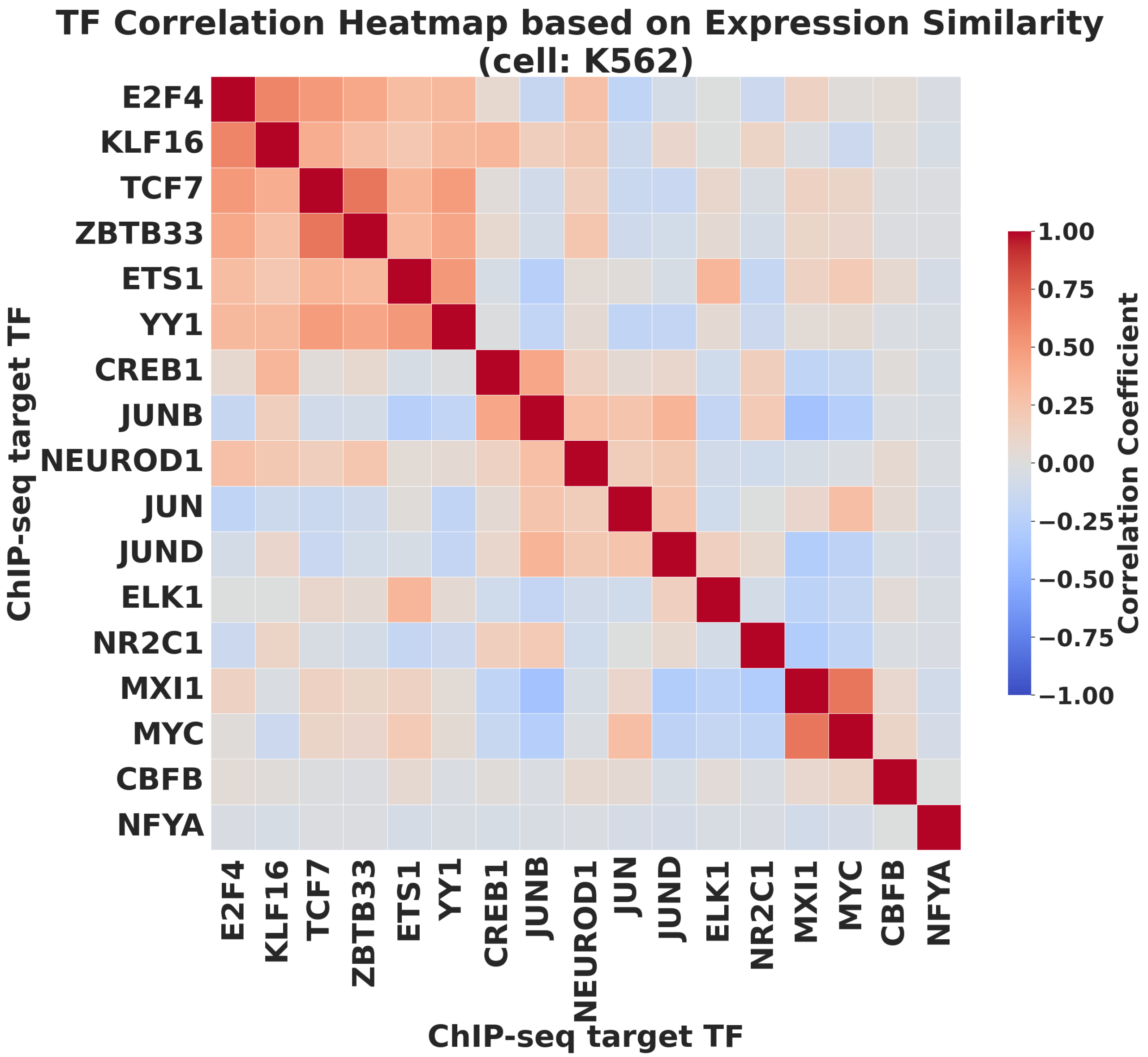

Supplementary Fig. 19: Correlation matrix of co-localization patterns in K562 cell line. The matrix is organized using hierarchical clustering (Ward's method). Based on dendrogram distance metrics (threshold 0.75), the following functional similarity pairs were identified: {TCF7, ZBTB33, ETS1, YY1}, {E2F4, KLF16}, {CREB1, JUNB}, {JUN, JUND}, and {MXI1, MYC}.

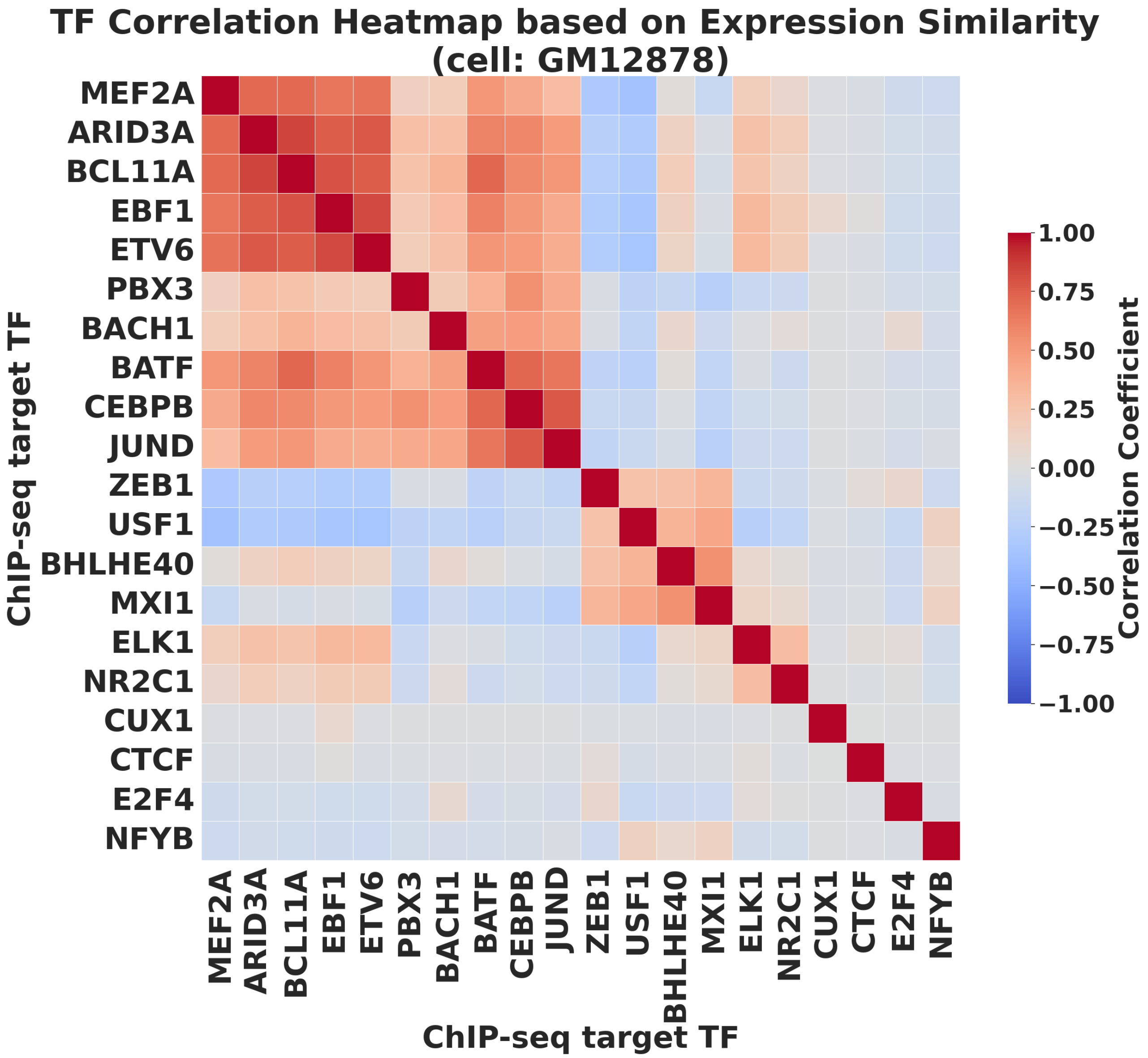

Supplementary Fig. 20: Correlation matrix of co-localization patterns in GM12878 cell line. The matrix is organized using hierarchical clustering (Ward's method). Based on dendrogram distance metrics (threshold 0.75), the following functional similarity pairs were identified: {MEF2A, ARID3A, BCL11A, EBF1, ETV6}, {PBX3, BACH1, BATF, CEBPB, JUND}, {USF1, BHLHE40, MXI1}, and {ELK1, NR2C1}.

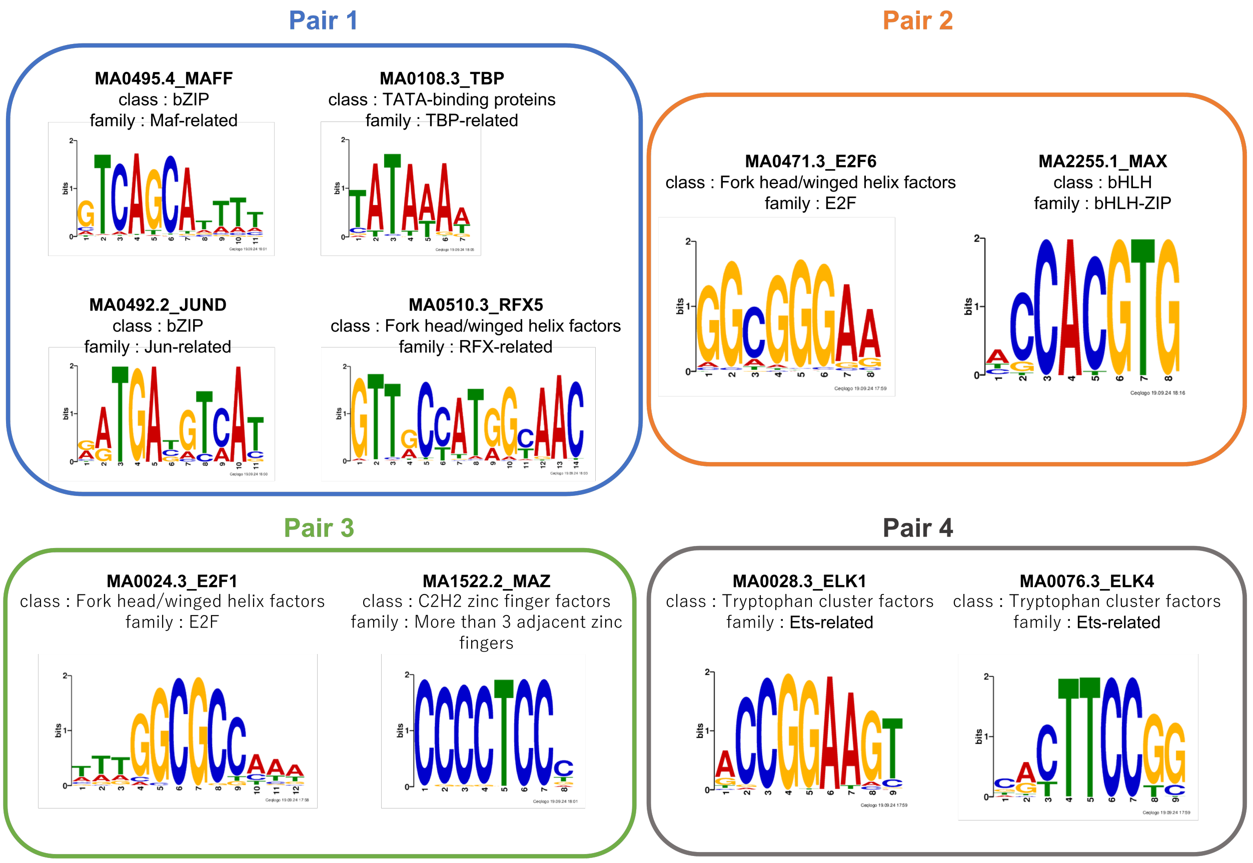

Supplementary Fig. 21: Functionally similar TF pairs predicted by QTFPred in HeLa-S3 cell line. The bold text above each logo shows the TF name and corresponding JASPAR motif ID, with TF family indicated below.

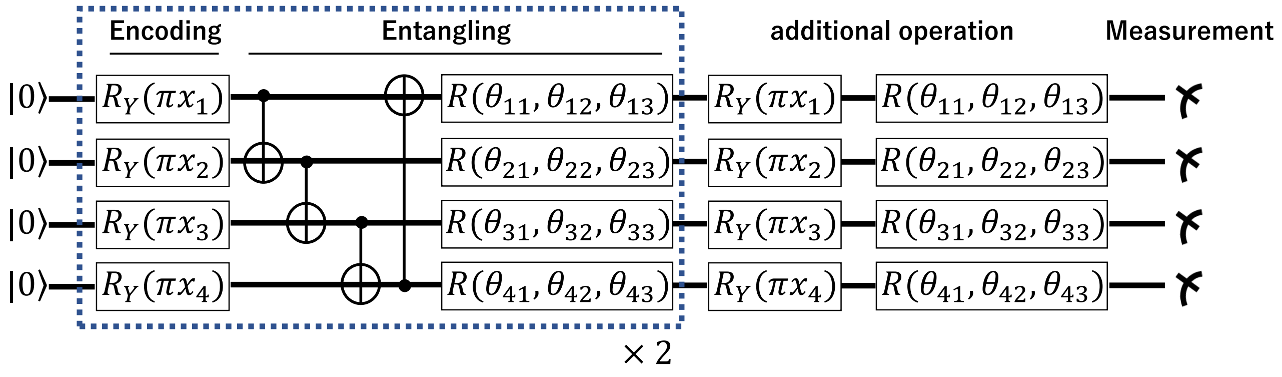

Supplementary Fig. 22: Structure of parametric quantum circuits used in QTFPred. $R_Y(\theta)$ gate converts classical information x into quantum information (angle encoding). Sequential application of CNOT gates and single-qubit rotation gates generates quantum entanglement. The Encoding and Entangling steps are repeated, followed by additional transformations before measurement in the Z basis. Single-qubit rotation gates contain trainable parameters. The total number of trainable parameters is 36.

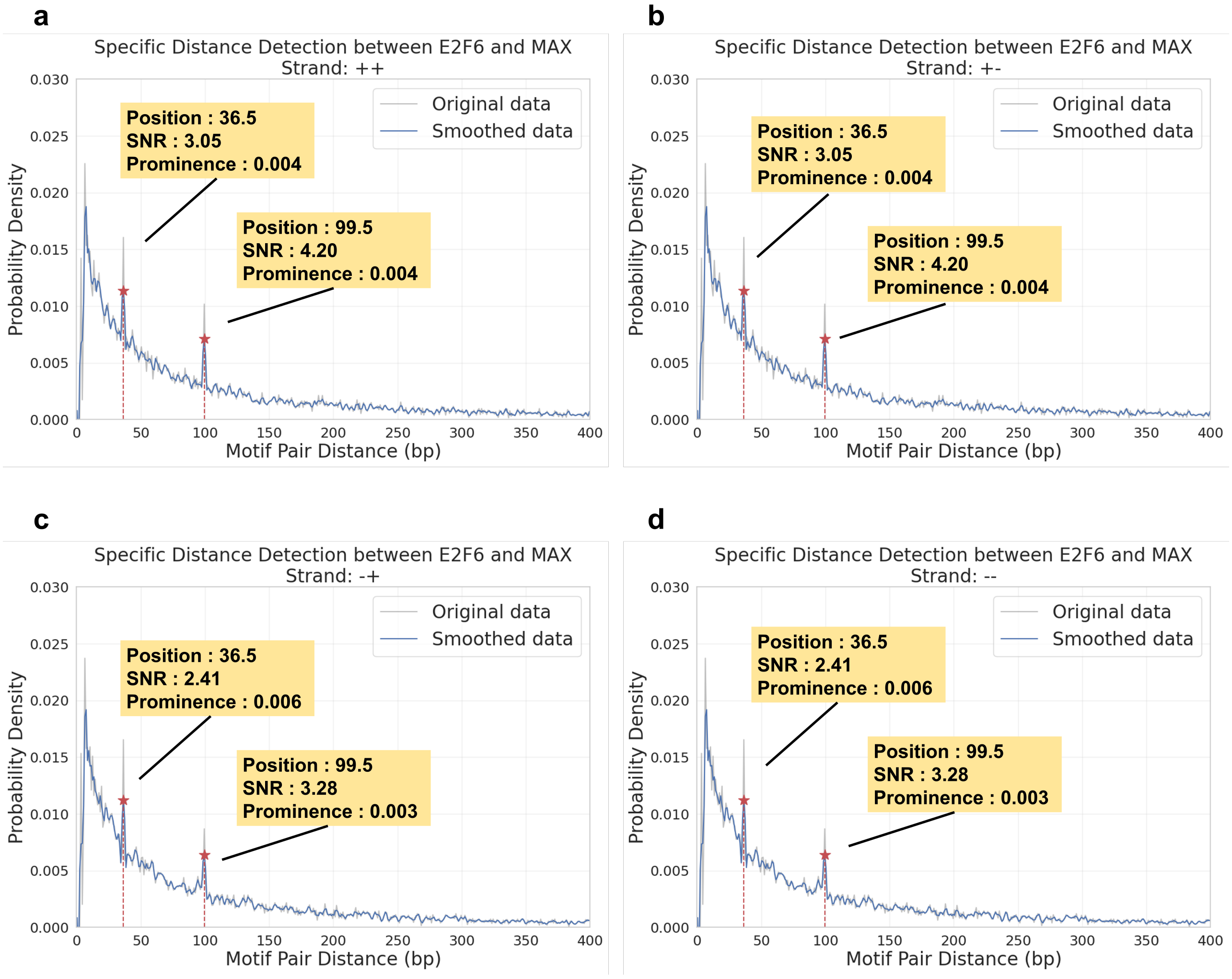

Supplementary Fig. 23: Distance distribution analysis between strand-specific E2F6 and MAX binding motifs in the ChIP-seq peak regions of MAX. Each panel shows original data (gray) and smoothed data (blue line). Red asterisks indicate significant peaks, with orange boxes displaying peak characteristics (position, SNR, prominence). Analysis parameters are shown in the Supplementary Method 14. (a) +/+. (b) +/−. (c) −/+. (d) −/−.

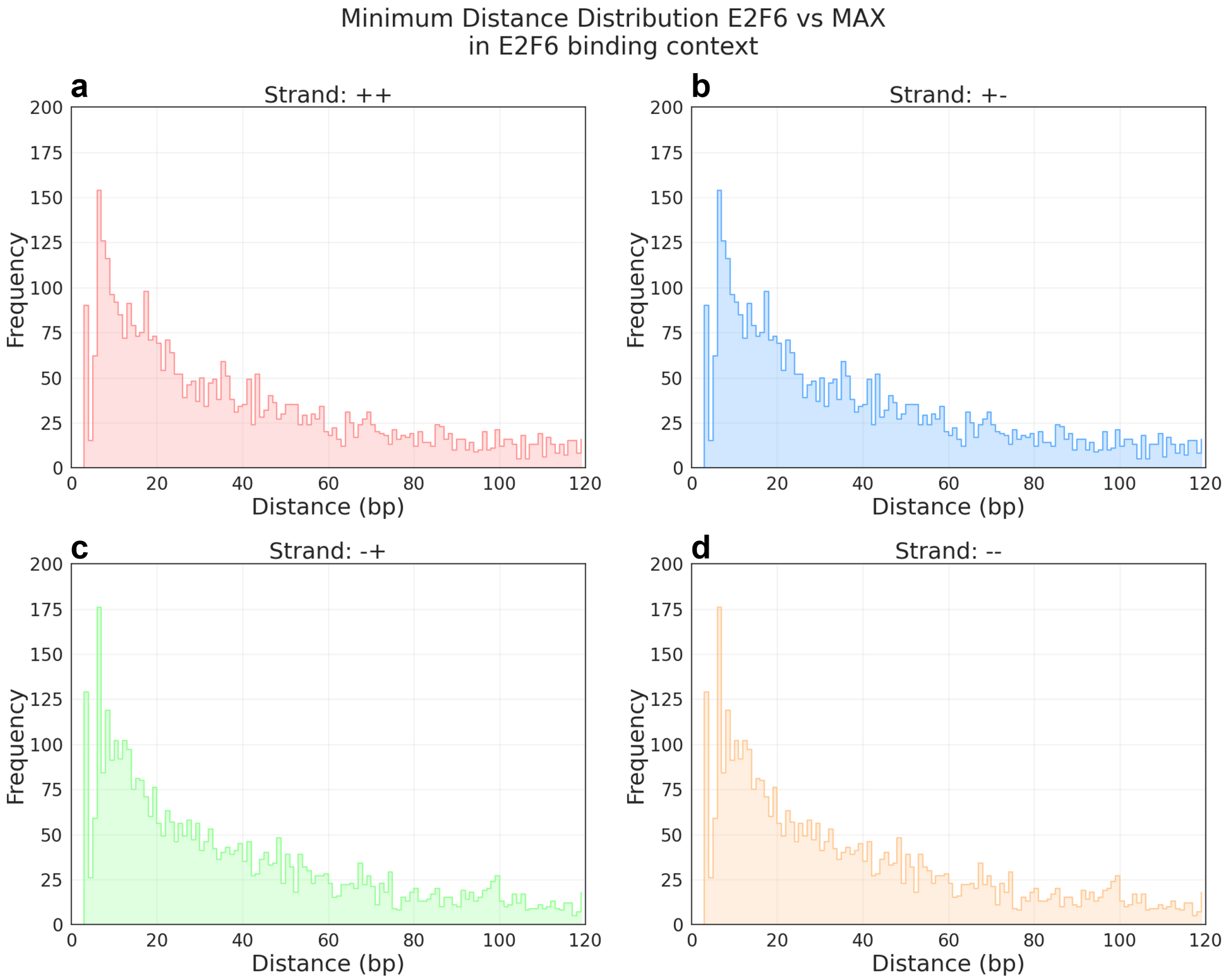

Supplementary Fig. 24: Frequency distribution of distances between strand-specific E2F6 and MAX binding motifs in the ChIP-seq peak regions of E2F6. (a) +/+. (b) +/−. (c) −/+. (d) −/−.

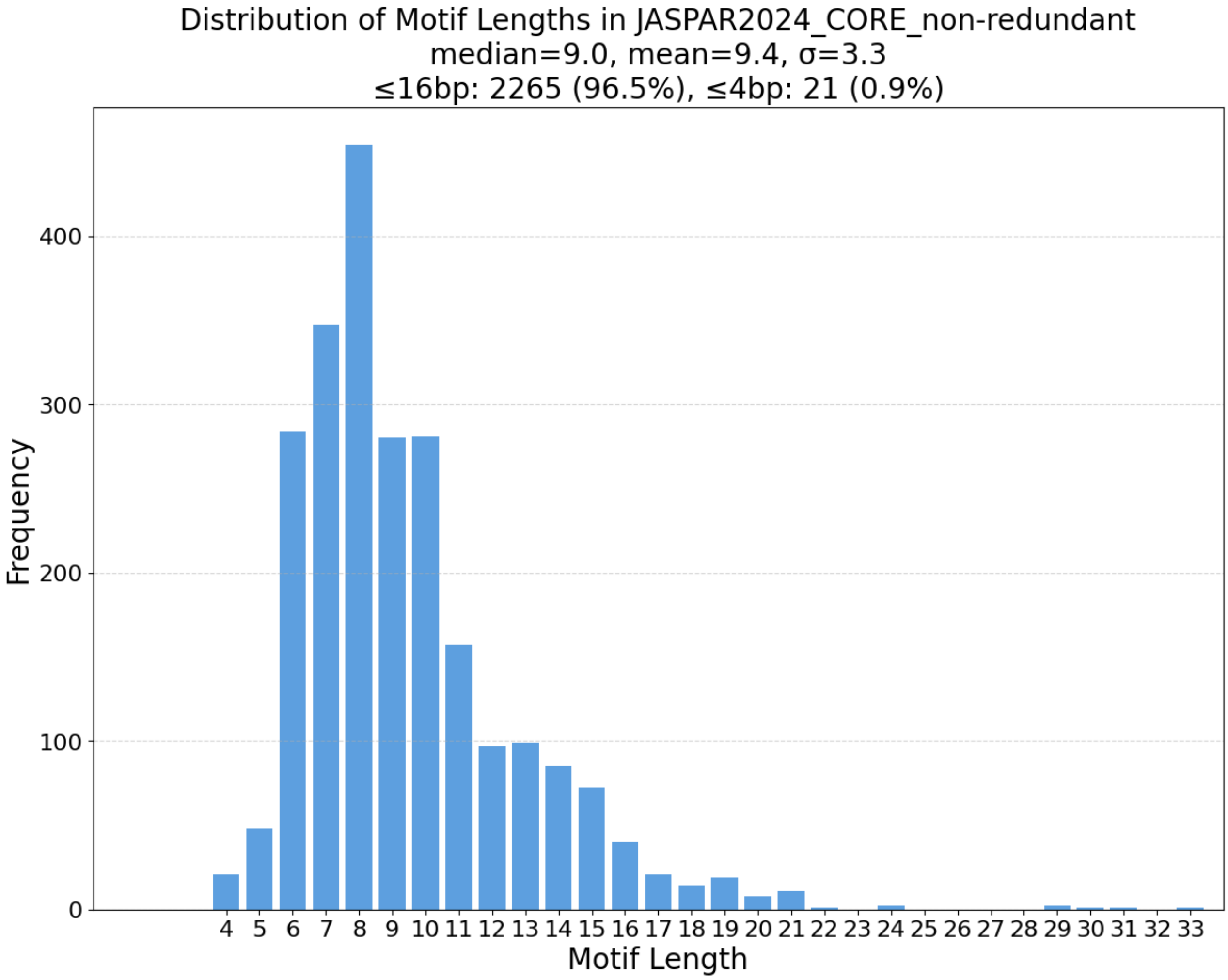

Supplementary Fig. 25: Length distribution analysis of motifs in the JASPAR2024 CORE non-redundant database.

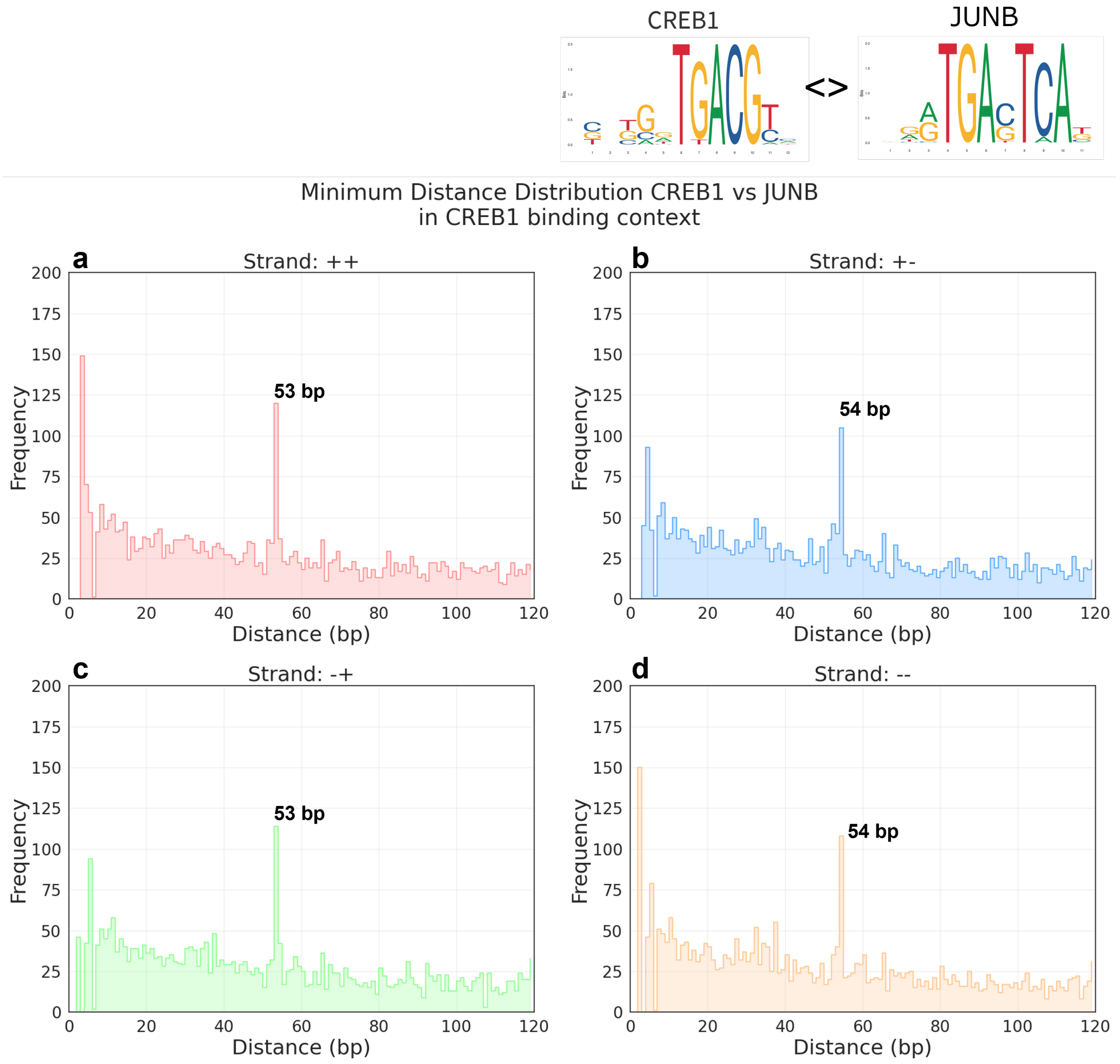

Supplementary Fig. 26: Frequency distribution of distances between strand-specific CREB1 and JUNB binding motifs in the ChIP-seq peak regions of CREB1. (a) +/+. (b) +/−. (c) −/+. (d) −/−.

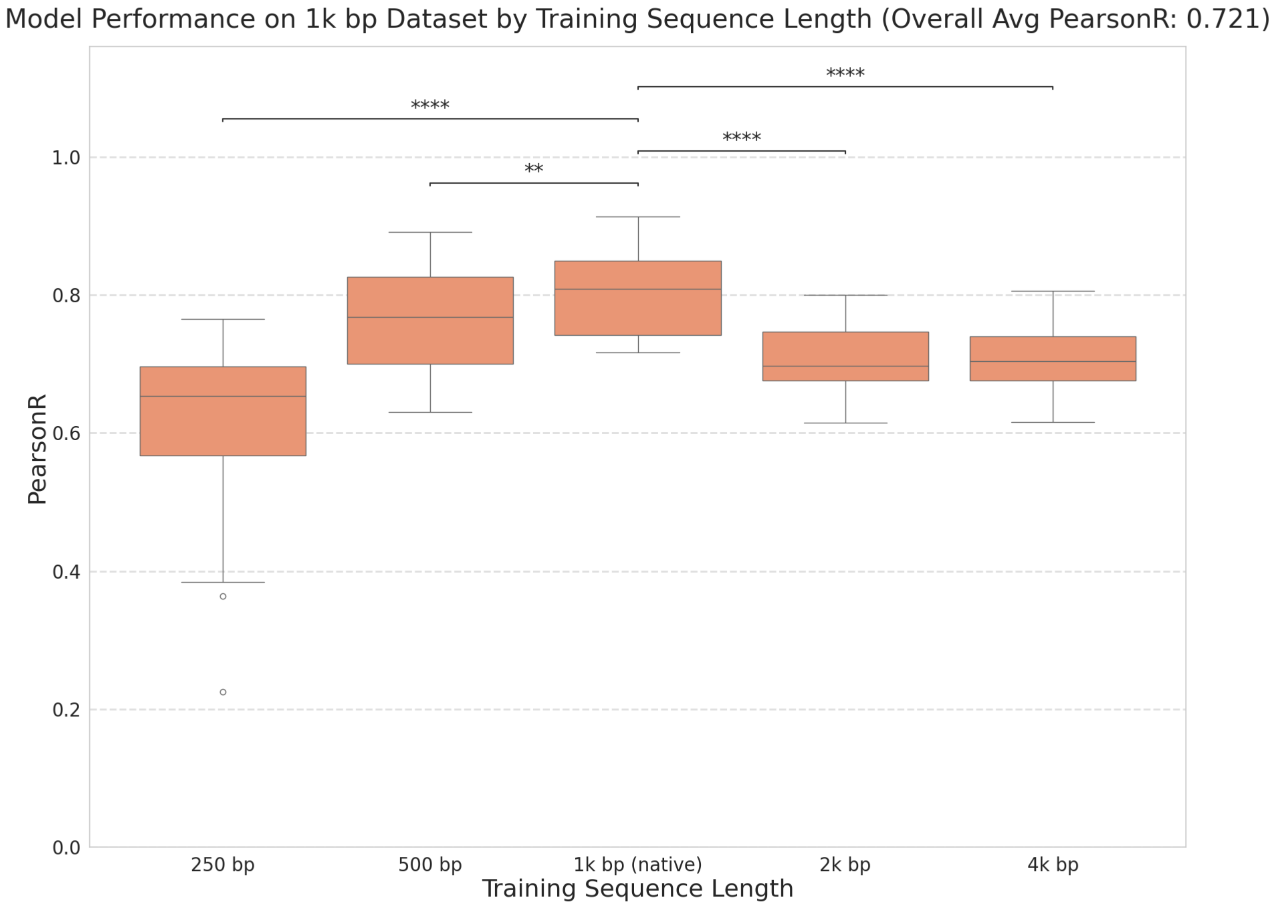

Supplementary Fig. 27: Input sequence length dependency analysis for QTFPred signal prediction. Box plots show performance across five different sequence lengths (250 bp, 500 bp, 1k bp, 2k bp, 4k bp) using six TFs from HeLa-S3 cell line (ELK1, ELK4, REST, MAZ, JUND, CTCF).

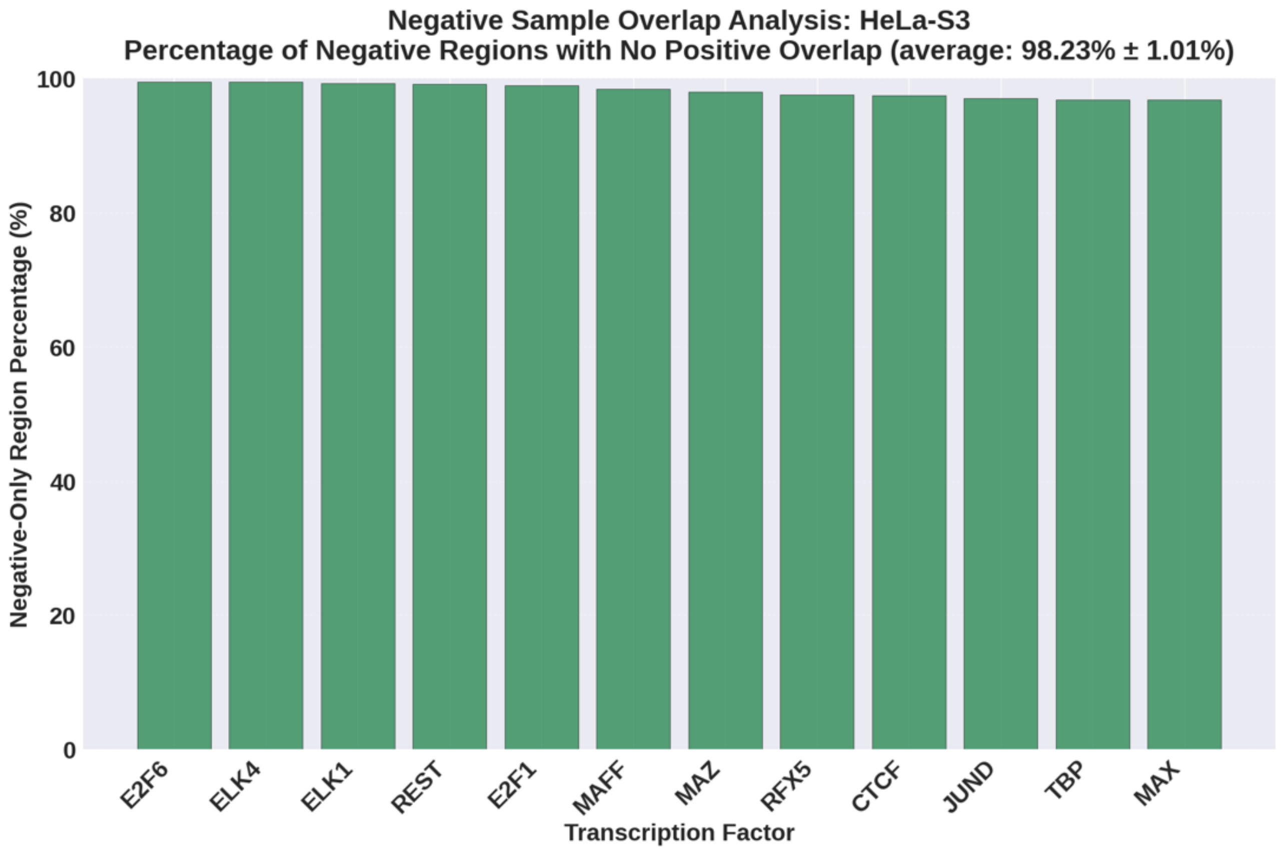

Supplementary Fig. 28: Negative sample overlap analysis across 12 TFs in HeLa-S3 cell line. Bar chart displays the percentage of negative training samples with no overlap to positive binding regions for each transcription factor.

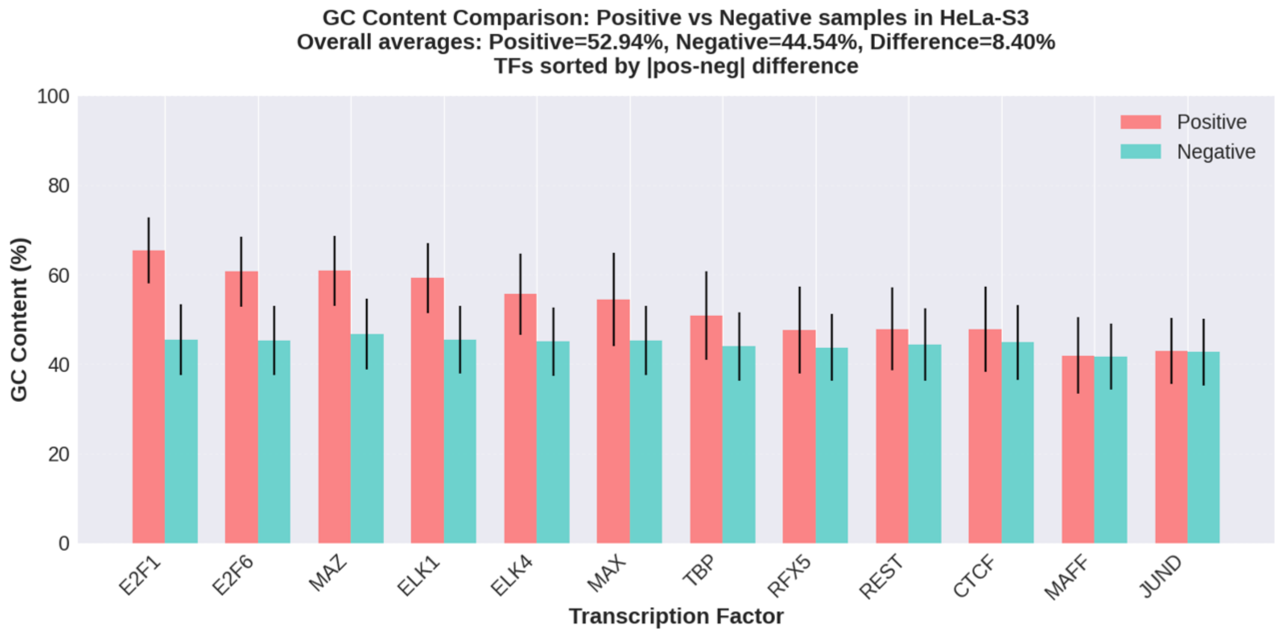

Supplementary Fig. 29: GC content comparison between positive and negative training samples across 12 TFs in HeLa-S3 cell line. Bar chart shows GC content percentages for positive and negative samples, with TFs sorted by the magnitude of difference between sample types.

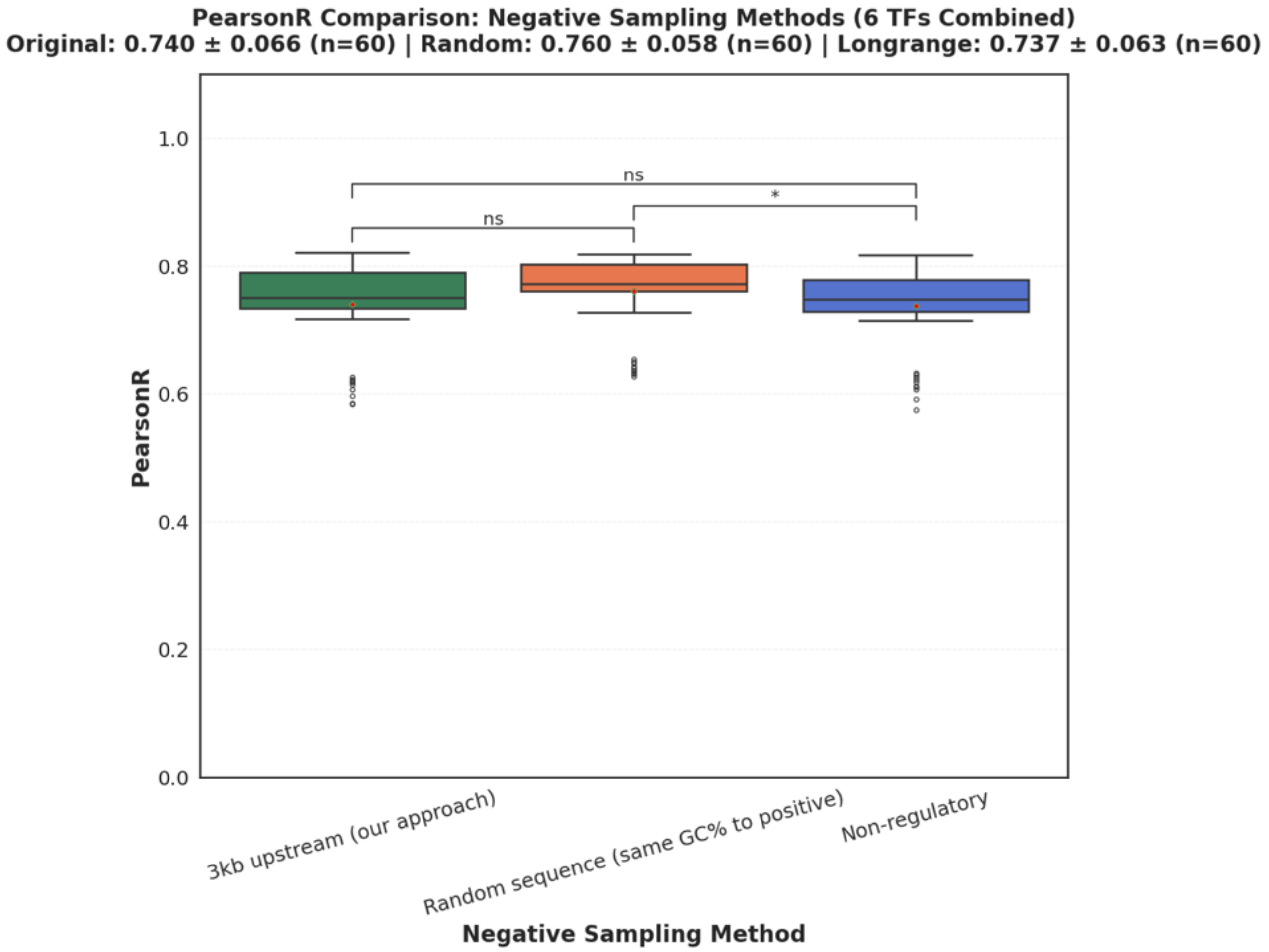

Supplementary Fig. 30: Comparison of three negative sampling strategies for QTFPred signal prediction across six representative TFs in HeLa-S3 cell line. Box plots compare original 3kb upstream approach, random artificial sequences with matched GC content to positive samples, and non-regulatory genomic regions sampled from distant locations. Each method evaluated across ELK1, ELK4, REST, MAZ, JUND, and CTCF datasets.

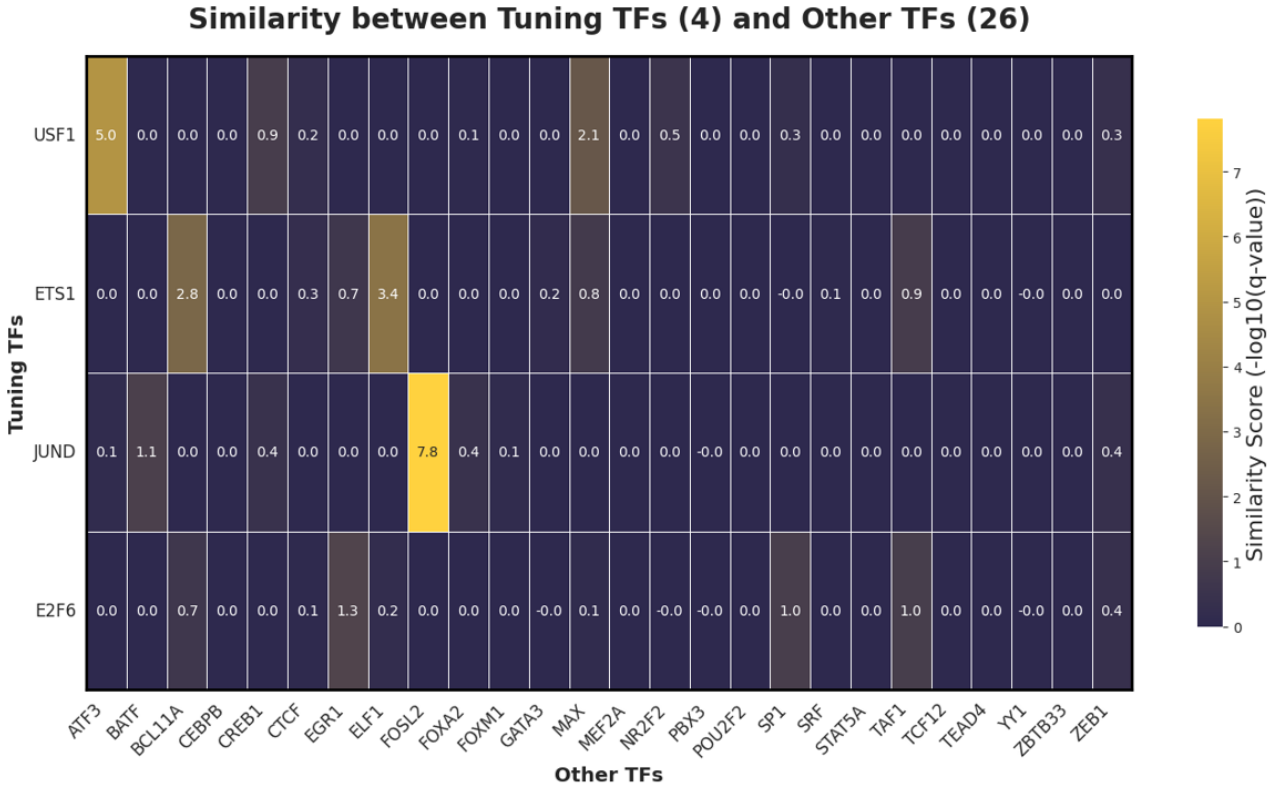

Supplementary Fig. 31: Motif similarity heatmap between hyperparameter tuning TFs and evaluation TFs using TOMTOM analysis. Heatmap displays similarity scores between four tuning TFs (USF1, ETS1, JUND, E2F6) from A549 cell line and 26 evaluation TFs from binary classification datasets.

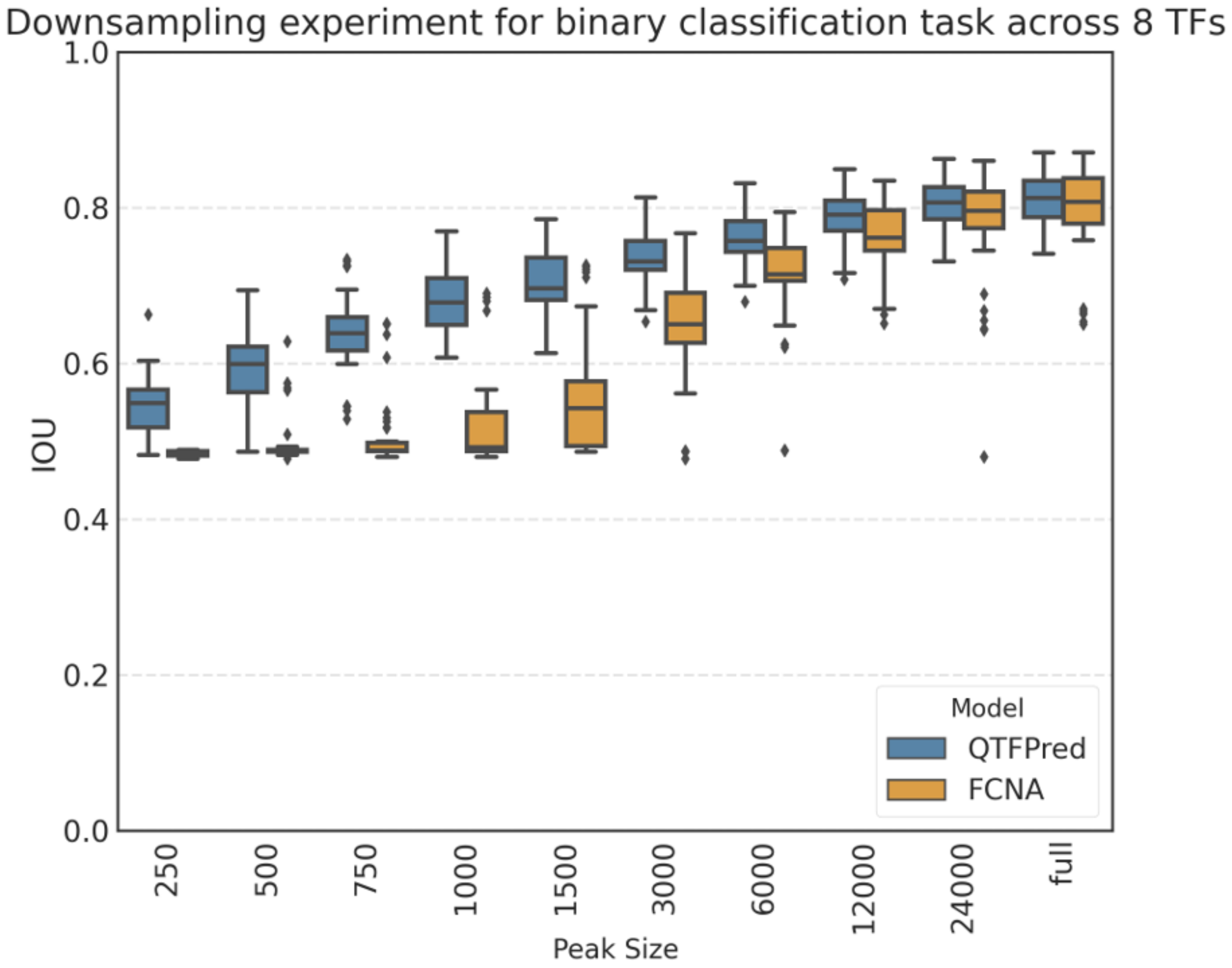

Supplementary Fig. 32: Down-sampling experiment for binary TFBS classification across eight TFs from GM12878 and MCF7 cell lines. Box plots show QTFPred and FCNA model performance across varying peak sizes from 250 to full datasets for binary classification tasks.

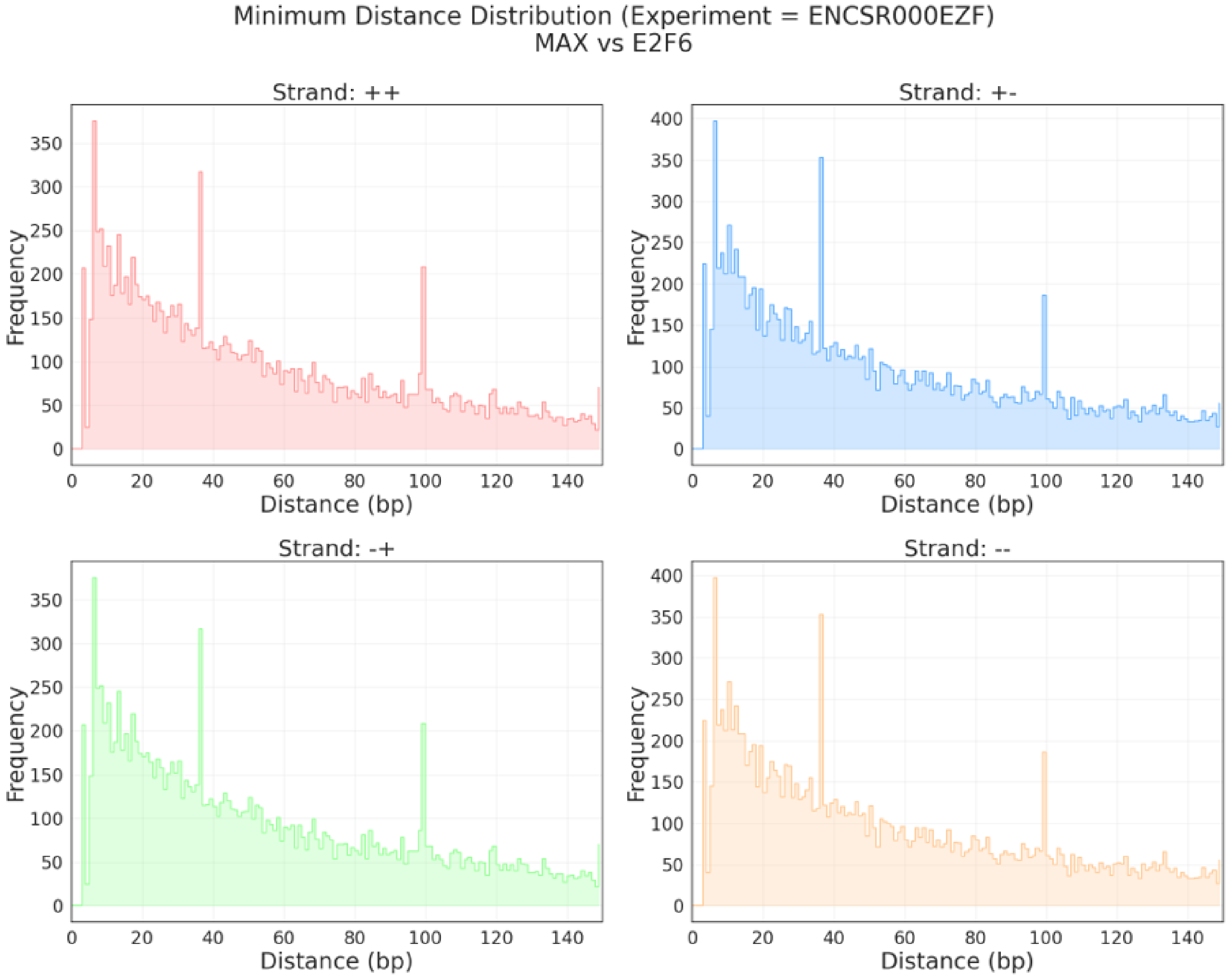

Supplementary Fig. 33: Distance distribution analysis between E2F6 and MAX motifs using independent ChIP-seq experiment (ENCSR000EZF) in HeLa-S3 cell line. Four panels show minimum distance distributions for different strand orientations (++, +-, -+, –) within MAX binding peaks.

Cell Line	TF	Peak Counts	DBD Family	Data Source
A549(15)	PBX3	7,122	Homeodomain	FCNA*
	ZBTB33	9,112	C2H2 ZF	FCNA*
	TAF1	11,004	Unknown	FCNA*
	TEAD4	12,423	TEA	FCNA*
	ELF1	14,555	Ets	FCNA*
	YY1	20,176	C2H2 ZF	FCNA*
	GATA3	20,017	GATA	FCNA*
	CEBPB	26,320	bZIP	FCNA*
	CREB1	27,555	bZIP	FCNA*
	MAX	32,667	bHLH	FCNA*
	CTCF	35,572	C2H2 ZF	FCNA*
	SP1	35,874	C2H2 ZF	FCNA*
	FOSL2	37,115	bZIP	FCNA*
	TCF12	39,382	bHLH	FCNA*
	FOXA2	45,498	Forkhead	FCNA*
GM12878(20)	ATF3	2,921	bZIP	FCNA*
	ETS1	8,436	Ets	FCNA*
	USF1	9,268	bHLH	FCNA*
	SRF	10,420	MADS box	FCNA*
	CEBPB	12,247	bZIP	FCNA*
	ZBTB33	13,571	C2H2 ZF	FCNA*
	CREB1	15,758	bZIP	FCNA*
	ZEB1	17,451	C2H2 ZF; Homeodomain	FCNA*
	EGR1	17,468	C2H2 ZF	FCNA*
	TAF1	17,585	Unknown	FCNA*
	MEF2A	19,145	MADS box	FCNA*
	STAT5A	30,266	STAT	FCNA*
	ELF1	31,332	Ets	FCNA*
	BCL11A	31,672	C2H2 ZF	FCNA*
	PBX3	34,215	Homeodomain	FCNA*
	SP1	37,020	C2H2 ZF	FCNA*
	TCF12	40,188	bHLH	FCNA*
	YY1	43,592	C2H2 ZF	FCNA*
	POU2F2	45,368	Homeodomain; POU	FCNA*
	BATF	52,216	bZIP	FCNA*
MCF7(14)	TAF1	4,093	Unknown	FCNA*
	TCF12	4,861	bHLH	FCNA*
	TEAD4	4,873	TEA	FCNA*
	FOXM1	5,550	Forkhead	FCNA*
	SRF	5,572	MADS box	FCNA*
	EGR1	6,348	C2H2 ZF	FCNA*
	JUND	7,500	bZIP	FCNA*
	GATA3	9,854	GATA	FCNA*
	ELF1	15,042	Ets	FCNA*
	FOSL2	16,157	bZIP	FCNA*
	MAX	29,929	bHLH	FCNA*
	NR2F2	31,626	Nuclear receptor	FCNA*
	CEBPB	34,154	bZIP	FCNA*
	CTCF	44,480	C2H2 ZF	FCNA*

*FCNA: Downloaded from `https://github.com/turningpoint1988/FCNA`
DBD families based on [1]

Supplementary Table 1: List of 49 TFs used for binary TFBS classification and their peak counts across three cell lines

Cell Line	TF	Peak Counts	ENCODE Data Source
HeLa-S3(12)	ELK1	6,091	Experiment ID = ENCSR000ECI
	E2F6	6,649	Experiment ID = ENCSR000EVK
	ELK4	7,251	Experiment ID = ENCSR000EVI
	E2F1	11,219	Experiment ID = ENCSR000EVM
	REST	11,963	Experiment ID = ENCSR000BMN
	MAZ	16,565	Experiment ID = ENCSR000ECL
	TBP	23,582	Experiment ID = ENCSR000EDD
	RFX5	24,379	Experiment ID = ENCSR000ECX
	MAFF	25,548	Experiment ID = ENCSR140DSL
	MAX	34,097	Experiment ID = ENCSR000ECN
	JUND	34,107	Experiment ID = ENCSR000EDH
	CTCF	41,927	Experiment ID = ENCSR000DUB
K562(17)	ELK1	3,793	Experiment ID = ENCSR000EFU
	JUNB	4,147	Experiment ID = ENCSR525VAT
	NFYA	4,571	Experiment ID = ENCSR000EGR
	TCF7	5,501	Experiment ID = ENCSR863KUB
	CBFB	7,326	Experiment ID = ENCSR116NDV
	MXI1	9,081	Experiment ID = ENCSR000EGZ
	E2F4	9,109	Experiment ID = ENCSR000EWL
	MYC	9,959	Experiment ID = ENCSR744JJU
	ETS1	13,775	Experiment ID = ENCSR000BKQ
	NEUROD1	14,155	Experiment ID = ENCSR986CDX
	NR2C1	15,072	Experiment ID = ENCSR742IDN
	CREB1	17,621	Experiment ID = ENCSR000BSO
	KLF16	17,643	Experiment ID = ENCSR397DQC
	JUN	21,522	Experiment ID = ENCSR000EFS
	YY1	30,726	Experiment ID = ENCSR000BMH
	JUND	47,180	Experiment ID = ENCSR000EGN
	ZBTB33	58,537	Experiment ID = ENCSR876GXA
GM12878(20)	E2F4	4,375	Experiment ID = ENCSR000DYY
	CEBPB	5,174	Experiment ID = ENCSR000BRX
	ELK1	7,245	Experiment ID = ENCSR000DZB
	CUX1	7,284	Experiment ID = ENCSR000DYR
	JUND	7,602	Experiment ID = ENCSR000DYS
	ZEB1	7,619	Experiment ID = ENCSR000BND
	USF1	8,461	Experiment ID = ENCSR000BGI
	NR2C1	10,486	Experiment ID = ENCSR784VIQ
	PBX3	12,587	Experiment ID = ENCSR000BGR
	NFYB	14,678	Experiment ID = ENCSR000DNM
	ARID3A	17,400	Experiment ID = ENCSR778UBR
	BCL11A	21,232	Experiment ID = ENCSR000BHA
	MXI1	21,737	Experiment ID = ENCSR000DZI
	ETV6	21,783	Experiment ID = ENCSR626VUC
	BACH1	22,354	Experiment ID = ENCSR636MKU
	MEF2A	22,588	Experiment ID = ENCSR000BKB
	BATF	37,158	Experiment ID = ENCSR000BGT
	CTCF	41,952	Experiment ID = ENCSR000AKB
	EBF1	42,055	Experiment ID = ENCSR000BGU
	BHLHE40	49,176	Experiment ID = ENCSR987MTA

Supplementary Table 2: List of 49 TFs used for signal prediction task and their peak counts across three cell lines

Model	Hyperparameter	Value	Search Space / Source
QTFPred	Batch size	76	[20, 140], Optuna
	Learning rate	5.30e-3	[1e-5, 1e-1], Optuna
	Weight decay	2.20e-5	[1e-5, 1e-1], Optuna
	Dropout	2.30e-1	[0.1, 0.8], Optuna
	Kernel size of decoder	7	[3, 5, 7], Optuna
	Activation	GELU	[ELU, SiLU, GELU], Optuna
	Bottleneck dim.	50	[1, 50], Optuna
	Ratio loss	5.50e-1	[0.1, 0.95], Optuna
	Weight initialization	Xavier	Fixed
FCN, FCNA	Batch size	100	Fixed
	Learning rate	1.00e-3	Fixed
	Weight decay	5.00e-4	Fixed
	Dropout	2.00e-1	Fixed
	Kernel size of decoder	3	Fixed
	Activation	ELU	Fixed
	Bottleneck dim.	1	Fixed
	Ratio loss	3.00e-1	Fixed
	Weight initialization	Xavier	Fixed

Supplementary Table 3: Hyperparameters for binary classification task. Bottleneck dim. is the output dimension of adaptive average pooling in the bottleneck layer. Ratio loss is the selection ratio of hard negative samples in the hard negative mining method adopted in the loss design for the binary prediction model

Model	Hyperparameter	Value	Search Space / Source
QTFPred	Batch size	66	[20, 120], Optuna
	Learning rate	2.60e-3	[1e-5, 1e-1], Optuna
	Weight decay	7.20e-3	[1e-5, 1e-1], Optuna
	Dropout	1.47e-1	[0.1, 0.8], Optuna
	Kernel size of decoder	7	[3, 5, 7], Optuna
	Activation	GELU	[ELU, SiLU, GELU], Optuna
	Bottleneck dim.	34	[1, 50], Optuna
	Dropout of GRU unit	1.33e-1	[0.1, 0.8], Optuna
	Beta1	9.00e-1	Fixed
	Beta2	9.99e-1	Fixed
	Weight initialization	Xavier	Fixed
FCNsignal	Learning rate	1.60e-3	[1e-5, 1e-1], Optuna
	Weight decay	4.50e-4	[1e-5, 1e-1], Optuna
	Beta1	9.14e-1	[0.9, 0.999], Optuna
	Beta2	9.36e-1	[0.9, 0.999], Optuna
	Batch size	500	Fixed
	Bottleneck dim.	1	Fixed
	Dropout	2.00e-1	Fixed
	Dropout of GRU unit	2.00e-1	Fixed
	Activation	ELU	Fixed
	Weight initialization	Xavier	Fixed
BPNet	Learning rate	6.80e-3	[1e-5, 1e-1], Optuna
	Weight decay	4.90e-3	[1e-5, 1e-1], Optuna
	Beta1	9.09e-1	[0.9, 0.999], Optuna
	Beta2	9.48e-1	[0.9, 0.999], Optuna
	Batch size	500	Fixed
	Activation	ReLU	Fixed
	Weight initialization	Xavier	Fixed

Supplementary Table 4: Hyperparameters for signal prediction task.

Supplementary Results

Supplementary Result 1: Functional TF pairs in multiple cell lines

We identified specific TF pairs with functional relationships in K562 and GM12878 cell lines using "TF detection binary heatmap based on reproducible motif matches" (Supplementary Figs. 17-18). Correlation analysis revealed multiple functionally similar TF pairs within these cell lines (Supplementary Figs. 19-20). Notably, we observed a specific 53-54 bp distance constraint between CREB1 and JUNB motifs in the K562 cell line (Supplementary Fig. 26), distinct from the distance patterns observed in the E2F6-MAX pair, suggesting unique spatial constraints govern different TF interactions.

Supplementary Result 2: Identification of unannotated regulatory motifs

Deep learning models can extract both known TF representations and latent patterns that contribute to prediction accuracy. Consistent with previous studies[2, 3], QTFPred learned unannotated motifs alongside known binding patterns. Analysis of models trained on three TFs (JUND, ELK4, MAFF) in the HeLa-S3 cell line revealed highly informative and reproducible unannotated motifs (Supplementary Figs. 14-16) with information content comparable to annotated motifs (Supplementary Method 11). For instance, with JUND as the primary TF, we identified a specific motif consensus (CATTCCT) fundamentally different from the JASPAR-registered JUND consensus (CACTTCCGG, Supplementary Fig. 14). These unannotated sequence patterns may represent either alternative binding sites for the primary TF or recognition sites for cooperative factors that interact with the primary TF. Further research is needed to determine their specific roles in transcriptional regulation.

Supplementary Result 3: Negative Sampling Method Selection

Our primary negative sampling approach extracts 1,000 bp sequences from regions located 3,000 bp upstream of each ChIP-seq peak, as described in the FCNsignal pipeline. To address potential concerns regarding regulatory region contamination and sequence composition bias, we conducted a comprehensive evaluation of this approach against two alternative negative sampling strategies.

First, we assessed the risk of false negative contamination by analyzing overlap between negative samples and positive binding regions (Supplementary Fig. 28). Across all 12 TFs in HeLa-S3, negative samples showed virtually no overlap with positive regions, with 98.23 $\pm$ 1.019% of negative regions containing no positive overlap, confirming effective separation between positive and negative training data.

However, sequence composition analysis revealed GC content differences between positive and negative samples for several TFs (Supplementary Fig. 29), with positive samples showing higher GC content (52.94 %) compared to negative samples (44.54 %), representing an 8.40 % average difference that varied across TFs. To evaluate whether these composition biases could influence model performance and evaluation outcomes, we implemented two alternative negative sampling strategies: (1) artificial random sequences generated to match the exact ACGT nucleotide composition of positive samples, thereby controlling for sequence composition while eliminating any potential regulatory activity, and (2) genomic sequences sampled from regions located $>$10 kb away from any known binding sites, providing real genomic context while removing the fixed-distance sampling bias inherent in the upstream approach.

Performance comparison across six representative TFs using these three negative sampling approaches showed no significant differences (Supplementary Fig. 30; our approach: 0.74 $\pm$ 0.066, artificial sequences: 0.76 $\pm$ 0.058, distant genomic regions: 0.74 $\pm$ 0.064; $p > 0.05$ for pairwise comparisons between our approach and alternatives), indicating that negative sampling strategy choice does not substantially impact QTFPred's performance evaluation and that our 3,000 bp upstream approach provides valid negative samples for transcription factor binding prediction tasks.

Supplementary Result 4: Input Sequence Length Selection

To systematically evaluate the effect of training sequence length on model performance, we conducted sequence length dependency analysis (Supplementary Fig. 27) using six representative TFs from HeLa-S3 (ELK1, ELK4, REST, MAZ, JUND, CTCF). Models were trained with five different sequence lengths (250 bp, 500 bp, 1,000 bp, 2,000 bp, and 4,000 bp) and evaluated on a consistent 1,000 bp test dataset to ensure fair comparison. Performance analysis revealed that the 1,000 bp training length achieved optimal results (mean PearsonR: 0.806 ± 0.067), with significant performance degradation observed for both shorter sequences (250 bp: 0.616 ± 0.117; 500 bp: 0.767 ± 0.075) and longer sequences (2,000 bp: 0.706 ± 0.046; 4,000 bp: 0.709 ± 0.047). These results confirm that matching training and evaluation sequence lengths at 1,000 bp provides the optimal configuration for signal prediction tasks.

Supplementary Methods

Supplementary Method 1: Reference classical models

The encoder of reference classical models employ hierarchical one-dimensional (1D) convolution operations, which are product-sum operations defined between the input vector x and the kernel vector $k_{\text{classical}}$. For each output channel c, the 1D convolution computes:

$$f_c(i) = \sum_{d=0}^{C_{\text{in}}-1} \sum_{j=0}^{k_{\text{classical}}-1} w_{c,d,j} * x_{d,i+j} + b_c, \tag{1}$$

where $f_c(i)$ is the ith element of the cth channel of the output feature map, C_{in} is the number of input channels, $k_{\text{classical}}$ is the kernel size, $w_{c,d,j}$ is the kernel weight, $x_{d,i+j}$ is the input, and b_c is the bias term. The symbol $*$ represents the element-wise multiplication and summation.

Collectively, these operations form a 1D convolutional layer that transforms an input tensor of shape $L_{\text{in}} \times C_{\text{in}}$ to an output tensor of shape $L' \times C_{\text{out}}$, represented as $f : \mathbb{R}^{L_{\text{in}} \times C_{\text{in}}} \to \mathbb{R}^{L' \times C_{\text{out}}}$, where L_{in} is the input sequence length, C_{out} is the number of output channels and $L' = L_{\text{in}} - k_{\text{classical}} + 1$ is the output length.

Supplementary Method 2: QConv: Quantum Convolutional Layer

The Quantum Convolutional Layer (QConv) introduces a quantum-classical hybrid approach to extract features from sequential data. This section describes the theoretical foundation and implementation strategies of QConv, a generalized architecture that can be integrated within neural networks for enhanced representational capacity.

General Definition and Architecture

A Quantum Convolutional Layer aims to perform a convolution operation with a target kernel size k_{target} by transforming an input tensor into an output feature map:

$$f_{\text{QConv}} : \mathbb{R}^{L_{\text{in}} \times C_{\text{in}}} \to \mathbb{R}^{L_{\text{out}} \times C_{\text{out}}}, \tag{2}$$

where L_{in} is the input length, C_{in} is the number of input channels, $L_{\text{out}} = L_{\text{in}} - k_{\text{target}} + 1$ is the output length after convolution, and C_{out} is the number of output channels.

To achieve this transformation efficiently while leveraging quantum computational advantages, the QConv layer implements a three-stage processing pipeline:

1. Process A: Quantum convolution operation (qconv) on individual input channels
2. Process B: Feature map concatenation across channels
3. Process C: Classical complementary convolution for receptive field expansion

These processes work in sequence to transform the original input into the final output feature map, with each stage serving a specific role in the overall architecture.

Process A: qconv operation

Process A implements qconv, a quantum-based 1D convolution operation, inspired by Henderson et al.'s quanvolution [4]. Unlike classical convolution, qconv leverages quantum circuits to transform classical input data into feature representations while maintaining translation invariance.

For an n-qubit system, the qconv operation processes a single input channel and is defined as:

$$f_{\text{qconv}} : \mathbb{R}^{L_{\text{in}}} \to \mathbb{R}^{L' \times n}, \tag{3}$$

where $L' = L_{\text{in}} - k_{\text{quantum}} + 1$ is the intermediate output length, k_{quantum} is the quantum kernel size, and n is the number of qubits. In our approach, we set $k_{\text{quantum}} = n$ to match the number of qubits with the quantum kernel size.

The qconv operation consists of three sequential modules:

Encoding Module The encoding module transforms classical data into quantum states, enabling quantum information processing. Among various quantum encoding methods (angle encoding, amplitude encoding, basis encoding) [5], we adopt angle encoding for its prevalence in variational quantum algorithms.

In angle encoding, input data is encoded as rotation angles for single-qubit Y-axis rotation gates:

$$R_Y(\theta) = \begin{pmatrix} \cos(\theta/2) & -\sin(\theta/2) \\ \sin(\theta/2) & \cos(\theta/2) \end{pmatrix}. \tag{4}$$

For an n-qubit system, angle encoding represents a transformation $\mathbb{R}^n \to \mathbb{C}^{2^n}$. When processing sequential data with quantum kernel size k_{quantum} (where $k_{\text{quantum}} = n$ in our implementation), each input window element is encoded into a quantum state:

$$|x_i\rangle = R_Y(\pi x_i)|0\rangle = \cos(\frac{\pi}{2}x_i)|0\rangle + \sin(\frac{\pi}{2}x_i)|1\rangle \in \mathbb{C}^2, \quad i = 1, 2, \ldots, n. \tag{5}$$

The initial quantum state $|\psi_{\text{in}}\rangle$ is obtained as the tensor product of individual encoded states:

$$|\psi_{\text{in}}\rangle = \bigotimes_{i=1}^{n} |x_i\rangle \in \mathbb{C}^{2^n}. \tag{6}$$

This encoding maps classical information to an exponentially larger quantum state space, providing enhanced feature extraction capabilities [6].

Entanglement Module (Parameterized Unitary Transformation) In the entanglement module, a parameterized quantum circuit (PQC) $U(\theta)$ transforms the encoded quantum state:

$$|\psi_{\text{out}}\rangle = U(\theta)|\psi_{\text{in}}\rangle. \tag{7}$$

For an n-qubit system, $U(\theta)$ is a $2^n \times 2^n$ unitary matrix implementing a linear transformation $\mathbb{C}^{2^n} \to \mathbb{C}^{2^n}$. This operation generates quantum superpositions and entangled states, properties unique to quantum computation that enable exploration of complex feature interactions.

Decoding Module (Measurement and Feature Map Generation) The decoding module extracts classical information from quantum states through measurement operations. We calculate expectation values of the Pauli-Z operator for each qubit in the output state $|\psi_{\text{out}}\rangle$.

The Pauli-Z operator, defined as $\sigma_z = \begin{pmatrix} 1 & 0 \\ 0 & -1 \end{pmatrix}$, is applied to each qubit individually to obtain feature values:

$$f_i(\theta) = \langle\psi_{\text{out}}|\sigma_z^{(i)}|\psi_{\text{out}}\rangle = \langle\psi_{\text{in}}|U^\dagger(\theta)\sigma_z^{(i)}U(\theta)|\psi_{\text{in}}\rangle, \quad i = 1, 2, \ldots, n. \tag{8}$$

Here, $U^\dagger(\theta)$ represents the Hermitian conjugate of $U(\theta)$, and $\sigma_z^{(i)} = I \otimes \cdots \otimes \sigma_z \otimes \cdots \otimes I$ indicates that the Pauli-Z operator acts only on the i-th qubit, where I is the 2×2 identity matrix. Each feature value $f_i(\theta)$ ranges within $[-1, 1]$ due to the properties of Pauli-Z measurements.

By applying this process across all sliding windows of the input, we generate a structured feature map $F_{\text{A}} \in \mathbb{R}^{L' \times n}$ for each input channel.

Process B: Feature Map Concatenation

Process B integrates the feature maps F_{A} generated from individual input channels. Unlike classical convolution where input channels are summed per kernel, QConv concatenates F_A along the channel dimension [4, 7, 8].

For C_{in} input channels, each producing an $L' \times n$ feature map, Process B generates an intermediate output feature map $F_{\text{B}} \in \mathbb{R}^{L' \times (C_{\text{in}} \times n)}$. The feature map F_{B} depends on the trainable parameters θ within the PQC, which are optimized through gradient-based training using a classical optimizer.

Process C: Kernel Division Strategy for Effective Receptive Field

Process C applies a complementary classical convolution to the feature map F_{B} produced by Process B, generating the final output feature map $F_{\mathrm{C}} \in \mathbb{R}^{L_{\mathrm{out}} \times C_{\mathrm{out}}}$. This Kernel Division Strategy addresses computational limitations when implementing quantum circuits with large kernel sizes. While larger receptive fields are desirable for capturing extended patterns like TF binding motifs, classical simulation of quantum circuits becomes exponentially more resource-intensive as the number of qubits increases.

This strategy is inspired by Szegedy et al.'s Inception architecture [9], which proposed factorizing large convolutions into smaller sequential operations to reduce computational cost while maintaining expressivity. We adapt this principle to the quantum-classical hybrid context.

For a target effective quantum receptive field size r_{target}, the Kernel Division Strategy implements a hybrid approach:

$$r_{\mathrm{target}} = r_{\mathrm{quantum}} + r_{\mathrm{classical}} - 1, \tag{9}$$

where r_{quantum} is the real receptive field of the quantum circuit, and $r_{\mathrm{classical}}$ is the receptive field of the complementary classical convolution. Since the receptive field directly corresponds to the kernel size in our 1D convolution operations, this relationship can also be expressed in terms of kernel sizes:

$$k_{\mathrm{target}} = k_{\mathrm{quantum}} + k_{\mathrm{classical}} - 1. \tag{10}$$

This strategy partitions the convolution operation into:

1. A quantum convolution with kernel size k_{quantum} (Processes A and B)
2. A subsequent classical convolution with kernel size $k_{\mathrm{classical}}$ (Process C)

Through this division, the final output length L_{out} can be calculated as:

$$\begin{aligned} L_{\mathrm{out}} &= L' - k_{\mathrm{classical}} + 1 \\ &= (L_{\mathrm{in}} - k_{\mathrm{quantum}} + 1) - k_{\mathrm{classical}} + 1 \\ &= L_{\mathrm{in}} - (k_{\mathrm{quantum}} + k_{\mathrm{classical}} - 1) + 1 \\ &= L_{\mathrm{in}} - k_{\mathrm{target}} + 1. \end{aligned} \tag{11}$$

This confirms that our approach achieves the intended transformation from $\mathbb{R}^{L_{\mathrm{in}} \times C_{\mathrm{in}}} \to \mathbb{R}^{L_{\mathrm{out}} \times C_{\mathrm{out}}}$ where $L_{\mathrm{out}} = L_{\mathrm{in}} - k_{\mathrm{target}} + 1$. Here, C_{out} represents the number of different kernels (filters) in the complementary classical convolution operation. This approach allows QConv to achieve a large effective receptive field while maintaining computational feasibility in classical simulation environments.

QTFPred Implementation: QConv1

In QTFPred, we implement the first convolutional layer (QConv1) as a QConv layer with specific parameters for directly processing one-hot DNA sequence:

- Input: DNA sequence in one-hot encoding ($C_{\mathrm{in}} = 4$ for A, C, G, T)
- Quantum circuit: $n = 4$ qubits operating in $2^n = 16$ dimensional complex Hilbert space (the detailed structure shown in Supplementary Fig. 22 and Supplementary Method 9)
- Target receptive field: $r_{\mathrm{target}} = 16$ bp (sufficient to capture most TF binding motifs in JASPAR2024)
- Kernel Division configuration: $k_{\mathrm{quantum}} = 4$, $k_{\mathrm{classical}} = 13$
- Output channels: $C_{\mathrm{out}} = 64$

We selected a target receptive field of 16 bp based on analysis of JASPAR2024 motif length distribution (Supplementary Method 15), where 96.5 % of known TF binding motifs are 16 bp or shorter (median length 9.0 bp, mean 9.4 bp, standard deviation 3.3 bp).

Due to the exponential computational cost of simulating quantum circuits, we found that using $k_{\mathrm{quantum}} > 4$ led to prohibitively long training times in our experimental environment. The Kernel Division Strategy

enables us to achieve the desired 16 bp receptive field while keeping quantum simulation computationally tractable.

The subsequent network layers after QConv1 maintain the same structure as the reference classical models, facilitating direct performance comparisons.

Supplementary Method 3: Implementation

We implemented QTFPred using PennyLane [10] (a Python library for quantum circuit simulation) and PyTorch [11]. We used NVIDIA CUDA 12.1 for GPU simulation. PennyLane supports quantum circuit implementation and automatic differentiation of PQC variational parameters (Supplementary Method 12), facilitating the construction and training of quantum-classical hybrid models. We implemented the quantum convolutional layer using PennyLane's qml.qnn.TorchLayer module, which converts quantum circuit functions into PyTorch-compatible neural network layers.

The quantum convolution operations require sliding window processing of input vectors, which could become a computational bottleneck. To accelerate this process, we leveraged PennyLane's default.qubit simulator vectorization capabilities. This approach allows simultaneous evaluation of the same quantum circuit across multiple input data points while sharing the same parameters, as represented by:

$$[f(x_1, \theta), f(x_2, \theta), \ldots, f(x_n, \theta)], \tag{12}$$

where $f(x_i, \theta)$ denotes the expectation value from evaluating the quantum circuit with parameters θ on input vector x_i. By applying this vectorization technique, we transformed what would otherwise be n sequential quantum circuit evaluations into a single parallel operation [10, 12], accelerating the forward propagation process.

Supplementary Method 4: Comparison methods

We benchmarked the performance of our method against several existing state-of-the-art methods for two tasks: binary TFBS prediction and signal prediction. For the binary TFBS prediction task, we used the reference classical model FCNA and FCN.

FCNA is a model that uses fully convolutional neural networks (FCN) [13] and global average pooling (GAP) to predict TFBS and motifs from ChIP-seq data.

In the signal prediction task, we used the reference classical model FCNsignal and BPNet [14] to evaluate the regression performance of ChIP-seq signals. FCNsignal has a structure similar to FCNA but differs in that a bidirectional gated recurrent unit (GRU) is added to capture long-term dependencies within sequence-specific features before GAP in the bottleneck layer, and the head is constructed for signal prediction. BPNet is a dilated CNN model proposed to predict the base resolution ChIP-nexus binding profiles of pluripotent TFs.

Supplementary Method 5: Tuning of hyperparameters

We optimized QTFPred's hyperparameters using Optuna [15], which implements the Tree-structured Parzen Estimator (TPE) algorithm for black-box optimization. We used IOU maximization as the objective function for binary classification and MSE minimization for signal prediction.

For binary prediction tuning, we created a merged dataset from four TFs (USF1, ETS1, JUND, E2F6) in the A549 cell line, with 50 % of the 32,465 examples (sequence length 500 bp) used for evaluation. For signal prediction tuning, we merged sequences from three TFs (ZNF740, FOSL1, GATA2) in the K562 cell line, with 30 % of the 34,734 examples (sequence length 1,000 bp) used for evaluation. To prevent data leakage, the specific TFs used for tuning were excluded from subsequent training and evaluation. Each hyperparameter trial involved training the model for 10 epochs on the training portion of these datasets, then calculating the objective function value using the designated evaluation portion.

Sequence homology or motif similarity between tuning and evaluation datasets can lead to indirect data leakage, potentially inflating performance estimates during hyperparameter optimization. To assess this risk for binary classification tasks, we evaluated motif similarity between the four tuning TFs and 26 unique evaluation TFs using TOMTOM analysis (Supplementary Fig. 31), which revealed that only FOSL2 among evaluation TFs showed high motif similarity to JUND from the tuning set (similarity score 7.8) with highly similar consensus sequences (JUND: GATGACTCATC vs FOSL2: GATGACTCATCC), while the

second-highest similarity between USF1 and ATF3 (similarity score 2.8) showed different consensus sequences (USF1: GGTCACGTGACC vs ATF3: GGTGACGTGAT), indicating that the risk of indirect data leakage is limited to one specific TF and does not substantially affect the broader evaluation of QTFPred's generalization capability for binary classification tasks.

For baseline binary prediction models (FCN and FCNA), we adopted the hyperparameters from their original publication [16]. For signal prediction models (BPNet and FCNsignal), we only optimized parameters not specified in the work [17] (learning rate, AdamW weight decay, beta1, beta2) using our tuning dataset.

The complete list of hyperparameters used in our study is provided in Supplementary Table 3 and Supplementary Table 4.

Supplementary Method 6: Downsampling experiment

To evaluate how model performance varies with different amounts of training data, particularly in ultra-low data regimes, we designed a comprehensive down-sampling experiment with five TFs (GM12878: CTCF, EBF1, BHLHE40; K562: ZBTB33, JUND) possessing abundant binding regions. For each TF, we created nested training datasets of varying sizes through random down-sampling, ensuring that larger datasets fully included smaller ones to maintain consistency across comparisons.

We targeted specific peak counts of 250, 500, 750, 1,000, 1,500, 3,000, 6,000, 12,000, 24,000, and full peaks for our analysis. Since our training data contains both positive and negative samples in approximately equal proportions (as described in the Data and pre-processing section), we made the reasonable approximation that positive samples (actual peaks) constitute roughly half of the total dataset size. Following this assumption, we generated training datasets of 500, 1,000, 1,500, 2,000, 3,000, 6,000, 12,000, 24,000, 48,000, and full dataset sizes to achieve our target peak counts.

For each down-sampled dataset, we performed five independent training trials for both signal prediction and binary classification tasks. For signal prediction, we compared QTFPred against FCNsignal, while for binary classification, we compared QTFPred against FCNA. The binary classification experiments used eight TFs from GM12878 and MCF7 cell lines (GM12878: BCL11A, PBX3, SP1, TCF12, YY1; MCF7: CEBPB, CTCF, NR2F2) to evaluate model robustness across different prediction tasks and validate the generalizability of our findings from signal prediction to classification scenarios.

Supplementary Method 7: Benchmark of training time and peak memory

To quantitatively evaluate the computational resources required by each model, we conducted comprehensive benchmarking experiments comparing BPNet, FCNsignal, and QTFPred.

We measured the following metrics:

- Training time per epoch
- Peak GPU memory usage during training
- Peak CPU memory usage during training
- Average inference time per batch

We conducted experiments using six TF datasets from the HeLa-S3 cell line with 1,000 bp sequences, selecting two TFs from each peak group: low peak group (ELK1, ELK4), middle peak group (REST, MAZ), and high peak group (JUND, CTCF). All benchmarking was performed in a standardized computational environment using a single NVIDIA H100 PCIe GPU with 80 GB memory (CUDA 12.4) on an Ubuntu 22.04.5 LTS system with 755 GB of RAM and dual AMD EPYC 7302 16-Core Processors.

To ensure statistical reliability, we executed 10 independent trials for each model-TF combination with a consistent batch size of 100 across all models. Each trial consisted of 5 epochs, with memory usage recorded at regular intervals (every 10 batches) throughout training. The benchmarking procedure included an initial warm-up phase (3 batches) to stabilize GPU memory allocation before beginning measurements. We cleared GPU cache between trials to ensure consistent starting conditions. All experiments were orchestrated using

a SLURM job scheduling system that managed dependencies between tasks, ensuring systematic execution of all model-TF combinations while maintaining consistent hardware conditions throughout the evaluation process.

Supplementary Method 8: Ablation study on the effect of qubit counts

To systematically investigate the impact of quantum computation on model performance, we conducted an ablation study examining how qubit counts in the quantum convolutional layer affect prediction accuracy. We constructed a series of models using our Kernel Division Strategy by replacing the first convolutional layer of the optimized FCNsignal model with quantum convolutional layers containing 1, 2, 3, or 4 qubits.

For each configuration, we maintained a consistent effective receptive field size of the quantum convolutional layer across all models by adjusting the kernel size of the complementary classical convolution according to the number of qubits used. Specifically, we implemented the following configurations:

- 1 qubit ($k_{\text{quantum}} = 1, k_{\text{classical}} = 16$)
- 2 qubits ($k_{\text{quantum}} = 2, k_{\text{classical}} = 15$)
- 3 qubits ($k_{\text{quantum}} = 3, k_{\text{classical}} = 14$)
- 4 qubits ($k_{\text{quantum}} = 4, k_{\text{classical}} = 13$)

This approach ensures that the total receptive field remains 16 bp for all models, matching the receptive field of the original FCNsignal model's first convolutional layer.

All hyperparameters except batch size were kept consistent with the optimized FCNsignal model to ensure that performance differences could be attributed solely to the expressive power of the quantum circuit. Due to GPU memory constraints when simulating quantum circuits, we reduced the batch size from the original FCNsignal's 500 to 66 for all ablation experiments.

Supplementary Method 9: Detail of parameterized quantum circuit

We implemented a structured circuit design rather than a random circuit configuration [18–21], as structured designs offer greater interpretability [19, 22–24] (Supplementary Fig. 22). Our circuit architecture incorporates data re-uploading [24–26], a technique that enhances model expressivity by encoding input data multiple times throughout the circuit. This approach provides redundancy in the encoding process and enables greater generalization performance.

For entanglement generation, we employed a cyclic code structure inspired by [23], using control gates and parameterized unitary transformations. The cyclic code block B is defined as:

$$B = \prod_{j=0}^{N-1} R_j \prod_{i=0}^{N-1} C_{i,(i+r) \bmod N}, \tag{13}$$

where $N = 4$ is our qubit count, $C_{i,(i+r) \bmod N}$ represents CNOT gates with control qubit i and target qubit $(i + r) \bmod N$, and $r = 1$ is the connectivity range parameter. Each R_j is a parameterized rotation gate defined as:

$$\begin{aligned} R_j(\phi_j, \theta_j, \omega_j) &= R_Z(\omega_j) R_Y(\theta_j) R_Z(\phi_j) \\ &= \begin{pmatrix} e^{-i(\phi_j+\omega_j)/2} \cos(\theta_j/2) & -e^{i(\phi_j-\omega_j)/2} \sin(\theta_j/2) \\ e^{i(\phi_j-\omega_j)/2} \sin(\theta_j/2) & e^{i(\phi_j+\omega_j)/2} \cos(\theta_j/2) \end{pmatrix}, \end{aligned} \tag{14}$$

where ϕ_j, θ_j, and ω_j are trainable rotation parameters, and $R_Z(\alpha) = \begin{pmatrix} e^{-i\alpha/2} & 0 \\ 0 & e^{i\alpha/2} \end{pmatrix}$ is the Z-axis rotation matrix. Our PQC contains 36 trainable parameters.

Supplementary Method 10: Selection of motif calculation algorithm

We adopted the filter activation approach [2, 17] rather than gradient back-propagation strategies (such as DeepLIFT[27] and TF-MoDISco[28]) for motif detection. While gradient back-propagation calculates each input's contribution to the final output throughout the entire network, our objective was to specifically isolate representations from the quantum convolutional layer. The filter activation approach allows direct

observation of this layer's output, enabling us to examine QTFPred's learned feature representations without influence from deeper network components.

Supplementary Method 11: Reproducible unannotated motif

To identify highly informative and reproducible unannotated motifs from QTFPred predictions, we implemented a two-stage filtering process. For specificity assessment, we established a threshold at the lower quartile (Q1) of information content (IC) distribution among annotated motifs for each primary TF, selecting only unannotated motifs exceeding this threshold to maintain comparable specificity to known motifs rather than representing random noise. For reproducibility assessment, we evaluated persistence across five independent trials and retained only motifs appearing in identical patterns across at least four of the five trials based on TOMTOM annotation, effectively eliminating spurious patterns while preserving genuinely consistent sequence features.

Supplementary Method 12: Computation of gradient

For gradient computation in QTFPred, we utilized PennyLane's default.qubit state vector simulator with the backpropagation differentiation method, selected for both its computational efficiency and compatibility with parameter broadcasting requirements in our implementation. Backpropagation is exclusively applicable to quantum circuit simulators as physical quantum hardware implementations cannot preserve intermediate quantum states due to wave function collapse upon measurement, preventing the backward propagation of gradients. For physical quantum hardware implementation of QTFPred, analytical methods such as the parameter-shift rule [29, 30] would be necessary.

Supplementary Method 13: The distribution of distances between motifs

To characterize physical binding relationships between transcription factor pairs, we analyzed inter-motif distances within ENCODE ChIP-seq peak regions. From each peak center, we extracted sequences spanning $\pm$600 bp (accommodating both the 1,000 bp training sequence length and $\pm$100 bp random noise) and identified motif positions using FIMO [31] with p-value $<$ 1e-3 against the JASPAR CORE 2024 database. Following the approach of Avsec et al. [14], we separately analyzed four possible orientation combinations of TF pairs (++, +−, −+, −−), where each sign represents the respective TF's strand orientation. For each orientation combination, we calculated the absolute distance between central positions of motif pairs within each peak region, selecting the minimum distance value per region to generate frequency distributions for analysis.

Supplementary Method 14: Statistical detection of specific distances

We developed a peak detection algorithm based on the Savitzky-Golay filter [32] to statistically identify specific binding distances distinguishable from background noise, applying it to raw distance distribution data with window length of 5 and polynomial degree of 3 to reduce noise. Our multi-stage filtering approach evaluated peaks using three statistical criteria: (1) prominence—measured as the height difference between peak and surrounding valley, with a threshold of 10 % of maximum distribution value; (2) width—requiring a minimum width of 2 to exclude narrow noise peaks; and (3) signal-to-noise ratio—requiring peak height to be at least twice the surrounding noise level (threshold of 2.0) to ensure reliability. This comprehensive approach enabled robust identification of statistically significant distance patterns between transcription factor binding sites.

Supplementary Method 15: Distribution Analysis of ENCODE Peaks and JASPAR Motif Lengths

We collected ChIP-seq data through the ENCODE API by accessing all TF ChIP-seq experiments registered for Homo sapiens across all cell lines (as of October 1, 2024). After retrieving experimental IDs for matching experiments, we downloaded peak files in bed format filtered by IDR scores and tabulated peak counts for each dataset.

For motif length distribution analysis, we examined the JASPAR2024 CORE non-redundant PFM database, counting matrix rows for each transcription factor motif to generate the length distribution histogram.

Supplementary Method 16: Statistical analysis

All statistical analyses were carried out using Python 3. For statistical hypothesis testing, we used Welch's t-test from the SciPy stats module [33].

For the TF detection binary heatmap analysis, correlation coefficients between primary TFs were calculated using NumPy's corrcoef function [34]. Hierarchical clustering was performed using SciPy's hierarchy module with the Ward method (linkage function with method='ward' option). We obtained the optimal leaf ordering from the hierarchy.dendrogram function to arrange similar TFs adjacently in the correlation matrix visualization.

Supplementary References

1. Lambert SA, Jolma A, Campitelli LF, et al. The human transcription factors. Cell 2018; 172:650–65
2. Kelley DR, Snoek J, and Rinn JL. Basset: learning the regulatory code of the accessible genome with deep convolutional neural networks. Genome research 2016; 26:990–9
3. Maslova A, Ramirez RN, Ma K, et al. Deep learning of immune cell differentiation. Proceedings of the National Academy of Sciences 2020; 117:25655–66
4. Henderson M, Shakya S, Pradhan S, et al. Quanvolutional neural networks: powering image recognition with quantum circuits. Quantum Machine Intelligence 2020 Jun; 2(1):2
5. Schuld M and Petruccione F. Supervised learning with quantum computers. Vol. 17. Springer, 2018
6. Schuld M and Killoran N. Quantum Machine Learning in Feature Hilbert Spaces. Physical Review Letters 2019 Feb; 122(4)
7. Shi S, Wang Z, Shang R, et al. Hybrid quantum-classical convolutional neural network for phytoplankton classification. Frontiers in Marine Science 2023; 10
8. Mattern D, Martyniuk D, Willems H, et al. Variational quanvolutional neural networks with enhanced image encoding. arXiv preprint arXiv:2106.07327 2021
9. Szegedy C, Vanhoucke V, Ioffe S, et al. Rethinking the Inception Architecture for Computer Vision. 2015
10. Bergholm V, Izaac J, Schuld M, et al. PennyLane: Automatic differentiation of hybrid quantum-classical computations. 2022
11. Paszke A, Gross S, Massa F, et al. Pytorch: An imperative style, high-performance deep learning library. Advances in neural information processing systems 2019; 32
12. Zhang SX, Allcock J, Wan ZQ, et al. TensorCircuit: a Quantum Software Framework for the NISQ Era. Quantum 2023 Feb; 7:912
13. Long J, Shelhamer E, and Darrell T. Fully Convolutional Networks for Semantic Segmentation. 2015
14. Avsec Ž, Weilert M, Shrikumar A, et al. Base-resolution models of transcription-factor binding reveal soft motif syntax. Nature genetics 2021; 53(3):354–66
15. Akiba T, Sano S, Yanase T, et al. Optuna: A Next-generation Hyperparameter Optimization Framework. 2019
16. Zhang Y, Wang Z, Zeng Y, et al. High-resolution transcription factor binding sites prediction improved performance and interpretability by deep learning method. Briefings in Bioinformatics 2021; 22(6):bbab273
17. Zhang Q, He Y, Wang S, et al. Base-resolution prediction of transcription factor binding signals by a deep learning framework. PLoS computational biology 2022; 18(3):e1009941
18. Cerezo M, Arrasmith A, Babbush R, et al. Variational quantum algorithms. Nature Reviews Physics 2021 Sep; 3(9):625–44

19. Sim S, Johnson PD, Aspuru-Guzik A, et al. Expressibility and Entangling Capability of Parameterized Quantum Circuits for Hybrid Quantum-Classical Algorithms. Advanced Quantum Technologies 2019 Dec; 2(12):1900070

20. Bharti K, Cervera-Lierta A, Kyaw TH, et al. Noisy intermediate-scale quantum algorithms. Reviews of Modern Physics 2022 Mar; 94(1)

21. McClean JR, Boixo S, Smelyanskiy VN, et al. Barren plateaus in quantum neural network training landscapes. Nature Communications 2018; 9

22. Kandala A, Mezzacapo A, Temme K, et al. Hardware-efficient variational quantum eigensolver for small molecules and quantum magnets. Nature 2017; 549:242–6

23. Schuld M, Bocharov A, Svore KM, et al. Circuit-centric quantum classifiers. Phys. Rev. A 2020 Mar; 101(3):32308

24. Schuld M, Sweke R, and Meyer JJ. Effect of data encoding on the expressive power of variational quantum-machine-learning models. Physical Review A 2021 Mar; 103(3):032430

25. Pérez-Salinas A, Cervera-Lierta A, Gil-Fuster E, et al. Data re-uploading for a universal quantum classifier. Quantum 2020 Feb; 4:226

26. Wach NL, Rudolph MS, Jendrzejewski F, et al. Data re-uploading with a single qudit. Quantum Machine Intelligence 2023 Dec; 5(2):1–12

27. Shrikumar A, Greenside P, and Kundaje A. Learning Important Features Through Propagating Activation Differences. *Proceedings of the 34th International Conference on Machine Learning.* Ed. by Precup D and Teh YW. Vol. 70. Proceedings of Machine Learning Research. PMLR, 2017 Jun :3145–53

28. Shrikumar A, Tian K, Avsec Ž, et al. Technical note on transcription factor motif discovery from importance scores (TF-MoDISco) version 0.5. 6.5. arXiv preprint arXiv:1811.00416 2018

29. Mitarai K, Negoro M, Kitagawa M, et al. Quantum circuit learning. Physical Review A 2018; 98(3):32309

30. Schuld M, Bergholm V, Gogolin C, et al. Evaluating analytic gradients on quantum hardware. Phys. Rev. A 2019 Mar; 99(3):032331

31. Grant CE, Bailey TL, and Noble WS. FIMO: scanning for occurrences of a given motif. Bioinformatics 2011; 27:1017–8

32. Savitzky A and Golay MJ. Smoothing and differentiation of data by simplified least squares procedures. Analytical chemistry 1964; 36:1627–39

33. Virtanen P, Gommers R, Oliphant TE, et al. SciPy 1.0: Fundamental Algorithms for Scientific Computing in Python. Nature Methods 2020; 17:261–72

34. Harris CR, Millman KJ, Walt SJ van der, et al. Array programming with NumPy. Nature 2020 Sep; 585:357–62
